# Exploring high-resolution chromatin interaction changes and functional enhancers of myogenic marker genes during myogenic differentiation

**DOI:** 10.1016/j.jbc.2022.102149

**Published:** 2022-07-02

**Authors:** Keren Long, Xiaokai Li, Duo Su, Sha Zeng, Hengkuan Li, Yu Zhang, Biwei Zhang, Wenying Yang, Penghao Li, Xuemin Li, Xun Wang, Qianzi Tang, Lu Lu, Long Jin, Jideng Ma, Mingzhou Li

**Affiliations:** 1 Institute of Animal Genetics and Breeding, College of Animal Science and Technology, Sichuan Agricultural University, Chengdu, China; 2 Jinxin Research Institute for Reproductive Medicine and Genetics, Chengdu Xi’nan Gynecology Hospital Co, Ltd, Chengdu, Sichuan, China

**Keywords:** myogenesis, 3D chromatin, 4C-seq, promoter–enhancer interaction, transcription regulation, 3C, chromosome conformation capture, 4C-seq, circularized chromosome conformation capture sequencing, ChIP-seq, chromatin immunoprecipitation sequencing, DEG, differentially expressed gene, FI, fusion index, GO, Gene Ontology, GSEA, Gene Set Enrichment Analysis, Hi-C, high-throughput chromatin conformation capture, KEGG, Kyoto Encyclopedia of Genes and Genomes, MB, myoblast, MT, myotube, PCHi-C, promoter capture Hi-C, qRT-PCR, quantitative RT-PCR, SDIS, significant differential interaction site, sgRNA, single guide RNA, SIS, significant interaction site, TF, transcription factor

## Abstract

Skeletal muscle differentiation (myogenesis) is a complex and highly coordinated biological process regulated by a series of myogenic marker genes. Chromatin interactions between gene’s promoters and their enhancers have an important role in transcriptional control. However, the high-resolution chromatin interactions of myogenic genes and their functional enhancers during myogenesis remain largely unclear. Here, we used circularized chromosome conformation capture coupled with next generation sequencing (4C-seq) to investigate eight myogenic marker genes in C2C12 myoblasts (C2C12-MBs) and C2C12 myotubes (C2C12-MTs). We revealed dynamic chromatin interactions of these marker genes during differentiation and identified 163 and 314 significant interaction sites (SISs) in C2C12-MBs and C2C12-MTs, respectively. The interacting genes of SISs in C2C12-MTs were mainly involved in muscle development, and histone modifications of the SISs changed during differentiation. Through functional genomic screening, we also identified 25 and 41 putative active enhancers in C2C12-MBs and C2C12-MTs, respectively. Using luciferase reporter assays for putative enhancers of *Myog* and *Myh3*, we identified eight activating enhancers. Furthermore, dCas9-KRAB epigenome editing and RNA-Seq revealed a role for *Myog* enhancers in the regulation of *Myog* expression and myogenic differentiation in the native genomic context. Taken together, this study lays the groundwork for understanding 3D chromatin interaction changes of myogenic genes during myogenesis and provides insights that contribute to our understanding of the role of enhancers in regulating myogenesis.

Myogenesis *in vivo* is a complex biological process involving the commitment of embryonic precursors to the myogenic lineage, myoblast (MB) proliferation, and their eventual differentiation into skeletal muscle fibers. The terminal differentiation of MBs, including the proliferation, differentiation, and fusion of myocytes into multinucleated myotubes (MTs), is essential for the growth and development of skeletal muscle (1, 2). Myogenesis is associated with the temporal and spatial expression of muscle-specific genes. *Myod1* and *Myog* are critical transcription factor (TF) genes involved in skeletal muscle differentiation (3, 4, 5, 6). They cooperate with the myocyte enhancer factor-2 (MEF2A, MEF2B, MEF2C, and MEF2D) family of TFs to activate the expression of most myogenesis-related genes and promote the differentiation of MBs (7, 8, 9) such as the myosin heavy chain (*MHC*) gene families (10) and MB fusion factor (*Mymk*) (5, 6, 11). The molecular mechanisms involved in how myogenic genes regulate myogenesis have been extensively studied; however, few studies have focused on changes in 3D chromatin interactions between myogenic genes and their functional enhancers during myogenesis.

3D chromatin interactions in the nucleus are thought to have a fundamental role in gene regulation by facilitating or restricting regulatory element interactions such as promoter and enhancer interactions. Gene activation and inactivation are linked to dramatic changes in chromatin interactions. The development of chromosome conformation capture (3C) technology and its variations, including circularized chromosome conformation capture sequencing (4C-seq), high-throughput chromatin conformation capture (Hi-C), and promoter capture Hi-C (PCHi-C) have uncovered the principles of higher-order chromatin structures and identified chromatin contacts at different genome structural levels. Compared with Hi-C and PCHi-C, 4C-seq enables the detection of single promoter interactions at a higher resolution (1–2 kb) and lower sequencing depth (12). Previous studies revealed that the expression of myogenic genes was regulated by chromatin interactions between promoters and enhancers during myogenesis (13, 14, 15). However, because of the low resolution of current Hi-C and PCHi-C data, little is known about the dynamics of such promoter–enhancer interactions and the precise localization of enhancer sites at high resolution. Therefore, deciphering changes in the chromatin interactions between myogenic genes and their functional enhancers remains a major challenge.

The transcriptional regulation of genes is a key factor in gene expression in general. Enhancers are critical *cis*-regulatory elements that control spatial and temporal gene expression by recruiting specific TFs in a sequence-specific manner (16). Enhancers are typically located upstream (5′), downstream (3′), or in the intron of the gene that they regulate, and they can also be located distant from their target genes (17). Chromatin modifications of H3K27ac, H3K4me1, and H3K4me2 are effective markers to identify enhancers, and the combination of H3K27ac and H3K4me1 is widely used for the identification of active enhancers (18, 19, 20). Enhancers regulate cell type–specific gene expression through long-range chromatin interactions. For example, Shyamsunder *et al.* found that the +6 kb enhancer of the *Cebpe* gene is essential for granulocytic differentiation and that its germline deletion reduced the expression levels of *Cebpe* and significantly inhibited granulocytic differentiation (21). The Sonic hedgehog (*Shh*) gene is regulated by an enhancer located 1 Mb distant in an intron of the unrelated *Lmbr1* gene and point mutations of the enhancer segregated with polydactyly in humans and mice (22, 23). In terms of muscle cell differentiation, several studies have reported that interactions between enhancers and promoters affected cell differentiation (13, 14, 15). However, to date, how enhancer–promoter interactions activate myogenic gene expression to myogenic differentiation is poorly understood, although this information is required to understand the underlying differentiation mechanisms involved.

Here, we used 4C-seq to explore the chromatin interactions of eight myogenic marker genes (*Myod1*, *Myog*, *Mef2a*, *Mef2b*, *Mef2d*, *Myh2*, *Myh3*, and *Mymk*) that are important for the terminal differentiation of MBs to MTs. We found a change in the dynamic chromatin interactions of the myogenic marker genes during C2C12-MB differentiation and identified the significant interaction sites (SISs) of C2C12-MBs and C2C12-MTs. We used dCas9-Krüppel-associated box (KRAB) and RNA-Seq to study the role of *Myog* enhancers in target gene regulation and myogenic differentiation. These findings enhance our understanding of the molecular regulation of myogenesis associated with the promoter–enhancer chromatin interactions.

## Results

### Interactome characterization of eight myogenic marker genes during C2C12 MB differentiation

We induced C2C12-MBs to differentiate into C2C12-MTs *in vitro* by culture with 2% horse serum for 5 days (Fig. S2, *A*–*C*). Giemsa staining showed the presence of dark purple-stained MT cytoplasm and pink multinuclei in differentiated C2C12-MBs (Fig. S2*A*). Immunofluorescence staining revealed the high expression of myosin heavy chain (MHC) protein (markers of MB differentiation) in differentiated C2C12-MBs (Fig. S2*A*). We observed significant changes in cell morphology and visible MT formation in differentiated C2C12-MBs, with a high fusion index (FI) of 70% (Fig. S2, *A* and *B*). The mRNA expressions of eight well-characterized myogenic marker genes (*Myod1*, *Myog*, *Mef2a*, *Mef2b*, *Mef2d*, *Myh2*, *Myh3*, and *Mymk*) were evaluated in C2C12-MBs and differentiated C2C12-MBs by quantitative RT-PCR (qRT-PCR). Compared with C2C12-MBs, the expression levels of the myogenic marker genes after differentiation were upregulated significantly (*p* < 0.005) (Fig. S2*C*). These results suggested C2C12-MBs were successfully induced to differentiate into MTs *in vitro* and highlighted the dynamic mRNA expression of the myogenic marker genes during differentiation.

To reveal genome-wide chromatin interactions of the myogenic marker genes during myogenic differentiation, we performed 4C-seq experiments with the promoters of eight genes. Overall, we generated 32 libraries for the eight genes in C2C12-MBs and C2C12-MTs with two replicates each. We obtained ∼201.37 M filtered reads with an average of 6.29 M reads for each 4C data, and 57.55% to 85.95% of the total reads in 32 datasets were distributed on the *cis*-chromosome (Fig. S3*A*). This conforms to the “*cis*/overall ratio of > 40%” criteria proposed by Van De Werken *et al.* (24), indicating the good experimental quality. In addition, 26 out of 32 datasets showed a capt100 kb > 40%, indicating the high complexity and reproducibility of the 4C datasets (Fig. S3*B*). All 4C datasets had cov1Mb > 50%, and 87.5% (28/32) of the 4C datasets had cov1Mb > 60%, indicating that most reads were located within 1 Mb of the viewpoint (Fig. S3*C*). Detailed quality metrics are provided in Table S7.

**Figure 1:**
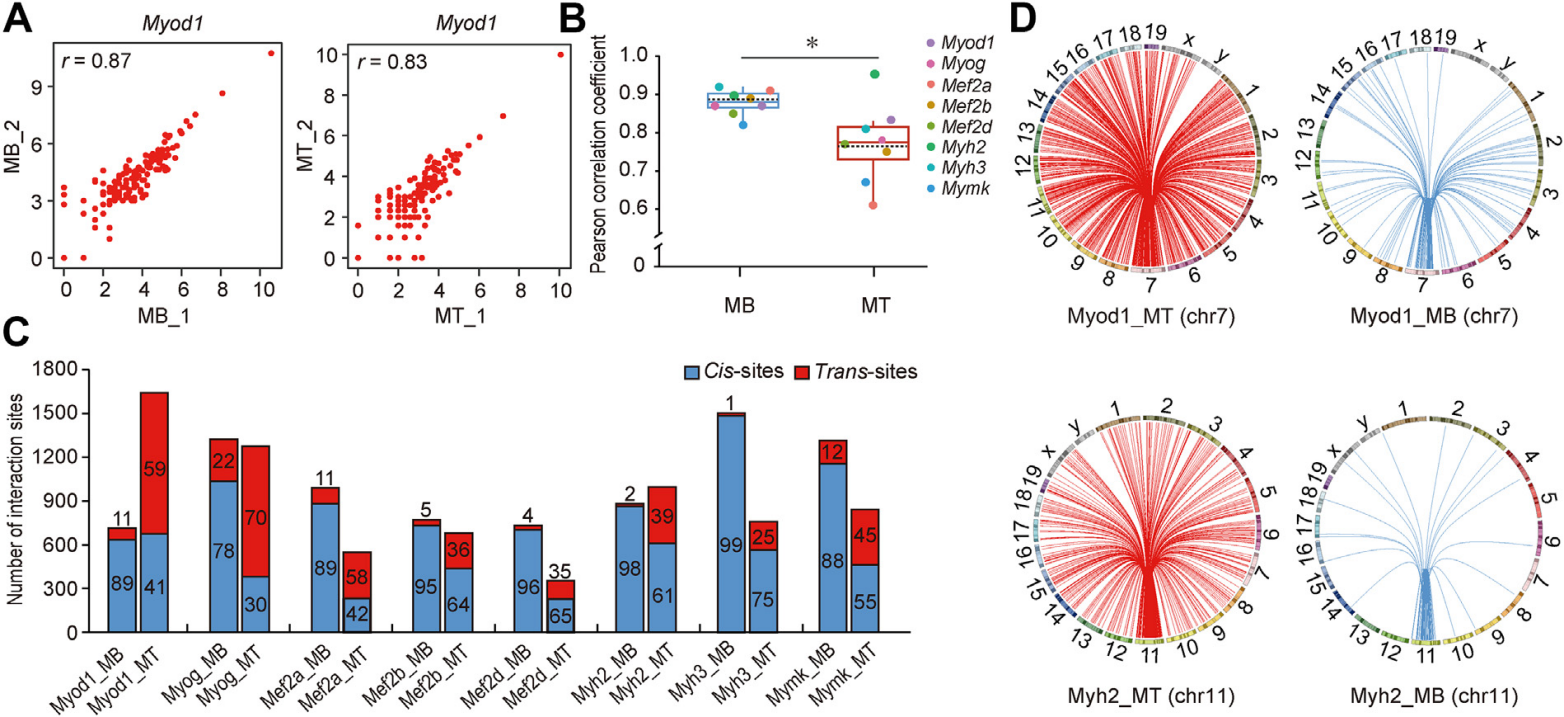
**The chromatin interactions characterization of eight myogenic marker genes.***A*, scatter plot showing interactions of *Myod1* in C2C12-MBs and C2C12-MTs. The number of interaction sites (in Log_2_) per 1 Mb *cis* in two replicates were plotted. The Pearson correlation coefficient is shown in the panel. *B*, the paired *t* test of Pearson correlation coefficient of the myogenic marker genes in C2C12-MBs and C2C12-MTs. *C*, the number of reliable interaction sites and the ratio of *cis*-/*trans*-interaction of the myogenic marker genes in C2C12-MBs and C2C12-MTs. The number inside or above the column indicates the percentage of *cis*-/*trans*-interaction sites. *D*, circos plot showing genome-wide interaction of *Myod1* and *Myh2* in C2C12-MTs and C2C12-MBs. Chromosomes are shown in a circular orientation. The numbers and letters above the circle indicate the chromosomes’ names. Results are expressed as mean ± SD (n = 3). ∗*p* < 0.05. MB, myoblast; MT, myotube.

We used r3Cseq to identify the interaction sites of each 4C data with a continuous nonoverlapping 2 kb window and counted the number and ratio of *cis*- and *trans*-interaction sites (Table S8). Then, we evaluated the reproducibility of intrachromosomal interactions between replicates by counting *cis*-interactions in every 1 Mb genomic bin. The Pearson correlation coefficient was 0.61 to 0.97 for each gene in C2C12-MBs and C2C12-MTs (Figs. 1*A* and S4), indicating good consistency between replicates. The paired *t* test revealed significantly higher correlation coefficients in C2C12-MBs than in C2C12-MTs (*p* < 0.05) (Fig. 1*B*), which may be related to the higher heterogeneity and complexity of C2C12 MB differentiation (25, 26).

To identify the reliable interaction sites, we overlapped interaction sites between replicates using r3Cseq. We identified 353 to 1641 interaction sites after overlapping replicates in C2C12-MBs and C2C12-MTs (Fig. 1*C*), which is comparable with other 4C-seq studies (27, 28, 29). In addition, more interaction sites were observed in C2C12-MBs for most myogenic marker genes except the *Myod1* and *Myh2* genes (Figs. 1, *C* and *D* and S5). For example, the number of interaction sites of *Myod1*, *Mef2d*, and *Myh3* genes increased more than twofold in C2C12-MBs compared with C2C12-MTs. Next, we counted the number of *cis*- and *trans*-interaction sites. Interestingly, all genes in C2C12-MTs had a higher *trans*-interaction ratio than in C2C12-MBs. Among them, the three most changed genes were *Myod1* (10.92% in C2C12-MBs, 58.81% in C2C12-MTs), *Myog* (21.77% in C2C12-MBs, 70.00% in C2C12-MTs), and *Mef2a* (11.00% in C2C12-MBs, 57.56% in C2C12-MTs). These results indicated the interaction divergence and high complexity of the chromatin interaction model of the myogenic marker genes during C2C12 MB differentiation.

### Chromatin interaction alterations of myogenic marker genes during C2C12 MB differentiation

**Figure 2:**
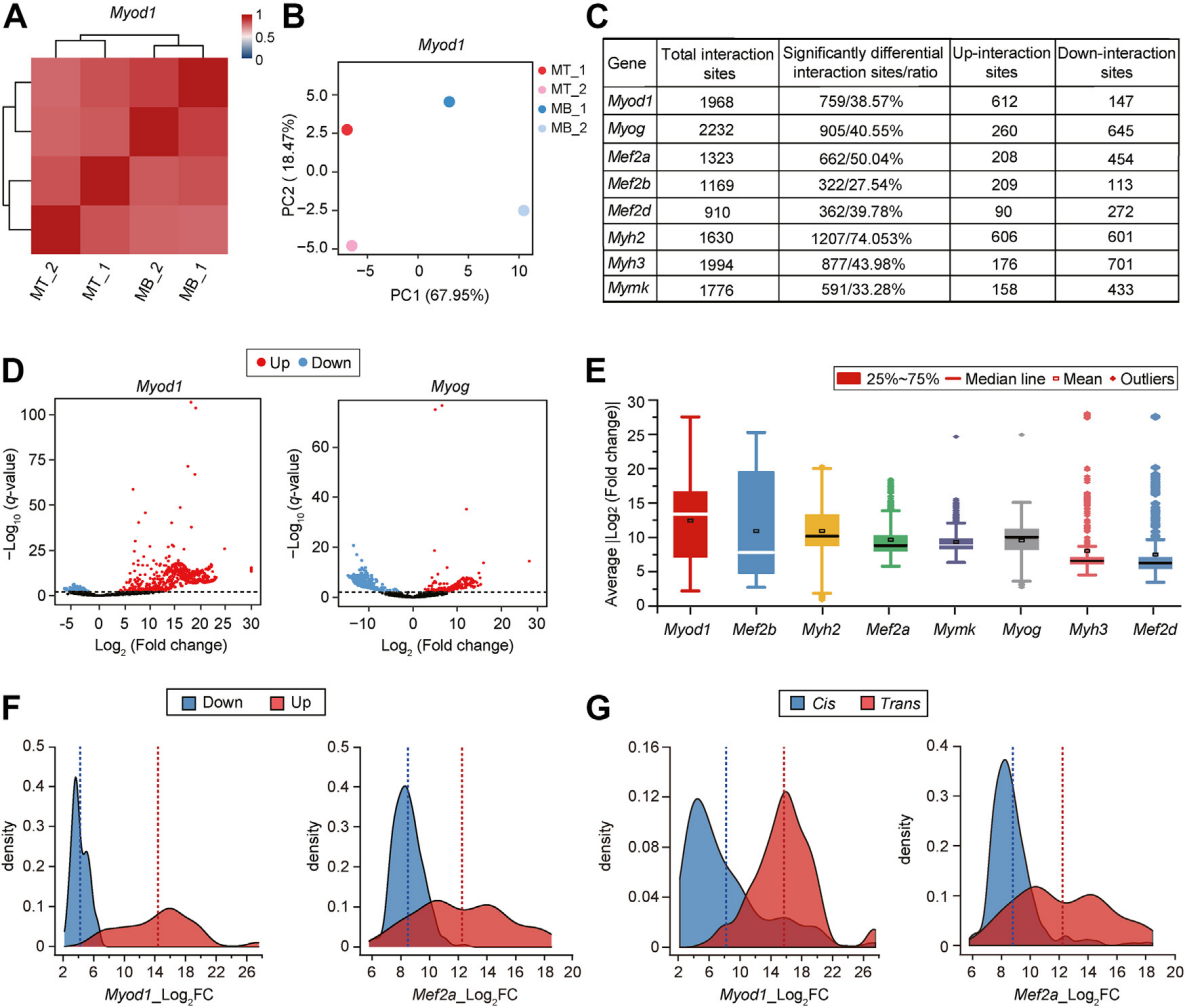
**Chromatin interaction alterations of the myogenic marker genes.***A*, heatmap showing the clustering of *Myod1* interactions in C2C12-MBs and C2C12-MTs. The color scale indicates the degree of correlation (*blue*, low correlation; *red*, high correlation). *B*, principal component analysis (PCA) of *Myod1* interactions in C2C12-MBs and C2C12-MTs. Each point represents a sample. The percentages on each axis represent the percentages of variation explained by the principal components. The clustering and PCA were generated using the Pearson correlation coefficient of interaction sites per1Mb *cis*. *C*, the SDISs of eight myogenic marker genes between C2C12-MTs and C2C12-MBs. Differential interaction was analyzed using the DESeq2 with the “ashr” algorithm (cutoff: *q*-value ≤ 0.01). *D*, volcano plot of SDISs of *Myod1* and *Myog*. The threshold of SDISs in the volcano plot was -Log_10_ (*q*-value) ≥ 2. *Red* and *blue* indicate significantly differential interaction sites. *E*, the average |log_2_FC| of SDISs of the myogenic marker genes. *F*, a density plot showing the |Log_2_FC| distribution of up- and down-regulated SDISs for *Myod1* and *Mef2a*. Vertical red and blue dashed lines indicate the mean value of Log_2_FC for upregulated and downregulated SDISs, respectively. *G*, a density plot showing the |Log_2_FC| distribution of *cis*- and *trans*-SDISs for *Myod1* and *Mef2a*. Vertical *red* and *blue* dashed lines indicate the mean value of |Log_2_FC| for *trans*- and *cis*-SDISs, respectively. MB, myoblast; MT, myotube; SDIS, significant differential interaction site.

To investigate the chromatin interaction alterations of the myogenic marker genes during differentiation, we performed a cluster analysis, which revealed a clear split between C2C12-MBs and C2C12-MTs for the *Myod1*, *Myog*, *Mef2d*, *Myh2*, and *Myh3* genes but a weak division for the *Mef2a*, *Mef2b*, and *Mymk* genes (Figs. 2*A* and S6*A*). Principal component analysis suggested an interaction discrepancy of the myogenic marker genes in C2C12-MBs and C2C12-MTs. (Figs. 2*B* and S6*B*). In addition, we compared the interaction profiles of the myogenic marker genes within ±500 kb of the viewpoint in C2C12-MBs and C2C12-MTs. The interaction sites of the myogenic marker genes were changed (Fig. S7). Together, these results suggested that chromatin interactions of the myogenic marker genes differed to some extent between C2C12-MBs and C2C12-MTs.

To clarify the detailed interaction changes during differentiation, we identified significant differential interaction sites (SDISs) between C2C12-MBs and C2C12-MTs using DESeq2 analysis with a *q*-value ≤ 0.01. Many SDISs were observed (Figs. 2, *C* and *D* and S8*A*), with the proportion of SDISs *versus* total interaction sites close to or greater than 40% for genes (*Myod1*, *Myog*, *Mef2a*, *Mef2d*, *Myh2*, and *Myh3*). Of note, the *Myh2* gene had the most SDISs, with more than 70% of interaction sites significantly changed. In comparison, 27.54% and 33.28% of interaction sites were changed for the *Mef2b* and *Mymk* genes, which had the least SDISs. In addition, the mean |Log_2_FC| of SDISs ranged from 7.48 to 12.42 for each gene (Fig. 2*E*), indicating a marked alteration in the interaction frequency of SDISs during differentiation. For example, *Myod1* had the largest average |Log_2_FC| (12.42) of SDISs. We also analyzed the mean fold changes for SDISs within ±500 kb of myogenic marker genes and found that the mean |Log_2_FC| of SDISs ranged from 5.49 to 10.73 (Fig. S8*B*). These results indicated a marked change in the interaction profiles of myogenic marker genes during myogenesis. Next, we examined the fold change distribution of the upregulated and downregulated SDISs. The results showed that the |Log_2_FC| values of upregulated and downregulated SDISs had different distributions (Figs. 2*F* and S9*A*). Except for *Myog*, most upregulated SDISs of other myogenic genes had larger fold changes than those of most downregulated SDISs, such as *Myod1* and *Mef2a* (Figs. 2*F* and S9*A*). We also examined the fold change of *cis*- and *trans*-SDISs and created density plot of the fold change distribution. We found that most *trans*-SDISs of seven myogenic genes had larger fold change than most *cis*-SDISs, such as *Myod1* and *Mef2b* (Figs. 2*G* and S9*B*). However, most *cis*-SDISs of *Myog* had higher fold change than most *trans*-SDISs (Fig. S9*B*). This suggests that the *trans*-interactions of most myogenic marker genes have a stronger change in interaction frequency than the *cis*-interactions between C2C12-MBs and C2C12-MTs. These results indicate that the interaction sites and interaction intensity of myogenic marker genes changed markedly during differentiation.

### SIS analysis reveals an enhanced coregulatory network and open chromatin state during myogenic differentiation

**Figure 3:**
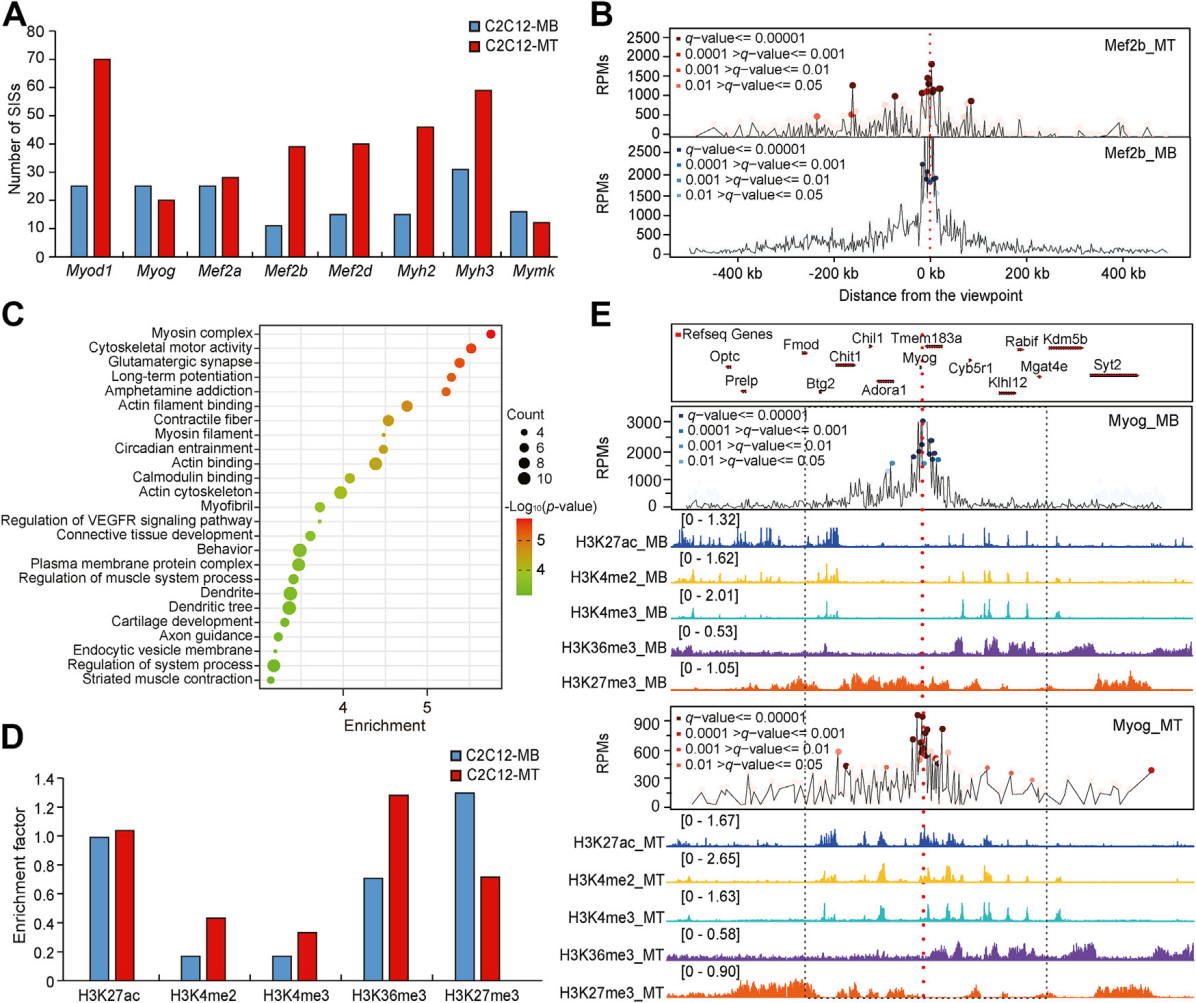
**The coregulated network and open chromatin state of SISs during myogenic differentiation.***A*, the SISs numbers of the myogenic marker genes in C2C12-MBs and C2C12-MTs. *B*, interactions of the *Mef2b* within ± 500kb of the viewpoint. The *dotted red line* represents the viewpoint. *Circles* represent interactions. The deeper color indicates a higher interaction frequency. The *y*-axis indicates reads per million (RPM). *C*, GO and KEGG pathway enrichment analyses of the interacting genes in C2C12-MTs. The top 25 terms are shown. Dot size represents the number of genes, and the color bar represents the -Log_10_ (*p*-value). *D*, enrichment analysis of histone marks of the SISs. Bar plots showing the enrichment factor values for histone modifications of the SISs in C2C12-MBs and C2C12-MTs. *E*, histone modification of interaction sites of the *Myog* locus in C2C12-MBs and C2C12-MTs. *Red circles* represent interactions in C2C12-MTs, and *blue circles* represent interactions in C2C12-MBs. The *dotted red line* represents the viewpoint of the *Myog*. ChIP-seq profiles for H3K27ac (*blue*), H3K4me2 (*yellow*), H3K4me3 (*cyan*), H3K36me3 (*purple*), and H3K27me3 (*orange*) at the *Myog* locus in C2C12-MBs and C2C12-MTs. ChIP-seq, chromatin immunoprecipitation sequencing; MB, myoblast; MT, myotube; SIS, significant interaction site.

The high confidence of the SISs suggests that they are potential candidate regulatory elements that regulate gene expression (21, 30, 31). Therefore, we used r3Cseq to detect SISs (*q*-value ≤ 0.05) of the myogenic marker genes in C2C12-MBs and C2C12-MTs. Overall, 477 SISs were detected, with 163 and 314 in C2C12-MBs and C2C12-MTs, respectively (Table S9). Of note, six of eight genes had more SISs in C2C12-MTs than in C2C12-MBs (Fig. 3*A*). For example, the *Mef2b* gene had more SISs within ±500 kb around the viewpoint in C2C12-MTs (n = 15) than C2C12-MBs (n = 11) (Fig. 3*B*). We also identified peaks using PeakC (35) and used on monotonic regression to confirm this result. When the wSize set at 5, the peak number of each myogenic marker gene in C2C12-MTs was higher than in C2C12-MBs (Fig. S10*A*). Although the numbers of SISs and peaks identified by the two software packages were different, both methods revealed more SISs or peaks in C2C12-MTs, which had the higher expression levels of the myogenic marker genes. This result is consistent with a previous study (32) reporting increased gene expression was positively correlated with the number of promoter interactions. A detailed comparison indicated that only *Myog* and *Mymk* share about 40% of SISs between C2C12-MBs and C2C12-MTs; the other genes only share about 10% of SISs (Fig. S10*B*), suggesting dynamic chromatin interactions during differentiation. In summary, these results indicate that the interaction profile of the myogenic marker genes changes during C2C12-MB differentiation.

Interactions between genes involved in related biological pathways have been detected in various biological processes, suggesting spatial networks of coregulated genes (32). To examine potential differences in spatial networks during differentiation, interacting genes that contained at least one SIS within intragenic regions were identified. We identified 15 interacting genes in C2C12-MBs and 84 interacting genes in C2C12-MTs, suggesting an increased network during myogenic differentiation (Table 1). *Myh* genes (*Myh1*, *Myh2*, *Myh3*, *Myh4*, *Myh8*, and *Myh13*) are located adjacently across the genome and are coexpressed to promote myogenesis (33, 34). In this study, we observed a significant interaction between *Myh2* and *Myh1* in C2C12-MBs. In comparison, *Myh2* interacted significantly with the *Myh1*, *Myh3*, *Myh8*, and *Myh13* genes in C2C12-MTs, indicating the increased connection of fast *Myh* (*fMyh*) genes during differentiation. These results demonstrated the coregulated expression of the *fMyh* genes and their potential coordinated roles in myogenic differentiation. To further investigate the biological function of the interacting genes, functional enrichment analysis was performed. The interacting genes in C2C12-MTs that showed significant enrichment were related to muscle development and function, including the terms “myosin complex” (five genes, *p* < 1.76 × 10^−6^), “cytoskeletal motor activity” (six genes, *p* < 3.01 × 10^−6^), “glutamatergic synapse” (six genes, *p* < 4.14 × 10^−6^), “long-term potentiation (five genes, *p* < 5.18 × 10^−6^)”, “actin filament binding” (seven genes, *p* < 1.74 × 10^−5^), “contractile fiber” (seven genes, *p* < 2.90 × 10^−5^), and “regulation of system process” (nine genes, *p* < 6.67 × 10^−4^) (Fig. 3*C* and Table S10). In C2C12-MBs, the interacting genes were not enriched for any term.

**Table 1:** The interacting genes of the myogenic marker genes in C2C12-MTs and C2C12-MBs

Gene	The interacting genes in C2C12-MTs	The interacting genes in C2C12-MBs	Shared the interacting genes
*Myod1*	*Abcc8*, *Ablim2*, *Gnao1*, *Gria1*, *Grin2d*, *Hif1a*, *Kcnc1*, *Myo10*, *Nup133*, *Otog*, *Parp14*, *Pitx1*, *Pkib*, *Prkch*, *Sipa1l3*, *Usp48*, *Zfp507*	*Otog*, *Sergef*	*Otog*
*Myog*	*Chit1*, *Klhdc8a*, *Ppfia4*	*Adora1*, *Ppfia4*, *Slc41a2*	*Ppfia4*
*Mef2a*	*Dnah1*, *Lrrc28*, *Mbd2*, *Sema5a*, *Sema6d*, *Spink5*, *Tanc2*, *Ttc23*, *Zbtb16*	*Lrrc28*	*Lrrc28*
*Mef2b*	*Arhgef3*, *Cald1*, *Cdkal1*, *Cnpy2*, *Fgf9*, *Gatad2a*, *Gm20604*, *Gypc*, *Otulinl*, *Rfc1*, *Yjefn3*	*Tmem161a*, *Borcs8*	
*Mef2d*	*Atl1*, *Cacna1c*, *Dlg1*, *Fnbp1l*, *Galnt2*, *Iqgap3*, *Rorc*, *Ttn*, *Vmn2r87*, *Wwox*	*Iqgap3*	*Iqgap3*
*Myh2*	*Abca8a*, *Anxa3*, *Cep112*, *Cmip*, *Col23a1*, *Klhl1*, *Myh1*, *Myh3*, *Myh8*, *Myh13*, *Rcvrn*, *Svop*, *Tmem132d*, *Tpgs2*, *Zfp804b*	*Myh1*	*Myh1*
*Myh3*	*Acaca*, *Dnah9*, *Epn2*, *Filip1l*, *Myh13*, *Ppp3r2*, *Rpe*, *Rsu1*, *Sco1*, *Stx8*, *Tbc1d5*, *Tmem220*, *Ttc1*, *Ubtd2*, *Usp34*, *Usp43*	*Adprm*, *Sco1*, *Tmem220*	*Sco1*, *Tmem220*
*Mymk*	*Adamtsl2*, *Fam155a*, *Prkca*, *Vav2*	*Adamtsl2*, *Sardh*	*Adamtsl2*

Next, we integrated our 4C-seq data with published epigenome datasets, including five histone modifications (H3K27ac, H3K4me2, H3K4me3, H3K36me3, and H3K27me3) to calculate enrichment factors for each histone mark in the SISs to assess the regulatory potential of the SISs (32, 35). Given that most SISs are located on the *cis*-chromosome, we mainly focused on the chromatin state of the SISs on the *cis*-chromosome. We found that histone hallmarks of open chromatin, such as H3K27ac, H3K4me2, H3K4me3, and H3K36me3, had high enrichment levels in C2C12-MTs compared with the enrichment in C2C12-MBs (Fig. 3*D*, Table S11, and Fig. S11). In contrast, the repressive histone mark H3K27me3 had high levels of enrichment in C2C12-MBs. For example, *cis*-SISs of the *Myog* gene showed increased enrichment of the H3K27ac, H3K4me2, and H3K4me3 modifications but decreased enrichment of H3K27me3 in C2C12-MTs (Fig. 3*E*). These results indicated dynamic histone modifications of the SISs occur during differentiation and that the chromatin state of the SISs in C2C12-MTs tends to be open.

### Identification and characterization analysis of active enhancers of myogenic marker genes

**Figure 4:**
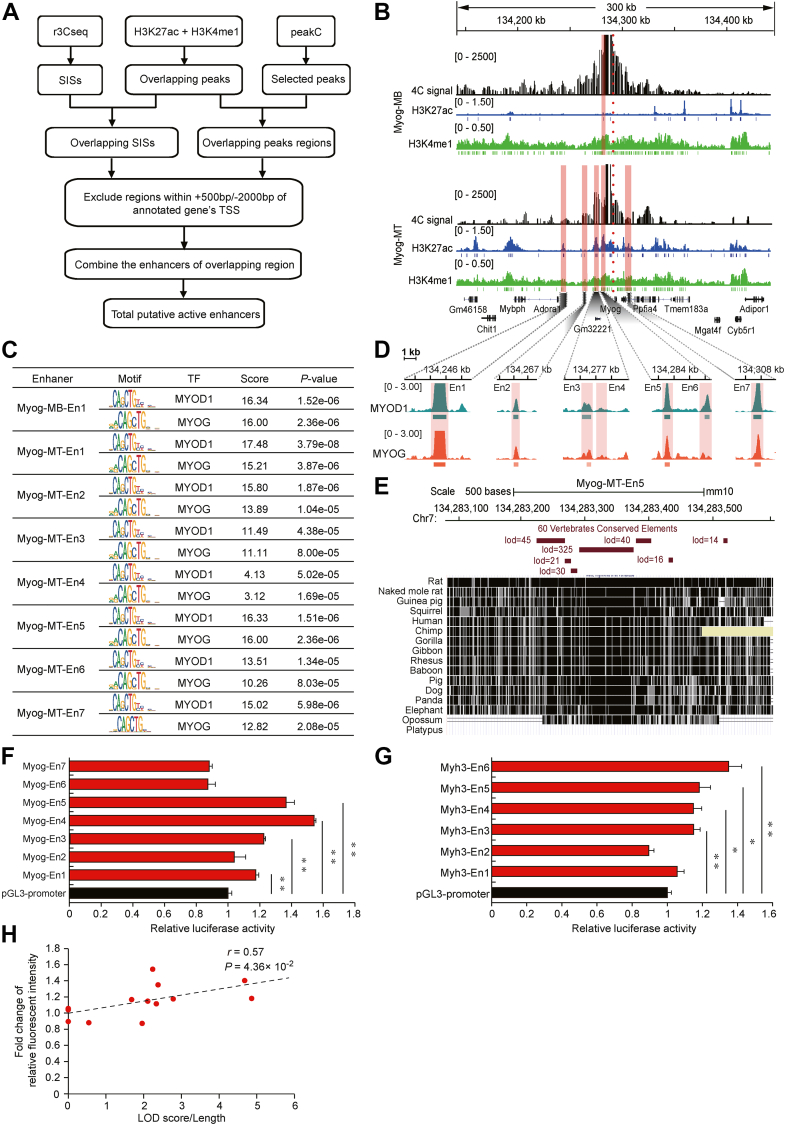
**Identification and activity evaluation of enhancers.***A*, scheme of putative active enhancer identification strategy. *B*, manual selection of putative active enhancers of *Myod1* in C2C12-MBs and C2C12-MTs. View of a genomic region around *Myod1* integrating ChIP-seq profiles for H3K27ac (*blue*) and H3K4me1 (*green*) with 4C interaction profile (*black*). Upper panel: 4C interaction profile of *Myog* in C2C12-MBs. Lower panel: 4C interaction profile of *Myog* in C2C12-MTs. The *blue* and *green* vertical lines below the ChIP-seq profiles indicate the peak. The *red* dotted line represents the viewpoint of *Myod1*. The *orange* column represents the putative active enhancers. *C*, MYOD1 and MYOG TFs motif enrichment analysis of putative active enhancer regions of *Myog*. *D*, zoom in view of MYOD1 and MYOG ChIP-seq signals. Shaded regions showing seven putative active enhancers (Myog-MT-En1-En7). *E*, conservation analysis of putative active enhancers of Myog-MT-En5. UCSC genome browser (http://genome-asia.ucsc.edu/; GRCm38/mm10) shows conserved regions (*black boxes*) of putative active enhancers in selected species. Horizontal *red* bars indicate conservative elements in 60 vertebrates. Elements conservation is measured as the LOD score of phastCons elements. *F* and *G*, dual-luciferase reporter assay assessing the enhancer activity of *Myog* and *Myh3*. Luciferase reporter assays was performed in H293T cells. The pGL3-promoter was used as control. Luciferase signals were normalized to renilla signals. Data are represented as mean ± SD of three independent experiments, and *p* values are calculated using Student’s *t* test (∗*p* < 0.05, ∗∗*p* < 0.01). *H*, correlation analysis between LOD score/length and relative fluorescence intensity of enhancers of *Myog* and *Myh3*. The *x*-axis indicates LOD score/length values, and the *y*-axis indicates relative fluorescence intensity values. The *black* dotted line represents the correlation line. The Pearson correlation coefficients and significances are indicated at the right upper corner of the plot. 4C, circularized chromosome conformation capture; ChIP-seq, chromatin immunoprecipitation sequencing; MB, myoblast; MT, myotube; TF, transcription factor.

We used two 4C-seq software programs to identify putative active enhancers, based on a nonoverlapping window and number of fragment ends. The two software programs have advantages in identifying the precision and recall of interaction regions (36). After identifying putative active enhancers (Fig. 4*A*), we identified 41 and 25 putative active enhancers in C2C12-MTs and C2C12-MBs, respectively (Table 2). As expected, more putative active enhancers were observed in C2C12-MTs, consistent with a previous study reporting a positive correlation between gene expression level and number of interacting enhancers (32). For example, we detected seven putative active enhancers of *Myog* in C2C12-MTs, which showed apparent histone modifications of H3K27ac and H3K4me1. In comparison, only one putative active enhancer was detected in C2C12-MBs (Fig. 4*B*).

**Table 2:** The number of putative active enhancers of myogenic marker genes in C2C12-MTs and C2C12-MBs

Gene	C2C12-MT		C2C12-MB	
	Number of enhancers	Containing conserved elements	Number of enhancers	Containing conserved elements
*Myod1*	8	8	7	7
*Myog*	7	6	1	1
*Mef2a*	8	8	10	10
*Mef2b*	2	1	0	0
*Mef2d*	3	3	2	2
*Myh2*	1	1	1	0
*Myh3*	6	4	3	2
*Mymk*	6	4	1	1
Total	41	35	25	23

The enhancer serves as a platform to supply binding sites of TFs to regulate gene expression (37, 38). Furthermore, genetic variants within enhancers that disrupt TF-binding motifs may lead to misregulated gene expression. It is therefore important to investigate TF binding in enhancer regions. We analyzed which myogenic TFs (MYOD1, MYOG, MYF6, and MEF2A-D) might bind to the enhancers of myogenic marker genes by performing motif enrichment analysis using AnimalTFDB3.0 (39) and JASPAR (40). All putative active enhancers were predicted to contain at least three types of myogenic TF-binding sites (Table S12). For enhancers of *Myod1*, *Myh2*, *Myh3*, and *Mymk*, we observed that the binding sites of MYOD1 TF were significantly enriched (*p* < 0.05). MYOD1 is a transcriptional activator that activates its own transcription and promotes the transcription of muscle-specific target genes during muscle differentiation (5, 41, 42). Of note, the motifs of MYOD1 and MYOG TFs were significantly enriched in most enhancer sequences of *Myog* (Fig. 4*C*), suggesting the TFs might cobind to functional enhancers of *Myog* to promote the transcription of *Myog*. Next, we downloaded the chromatin immunoprecipitation sequencing (ChIP-seq) data of MYOD1 and MYOG to determine whether MYOD1 and MYOG TFs occupied the enhancer of *Myog*. The ChIP-seq results confirmed that the enhancer regions of *Myog* were occupied by MYOD1 and MYOG TFs (Fig. 4*D*), which is consistent with the predicted results of TF-binding sites.

Previous studies have shown that enhancers are usually evolutionarily conserved, which allows them to be used to identify functional elements within genomes (43, 44, 45, 46, 47, 48, 49). To evaluate the conservation of the putative active enhancers, we used the UCSC genome browser (http://genome-asia.ucsc.edu/) to perform conserved element analysis in 60 vertebrates. Overall, 58 out of 66 (87.88%) putative active enhancers contained at least one conserved element (Table 2, Fig. S12, and Table S13), including Myog-MT-En5 (Fig. 4*E*). These results suggested the high confidence of the putative active enhancers. To validate the transcriptional activity of putative active enhancers, we performed luciferase reporter assays for 13 candidate enhancers of two genes (*Myog*: a transcription factor that controls myoblast differentiation; *Myh3*: a contractile protein important for muscle contraction), which are important for the differentiation of myoblasts. Eight enhancers had activity that increased gene expression. For enhancers of *Myog*, we found that Myog-En1, Myog-En3, Myog-En4, and Myog-En5 had a significant increase in luciferase activity compared with the pGL3-promoter vector (Fig. 4*F*), and Myog-En4 had the strongest activity (∼1.5-fold higher). *Myh3* enhancers other than Myh3-En1 and Myh3-En2 significantly increased the luciferase activity (Fig. 4*G*). To test whether the enhancement was associated with its conservation, we conducted Pearson correlation coefficient analysis between Lod score/length and the fold change of relative fluorescent intensity across 19 putative active enhancers. There was a significant positive Pearson correlation coefficient (*r* = 0.57, *p* < 0.05) (Fig. 4*H*), suggesting a weak relationship, but a significant correlation, between the activity of enhancers and their conservation. In summary, we comprehensively analyzed putative active enhancers and evaluated their transcriptional activity.

### The repression of *Myog* enhancers decreases *Myog* expression and inhibits myogenic differentiation

**Figure 5:**
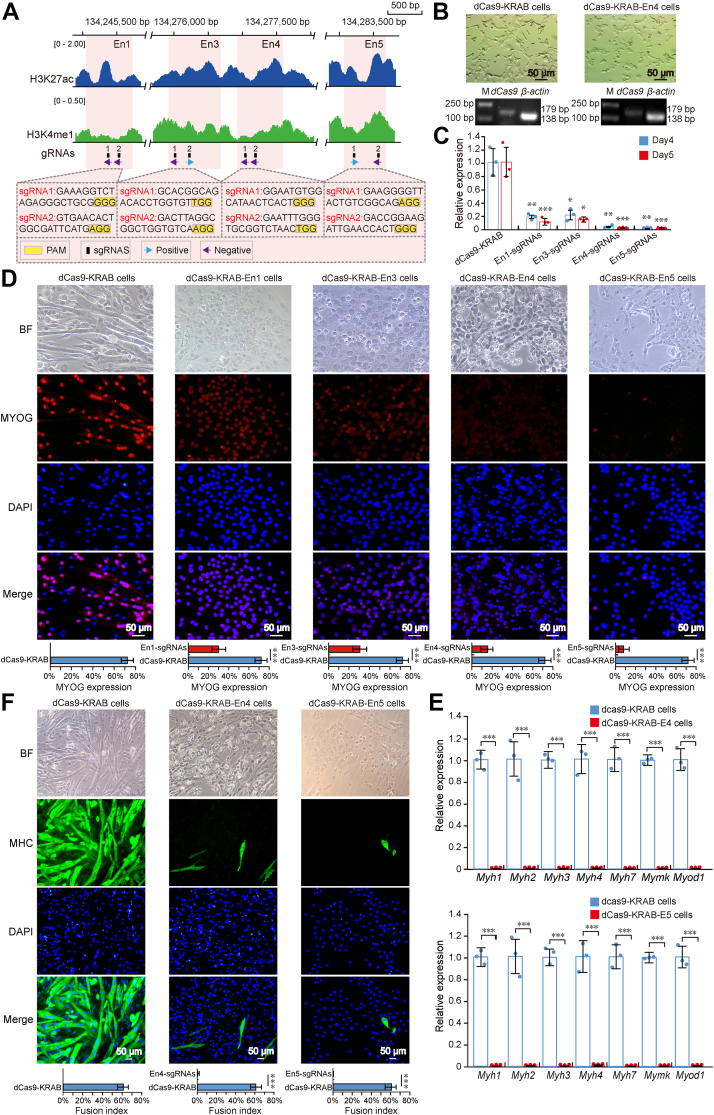
**Enhancers are required for *Myog* expression myoblast differentiation.***A*, ChIP-seq profiles of H3K27ac and H3K4me1 are shown at Myog-En1, Myog-En3, Myog-En4, and Myog-En5 regions in C2C12-MTs. Enhancers regions are shown as *orange* rectangle. sgRNA target locations are indicated in *black* with corresponding black numbers, and the direction of the arrow indicates the gRNA targets the forward or reverse DNA strand. *B*, proliferating C2C12 cells expressing dCas9-KRAB or dCas9-KRAB-En5-sgRNAs system after 10 days of puromycin selection and qRT-PCR analysis of *dCas9* and *β-actin* genes. cDNA samples were obtained from dCas9-KRAB cells and dCas9-KRAB-En4 cells, respectively. PCR products were run on a 1.2% agarose gel. *β-actin* served as an internal control. Lane M: 2 kb DNA marker. *C*, relative expression of *Myog* transcripts measured by qRT-PCR in C2C12 cells transduced with dCas9-KRAB, dCas9-KRAB-En1-sgRNAs, dCas9-KRAB-En3-sgRNAs, dCas9-KRAB-En4-sgRNAs, or dCas9-KRAB-En5-sgRNAs and induced to differentiate for 4 days or 5 days. Relative expression levels of genes were normalized to *β-actin*. Data are presented as mean ± SD (n = 3). ∗*p* < 0.05, ∗∗*p* < 0.01, ∗∗∗*p* < 0.005. *D*, immunofluorescence detection of Myogenin (MYOG) in C2C12 cells transduced with dCas9-KRAB, dCas9-KRAB-En1-sgRNAs, dCas9-KRAB-En3-sgRNAs, dCas9-KRAB-En4-sgRNAs, or dCas9-KRAB-En5-sgRNAs and differentiated for 5 days, MyoG (*red*) and DAPI (*blue*). Quantification of Myogenin-positive nuclei in dCas9-KRAB cells and dCas9-KRAB-sgRNAs cells. Data are presented as mean ± SD (10 randomly chosen microscopic fields). ∗∗∗*p* < 0.005. *E*, qRT-PCR for myogenic genes in dCas9-KRAB cells, dCas9-KRAB-En4 cells, and dCas9-KRAB-En5 cells after 5 days of differentiation. Relative expression levels of genes were normalized to *β-actin*. Data are presented as mean ± SD (n = 3). ∗∗∗*p* < 0.005. *F*, immunofluorescence detection of myosin heavy chain (MHC) in C2C12 cells transduced with dCas9-KRAB, dCas9-KRAB-En4-sgRNAs or dCas9-KRAB-En5-sgRNAs and differentiated for 5 days, MHC (*green*) and DAPI (*blue*). The fusion index (percentage of MTs containing more than three nuclei) in dCas9-KRAB cells, dCas9-KRAB-En4 cells, and dCas9-KRAB-En5 cells. Data are presented as mean ± SD (10 randomly chosen microscopic fields). ∗∗∗*p* < 0.005. ChIP-seq, chromatin immunoprecipitation sequencing; DAPI, 4′,6-diamidino-2-phenylindole; MT, myotube; qRT-PCR, quantitative RT-PCR; sgRNA, single guide RNA.

Understanding the complex mechanism involved in the transcriptional regulation of myogenic genes remains a major challenge. Classical luciferase reporter assay studies have already enabled the discovery of active enhancers; however, the influence of genomic context is still largely unknown. To test the transcription regulating potential of active enhancers for the target gene, we selected the active enhancers of the transcription factor *Myog*, which is critical for myoblast differentiation in functional studies. First, we visualized published Hi-C data (http://3dgenome.fsm.northwestern.edu/index.html) and analyzed published SMC3 (a subunit of the cohesin complex) and CTCF ChIP-seq data from mouse muscle tissues or cells. These results showed that these Myog-Ens and *Myog* promoter are located in an interaction domain, and they are present in the cohesin- and CTCF-enriched DNA region with convergent CTCF motif orientation (Fig. S13), suggesting that cohesin-mediated loop might be responsible for this interaction (50, 51). Then, we separately targeted four active enhancers of *Myog* using catalytically inactive Cas9 fused to a KRAB domain system, with two single guide RNAs (sgRNAs) for each enhancer (Fig. 5*A*). We transduced C2C12 cells with lentiviruses expressing dCas9-KRAB or dCas9-KRAB-sgRNAs (Figs. 5*B* and S14). The repression of Myog-En1, En3, En4, and En5 significantly decreased *Myog* expression compared with cells expressing dCas9-KRAB on days 4 and 5 of differentiation (Fig. 5*C*). Of note, *Myog* expression was significantly reduced by dCas9-KRAB-En4-gRNAs (0.027-fold, *p* < 0.01) and dCas9-KRAB-En5-gRNAs (0.02-fold, *p* < 0.005) on day 5 of differentiation. The immunofluorescence analysis of Myogenin (MYOG) was performed using C2C12 cells expressing dCas9-KRAB or dCas9-KRAB-sgRNAs. The repression of Myog-En1, En3, En4, and En5 reduced the proportion of Myogenin-positive cells and fluorescence intensity compared with dCas9-KRAB at day 5 of differentiation, indicating the repression of enhancers impaired MYOG expression (Fig. 5*D*). For C2C12 cells expressing dCas9-KRAB-En4-sgRNAs or dCas9-KRAB-En5-sgRNAs, the proportion of Myogenin-positive cells was 23.81% and 7.86%, respectively. In addition, qRT-PCR analysis showed that myogenic genes including *Myod1*, *Myh1*, *Myh2*, *Myh3*, *Myh4*, *Myh7*, and *Mymk* of dCas9-KRAB-En4 cells and dCas9-KRAB-En5 cells were significantly downregulated compared with dCas9-KRAB cells (Fig. 5*E*). To investigate whether the repression of Myog-En4 and En5 affected myoblast differentiation, we performed immunofluorescence staining of MHC on dCas9-KRAB cells and dCas9-KRAB-sgRNAs cells. We observed few MHC-positive cells on day 5 of differentiation, indicating the repression of Myog-En4 and En5 markedly attenuated the ability of C2C12 to differentiate and form multinucleated MTs (Fig. 5*F*).

**Figure 6:**
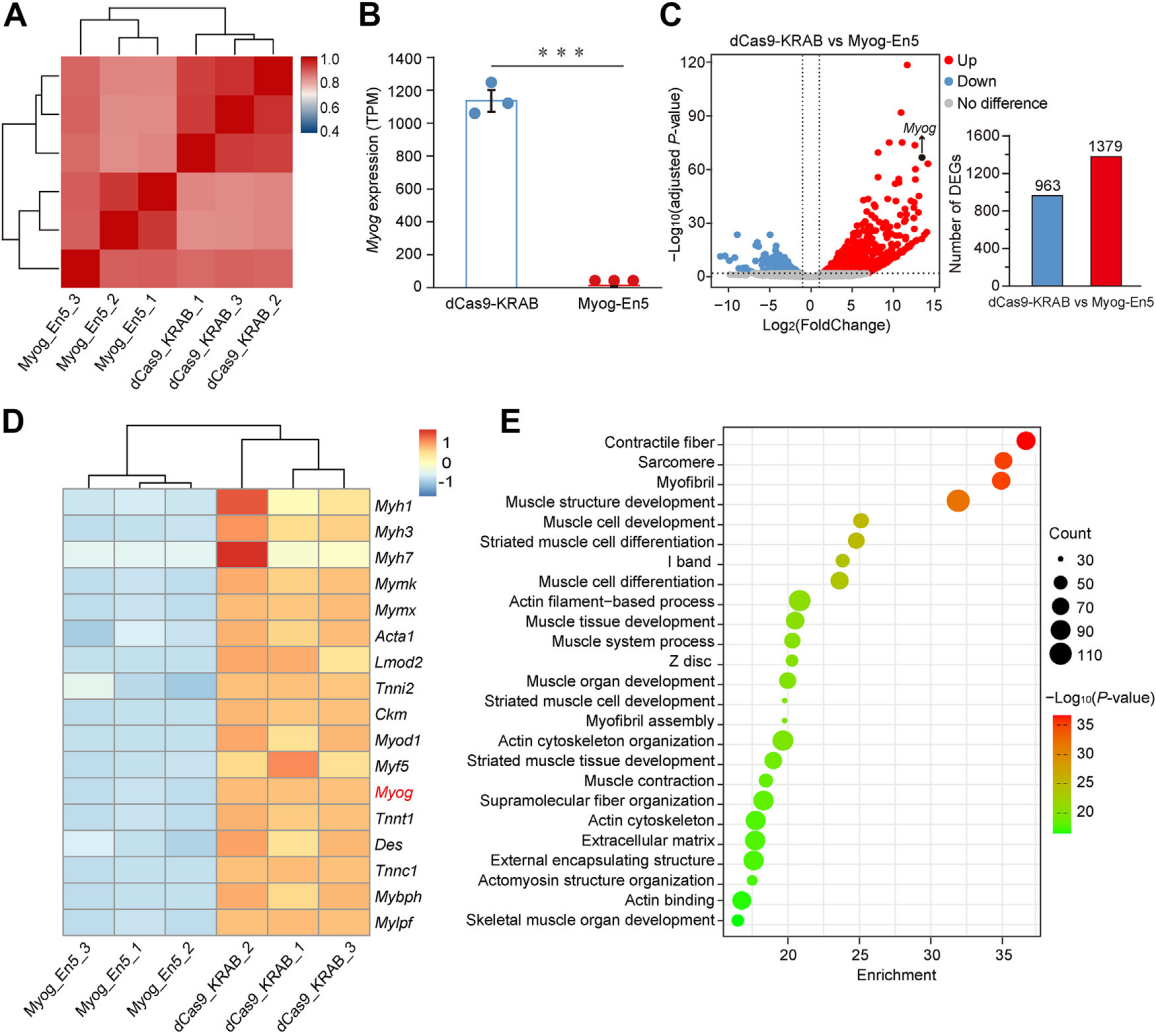
**Effects of Myog-En5 repression on the transcriptome of myogenic differentiation.***A*, heatmap shows hierarchical clustering of Spearman correlation scores between samples based on RNA-Seq profiles. Color represents the Spearman correlation coefficient, and correlation scores are plotted from 0.4 (*blue*) to 1 (*blue*). *B*, the expression levels of *Myog* in dCas9-KRAB-En5 cells and dCas9-KRAB cells. The bars show the mean and SD of TPM values (n = 3). ∗∗∗*p* < 0.005. *C*, volcano plot of differentially expressed genes between dCas9-KRAB cells *versus* dCas9-KRAB-En5 cells. *Blue* and *red* dots indicate genes upregulated in dCas9-KRAB-En5 cells and upregulated in dCas9-KRAB cells, respectively (adjusted *p*-value ≤ 0.01 and absolute Log_2_ fold-change ≥ 1). *Gray dots* represent genes with no significant difference, and *black dot* indicates *Myog* gene. *D*, heatmap representation of the expression levels (z-scores) of myogenic genes in dCas9-KRAB-En5 cells and dCas9-KRAB cells. Genes and samples were subjected to hierarchical clustering. *E*, functional enrichment analyses of downregulated DEGs in dCas9-KRAB-En5 cells. The top 25 terms are shown. Dot size represents the number of genes, and the color bar represents the -Log_10_(*p*-value). DEG, differentially expressed gene; TPM, transcripts per million.

Next, to examine the effect of Myog-En5 repression on differentiation in a transcriptome-wide manner, we conducted RNA-Seq analysis for dCas9-KRAB cells and dCas9-KRAB-En5 cells. Hierarchical clustering between samples revealed that samples between replicates within the same group have better correlations than samples between different groups (Fig. 6*A*). RNA-Seq analysis revealed *Myog* expression was reduced significantly (*p* < 0.01) in dCas9-KRAB-En5 cells than in dCas9-KRAB cells (Fig. 6*B*), which is in line with the qRT-PCR results (Fig. 5*C*). Differential expression analysis indicated that 2342 genes were differentially expressed, including 1379 upregulated and 963 downregulated genes in dCas9-KRAB cells (Fig. 6*C*). Known MYOG targets (*Myh1*, *Myh3*, *Myh7*, *Mymk*, *Mymx*, *Acta1*, *Lmod2*, *Tnni2*, and *Ckm*) (6, 52), the myogenic regulatory factors (*Myf5*, *Myod1*, and *Myog*), and late myogenic differentiation genes (*Tnnt1*, *Des*, *Tnnc1*, *Mybph*, and *Mylpf*) (4, 53, 54) were significantly downregulated (*p* < 0.01) in dCas9-KRAB-En5 cells (Fig. 6*D*). Gene Ontology (GO) enrichment and the Kyoto Encyclopedia of Genes and Genomes (KEGG) pathway analysis of downregulated differentially expressed genes (DEGs) in dCas9-KRAB-En5 cells revealed significant enrichment for terms implicated in muscle structure and differentiation, such as contractile fiber, sarcomere, myofibril, muscle structure development, and muscle cell differentiation. (Fig. 6*E* and Table S14). In addition, Gene Set Enrichment Analysis (GSEA) showed that muscle cell development, muscle contraction, and structural constituent of muscle were enriched in dCas9-KRAB cells (Fig. S15). These findings indicate that Myog-En5 repression alters the transcriptome for MB differentiation and inhibits myogenic differentiation, demonstrating that Myog-En5 is critical for regulation of *Myog* transcription and myogenesis.

## Discussion

The formation of skeletal muscles involves 3D chromatin interactions and epigenetic modifications, which ultimately lead to alterations in myogenic gene expression (14, 55, 56, 57). The expression of myogenic genes is essential for myogenesis, and their abnormal expression directly affects muscle growth and development (58, 59, 60, 61). In this study, we analyzed the chromatin interactomes of eight myogenic marker genes and studied the alterations of their chromatin interactions during differentiation. We identified putative active enhancers of myogenic marker genes and performed sequence characterization analysis and transcriptional activity. Finally, we studied the role of *Myog* enhancers in the regulation of *Myog* expression and myogenic differentiation by epigenome editing and RNA-seq.

4C-seq is a powerful technique for studying the 3D chromatin interactions of a locus of interest at the resolution of several kilobases (62, 63, 64). Compared with Hi-C and PCHi-C, 4C-seq enables the generation of a high-resolution interaction map with a relatively low amount of sequencing data (12). To date, 4C-seq has been widely used to study the chromatin interaction of gene promoters (29), enhancers (65), lncRNA (66), and SNP sites (67). We utilized 4C-seq to study the chromatin interactions of eight myogenic marker genes during C2C12 myogenic differentiation. The correlation of chromatin interactions of each gene between replicates of C2C12-MBs was lower than that of C2C12-MTs, indicating that differentiated C2C12 have heterogeneity compared with the highly homogeneous C2C12-MBs before differentiation. We speculated that this was because the nuclei were heterogeneous at different differentiation stages (25), resulting in a more diverse interaction of the myogenic marker genes in C2C12-MTs. Similarly, this heterogeneity of cell differentiation was also reported during the adipogenic differentiation of 3T3-L1 (68). C2C12 can express a variety of MyHC isoform proteins after differentiation *in vitro*, leading to the production of nuclei of different MT types (26). This may increase the diversity of *Myh* gene chromatin interactions. We found that the *Mef2a*, *Mef2b*, and *Mymk* genes did not cluster together in C2C12-MTs, suggesting that they might have more diverse chromatin interaction patterns after differentiation.

Gene expression and cell differentiation are often accompanied by changes in chromatin conformation (69, 70, 71, 72, 73). Our results showed that the number of chromatin interactions and the proportion of *cis*- and *trans*-chromatin interactions for myogenic marker genes are markedly changed after differentiation and that the proportion of *trans*-interactions is increased in C2C12-MTs, especially for *Myod1* and *Myog*. Considering that these myogenic marker genes are highly active in C2C12-MTs and that the regions in which they are located show higher chromatin activity, these active regions may interact with active regions of other chromosomes with a higher interaction frequency than the average contact frequency in the genome. Furthermore, Tsai *et al.* reported that ^DRR^eRNA, a noncoding RNA transcribed from the enhancer regions of *Myod1*, affects the expression of *Myog* in *trans* and found that the distance separating the *Myod1* (chr7) and *Myog* (chr1) loci is reduced during C2C12-MB differentiation, suggesting that *Myod1* and *Myog* have increased *trans*-interactions after muscle cell differentiation (74). From this, we speculate that these *trans*-interactions between active chromatin regions may be able to regulate gene expression through other modes of action, such as eRNAs. Differential analysis characterized changes in chromatin interactions of the myogenic marker genes before and after differentiation. Upregulated SDISs of most myogenic genes have greater changes in interaction frequency than downregulated SDISs after differentiation and *trans*-SDISs of most myogenic genes have greater changes in interaction frequency than *cis*-SDISs after differentiation, indicating that the chromatin interaction of myogenic marker genes markedly changed not only in the interaction number but also in the interaction frequency. Although previous studies also reported changes in chromatin interactions during myogenesis (13, 14, 15), these studies focused on large-scale chromatin structures such as the chromosome territory, compartments A/B, topologically associating domain, and sub-topologically associating domain, and did not conduct in-depth studies on the high-resolution chromatin interaction changes of individual genes. Our study focused on eight myogenic marker genes and analyzed changes in chromatin interactions during differentiation in detail by mapping their high-resolution interaction profiles. In addition to chromatin interactions, gene expression is also affected by the chromatin state (75, 76, 77). The SISs of highly expressed genes in C2C12 MTs were enriched for different types of active histone marks, and the SISs of low-expressed genes in C2C12 MBs were more enriched for repressive histone marks. This is in accord with previous studies reporting that highly expressed genes interacted with regions enriched for active histone marks and that weakly expressed and silent genes interacted with regions depleted of active histone marks (27, 32). This implies that the expression of myogenic marker genes is related to the chromatin state of their SISs and that SISs of highly expressed genes tend to interact more with open chromatin.

In the human genome, 98% of DNA sequences are noncoding regions that were previously disregarded as “junk” DNA (78, 79). However, noncoding regions contain a variety of *cis*-regulatory regions, such as enhancers, which precisely control the expression of genes. Thus, identifying the active enhancer in the genome is critical for understanding gene regulation and assessing the impact of genetic variation on phenotype. We used two 4C-seq software combined with ChIP-seq of H3K27ac and H4K4me1 to identify the putative active enhancers of eight myogenic marker genes. The interaction sites identified by r3Cseq were a continuous nonoverlapping 2 kb window. Considering that an enhancer might be located in two adjacent windows, it is difficult to use r3Cseq to determine the exact location of the enhancer. Therefore, we used the visual inspection method based on PeakC contact profiles as a supplement to identify the enhancer further. The two methods have their advantages and both are widely used to identify enhancers of genes (36). For example, Pan *et al.* utilized r3Cseq to identify significant interactions of the *HNF4A* gene and detected three distal enhancers of the *HNF4A* gene in combination with ChIP-seq H3K27ac in a study of gastrointestinal adenocarcinomas (31). Maqbool *et al.* mapped 4C profiles of sliding windows of 21 fragments and ChIP-seq profiles of H3K27ac and Pol II at *Lef1*, *S1pr1*, *mir181*, and *Nfatc1* loci, visually inspected the profiles, manually selected their putative enhancers, and found moderate dynamics of enhancer–promoter interactions during T cell differentiation (80). In keeping with these two methods, we identified the putative enhancers of eight genes in C2C12-MBs and C2C12-MTs. Counting the number of enhancers, we found that the number of putative active enhancers in C2C12-MTs was higher than that in C2C12-MBs, which might be related to the additive mode effects of enhancers to increase expression (32, 81). In addition, the enrichment analysis of TF motifs showed that these enhancers have many binding sites of myogenic TFs, indicating its potential to bind to myogenic TFs to regulate gene expression. Moreover, we found that MYOG and MYOD1 TFs were colocalized in the enhancer sequence of *Myog*. This suggests that the expression of *Myog* is regulated by MYOG and MYOD1 during myogenic differentiation. Previous studies showed that *Myog* was activated by MYOD1 and that its expression was self-regulated (82, 83).

Gene KO is a traditional method of altering gene expression at the genome level. However, this method has a systemic and irreversible impact on the individual and can even cause death. As *cis*-regulatory elements, enhancers are usually located in noncoding regions to regulate gene expression in a spatiotemporally specific manner (81, 84). The manipulation of enhancers can regulate gene expression and reduce the impact on nontargeted tissues and cells. We used the dCas9-KRAB system to study the effect of enhancers on *Myog* expression and myogenic differentiation. After targeting the *Myog* enhancers, the expression of *Myog* was significantly decreased; moreover, we observed a reduction of MB fusion and MT formation by the repression of Myog-En4 and En5. This indicates that *Myog* expression and myogenic differentiation can be regulated by manipulating enhancers. Previous studies also reported that the manipulation of enhancer affected gene expression and changed cell differentiation processes. For example, deletion of the +6 kb enhancer of *CEBPE* reduced levels of CEBPE and inhibited granulocytic differentiation in mice (21). Although our study demonstrated the ability of four enhancers to regulate *Myog* expression, the modes of multiple enhancer action for gene expression require further in-depth study. Overall, our study demonstrated dynamic chromatin interactions of myogenic marker genes during myogenic differentiation and investigated the role of *Myog* enhancers in *Myog* expression. Our research strategy can be applied to other genes and is expected to greatly accelerate the study of mechanisms related to gene transcriptional regulation.

## Experimental procedures

### Cell culture and C2C12 myoblast differentiation

C2C12 mouse MB cells and human embryonic kidney cells (HEK293T) were kindly provided by the Cell Bank of the Chinese Academy of Sciences. HEK293FT cells were purchased from Thermo Fisher Scientific. Cells were maintained in Dulbecco's modified Eagle's medium (Gibco) supplemented with 10% fetal bovine serum (Gibco) and 1% penicillin–streptomycin (Gibco) at 37 °C under 5% CO_2_. The cells were confirmed without *mycoplasma* contamination using a PCR *mycoplasma* detection kit (TransGen). To induce differentiation, we cultured C2C12 cells in the differentiation medium consisting of 2% horse serum (HyClone) when grown to 50% to 60% confluence. The differentiation medium was changed every 48 h. Differentiated C2C12 cells are treated until day 5.

### Giemsa staining, immunofluorescence staining, and FI scoring

Cells on the dishes were fixed with 100% methanol for 2 to 3 min at room temperature (RT) and then washed with PBS. Giemsa stock solution (Solarbio) was diluted 1:9 in 0.01 M sodium phosphate buffer (pH 6.8), then cells were incubated with 1 ml of Giemsa working solution for 15 to 30 min at RT. Cells were finally visualized and photographed using an Olympus IX73 inverted microscope (Olympus). Each dish was photographed in three randomly selected regions. Protein-rich MTs are stained darker purple color, and nuclei are stained pink.

Cells were fixed in 4% paraformaldehyde in 0.1 M phosphate buffer (pH 7.4) for 10 min. Cells were washed three times with PBS and permeabilized by treatment with 0.25% Triton X-100 for 10 min. Cells were washed three times with PBS and blocked in 10% goat serum (Solarbio, SL038) for 40 min at RT. Cells were incubated with myosin 4 monoclonal antibody (eBioscience, 14-6503-80) overnight at 4 °C. Then, cells were washed three times with PBS and incubated with Alexa Fluor 488–labeled goat antimouse IgG H&L secondary antibody (abcom, ab150113) at RT protected from light for 1 h. Finally, the nuclei were stained with 1 μg/ml 4′,6-diamidino-2-phenylindole (Beyotime) for 3 min at RT. Cells were washed three times with PBS and photographed with Olympus IX73 inverted microscope (Olympus). The images were processed using ImageJ 1.52a software (NIH).

The FI was calculated as has been described (85). MTs were defined as 3+ nuclei within a cellular structure to rule out myoblasts undergoing mitosis. The number of nuclei in MTs with ≥ 3 nuclei and the total number of nuclei in cells were counted in each field. The FI was defined as the number of nuclei in MTs divided by the total number of nuclei.

### RNA extraction, complementary DNA synthesis, and quantitative RT-PCR

Total RNA was extracted using HiPure Total RNA Mini Kit (Magen) following the manufacturer’s instructions. Complementary DNA synthesis was performed with the HiScript III RT SuperMix (Vazyme). qRT-PCR amplification was performed on the CFX Connect Real-Time System (Bio-Rad) using the ChamQ Universal SYBR qPCR master mix (Vazyme). The reaction conditions were 95 °C for 5 min, followed by 40 cycles at 95 °C for 10 s and 60 °C for 30 s. After PCR amplification, a melting curve was obtained by the following process: 95 °C for 15 s, 60 °C for 1 min, followed by 95 °C for 15 s to verify primer specificity. Relative expression of genes was calculated using the 2^-ΔΔCt^ method (86) after normalization to mRNA expression of the housekeeping gene *β-actin*. All experiments were performed at least in triplicate. The primers of qRT-PCR are provided in Table S1.

### Generation of 4C libraries and sequencing

4C templates were prepared as previously described (24, 62) with slight modifications. The outline of the 4C-seq procedure, viewpoint selection, and primer position are shown in Fig. S1, *A* and *B*. In brief, ∼1 × 10^7^ million cells were harvested and crosslinked with 2% formaldehyde for 10 min at RT. The nuclei were digested using primary enzyme Dpn II (RE1) (New England Biolabs) overnight. Following proximity ligation, DNA was reverse crosslinked and purified using phenol/chloroform extraction, and the ligated circular DNA was precipitated with ethanol. The ligated circular DNA was digested with secondary enzyme Csp6 I (RE2) (New England Biolabs) overnight, followed by proximity ligation and purification to obtain the 4C library. The 4C-seq library was generated by performing a two-step PCR using Phusion DNA polymerase (Thermo Scientific; # F530S). The primers of the 4C-seq library are listed in Table S2. For each 4C-seq library, we perform 32-tube PCR reactions, and each PCR reaction uses 100 ng DNA as a template. PCR reactions were pooled and purified with the QIAquick PCR purification kit (Qiagen; #28104). DNA smears from 200 bp to 1000 bp were extracted by agarose gel and subjected to Agencourt AMPure XP Bead clean-up (Beckman Coulter; #A63881) using a 0.8 bead/DNA sample ratio. The 4C-seq libraries were sequenced on the Illumina NovaSeq 6000 platform.

### 4C-seq data analysis

Demultiplexing, trimming, mapping, and quality control of the 4C-seq data were performed using the pipe4C pipeline (62). r3Cseq (87) and PeakC (35) were used to perform 4C-seq data analysis. Briefly, trimmed reads were mapped to the masked version of the reference mouse genome (masked for the gap, repetitive, and ambiguous sequences) downloaded from the R Bioconductor repository (BSgenome.Mmusculus.UCSC.mm10.masked) using bowtie2 (v2.4.2). samtools (v1.11) was used to convert SAM to BAM. Only uniquely mapped reads consisting of sequences directly flanking the 4C primary restriction enzyme sites were used in downstream analyses, and nonunique frag-ends were discarded for posterior analysis.

Numbers of mapped reads for each window were counted and normalized to obtain reads per million per window values. Interactions sites were detected using r3Cseq with a nonoverlapping window (window size = 2 kb). Interactions sites with a *q*-value ≤ 0.05 were considered interaction sites. Interactions within ±500 kb around the viewpoint were visualized. The differential analysis of the interaction sites was performed using DESeq2 (version 1.35.0) with the “ashr” algorithm (88, 89). In addition, we used PeakC for calling peaks with a running mean window size of 11 (wSize = 11), as described previously (62, 90). Overlapping peak regions of two replicates were merged using bedtools (v2.25.0). Wig file of 4C-seq coverage profile was obtained using wSize = 21, and Wig file was visualized in Integrative Genomics Viewer.

### KEGG and GO enrichment analyses

The GO is a comprehensive source of digital data relating to the functions of genes, spanning three aspects of biology: BP (biological processes), CC (cellular components), and MF (molecular functions). The KEGG is a database resource for understanding high-level functions and utilities of the biological system from molecular-level information. Metascape (http://metascape.org/) is an online gene functional annotation tool to provide a comprehensive set of biological information of genes and proteins (91). To understand the function of genes, GO and KEGG enrichment analyses were performed using Metascape. GO/KEGG terms with *p* < 0.01 were considered significantly enriched.

### Download and analysis of public ChIP-seq data

Publicly available ChIP-seq sequencing data were downloaded from the EBI ENA database (https://www.ebi.ac.uk/ena/browser/home). The detailed information of ChIP-seq data is listed in Table S3.

For ChIP-seq data, raw sequence reads were aligned to the mouse reference genome (mm10) using BWA (v0.7.17), and BAM files were created by Samtools (v1.11). PCR duplicates of ChIP-seq were removed with Samtools. Prior to downstream analysis, mitochondrial reads were removed. Peak calling for individual replicates was carried out by MACS2 (v2.2.7.1). Peaks were called at a *q*-value ≤ 0.05. The bigwig files were generated by bedGraphToBigWig (v4) and visualized in Integrative Genomics Viewer (v 2.10.0).

### Identification of active enhancers of myogenic marker genes

The active enhancers were identified through two 4C-seq software combined with H3K27ac and H3K4me1 signal. We used two stringent criteria to identify active enhancers of myogenic marker genes. First, SISs identified by r3Cseq (87) show simultaneous enrichment with H3K27ac and H3K4me1 peaks; second, manually selected peaks based on chromatin interaction maps generated by PeakC (35) are overlaid simultaneously with H3K27ac and H3K4me1 peaks. To remove promoter regions (defined as -2000 bp to +500 bp around the transcription start site) of the annotated gene, we filtered out the enhancer located at the promoter regions. The active enhancers identified are considered same enhancer as long as their sequences are overlapped, and the manually selected enhancer is retained. The genomic coordinates of putative active enhancers are listed in Table S4.

### TF motif enrichment analysis and evolutionary conservation analysis

We used coordinates of the predicted enhancers to download FASTA format sequence of all the enhancers from the NCBI database (https://www.ncbi.nlm.nih.gov/). TF motif enrichment analysis was performed using AnimalTFDB 3.0 (http://bioinfo.life.hust.edu.cn/AnimalTFDB#!/tfbs_predict) (39) and JASPAR database (https://jaspar.genereg.net/) (40) by input FASTA format of the enhancers. TF motifs with *p* < 0.05 were identified as enriched motifs. The sequence logo of TF-binding sites motif was retrieved from JASPAR database.

The conserved elements of the predicted enhancers were analyzed by using phastCons method in the UCSC genome browser (http://genome-asia.ucsc.edu/). Each element is assigned a log-odds score (LOD) equal to its log probability under the conserved model minus its log probability under the nonconserved model. The “score” contains transformed log-odds scores, taking values between 0 and 1000. The raw log-odds scores are retained in the “name.” The sequence conservation is evaluated as the sum of the LOD of all elements divided by the length of the sequence, which is defined as the LOD score/length.

### Luciferase reporter assays

Putative active enhancers were amplified from C2C12 genomic DNA and cloned upstream of the luciferase gene in the pGL3-promoter vector (Promega) at the Kpn I site. The constructs were validated by Sanger sequencing. HEK293T cells were cotransfected with 95 ng of the tested construct and 5 ng of Renilla vector per well of 96-well plate using the Lipofectamine 3000 (Thermo Fisher Scientific). Thirty-six hours after transfection, luciferase activity was measured using the Dual-Glo luciferase assay kit (Promega; #E2920) on a GloMax 96 microplate luminometer (Promega) according to the manufacturer’s instructions. Luciferase activity of firefly was normalized by renilla, and relative luciferase activity was obtained as compared with empty pGL3-promoter. The experiments were in triplicate and repeated at least once. The data are presented as means with their SD (mean ± SD). The PCR primers of constructed vectors are shown in Table S5.

### sgRNA design and plasmid construction

Two sgRNAs were designed for each enhancer using CRISPOR (http://crispor.tefor.net/) (92) and CRISPR-ERA (http://crispr-era.stanford.edu/) (93). All sgRNAs were examined for genome-wide sequence specificity using CRISPR Finder (https://wge.stemcell.sanger.ac.uk/) (94) and Cas-OFFinder (http://www.rgenome.net/cas-offinder/) (95). Synthesized oligonucleotides were annealed and cloned into the BsmBI-v2 restriction sites of pLV hU6-sgRNA hUbC-dCas9-KRAB-T2a-Puro vector (Addgene; #71236) using DNA Ligation Kit (Takara). Sanger sequencing confirmed sgRNAs insertion. The sequences of all the sgRNAs are listed in Table S6.

### Lentivirus production, cell transduction, and CRISPRi-mediated enhancer inhibition

Under sterile BSL-2 conditions, lentiviral particles were generated in HEK293FT cells using the pVSV-G (Addgene; #138479) and psPAX2 (Addgene; #12260) packaging plasmids alongside the Lenti-dCas9-KRAB-enhancer-sgRNA plasmid (Addgene; #71236). The virus supernatant was harvested at 24, 48, and 72 h post-transfection and filtered with a 0.45 μm polyvinylidene difluoride filter (Millipore; catalog no.: #SLHV033RB). Viruses were purified and concentrated using Amicon Ultra (Millipore; #UFC910008) and titer was determined using a colloidal gold kit (BioDragon). Concentrated viruses were stocked in a final volume of 1 ml and stored at −80 °C.

For transduction, C2C12 cells were seeded in a 12-well plate. When confluence reaches about 50%, the cells were infected with 1 ml lentiviruses medium containing dCas9-KRAB-sgRNA or dCas9-KRAB at a multiplicity of infection of 100. The day after transduction 24 h, the medium was exchanged to remove the virus. At 48 h postinfection, 2 μg/ml puromycin was used to select transduced cells. C2C12 cells were kept at ∼30% confluence during antibiotic selection. C2C12 cells stably expressing dCas9-KRAB-sgRNAs or dCas9-KRAB were induced to differentiation with the differentiation medium consisting of 2% horse serum for 5 days. C2C12 cells expressing dCas9-KRAB served as controls. Cells were examined by qRT-PCR and immunofluorescence staining.

### RNA-Seq: Library preparation, sequencing, and data analysis

Total RNA (RIN ≥ 9) was used as input material for the RNA sample preparations. Sequencing libraries were generated using NEBNext UltraTM RNA Library Prep Kit for Illumina (NEB). mRNA was purified from total RNA using poly-T oligo-attached magnetic beads. The library preparations were sequenced on an Illumina Novaseq 6000 platform and 150 bp paired-end reads were generated. Sequence reads were aligned to the mouse reference genome (GRCm38/mm10) by HISAT2 (v2.2.1) and quantified by featureCounts from the Rsubread package (v2.8.1). Differential analysis was performed using DESeq2. DEGs were defined as genes with adjusted *p*-value ≤ 0.01 and |Log_2_fold-change| ≥ 1. Gene expression levels for each sample were calculated as transcripts per million values. Functional enrichment analysis of the DEGs was performed using Metascape. Terms with *p*-values ≤ 0.01 were considered significantly enriched for DEGs. GSEA was performed using GSEA version 4.2.3 software (https://www.gsea-msigdb.org/gsea/index.jsp) with MSigDBv7.5.1.

### Statistical analysis

Statistical analysis was performed for unpaired or paired two-tailed Student’s *t* test using SPSS Statistics 21. Data are presented as mean ± SD. *p* ≤ 0.05 was considered statistically significant.

## Data availability

All 4C-seq data were deposited in the NCBI Sequence Read Archive (SRA; https://www.ncbi.nlm.nih.gov/sra/) under BioProject PRJNA795427. RNA-seq data were deposited into the Gene Expression Omnibus (GEO) under accession code GSE201138.

## Supporting information

This article contains supporting information.

## Conflict of interest

The authors declare that they have no conflicts of interest with the contents of this article.

## Supporting information

Supplementary Figure S1A-B

Supplementary Figure S2A-C

Supplementary Figure S3A-D

Supplementary Figure S15

Supplementary Table1

Supplementary Table2

Supplementary Table3

Supplementary Table4

Supplementary Table5

Supplementary Table6

Supplementary Table7

Supplementary Table8

Supplementary Table9

Supplementary Table10

Supplementary Table11

Supplementary Table12

Supplementary Table13

Supplementary Table14

Supporting Information Text_Second Submission
