# Supplementary material for: Exploring high-resolution chromatin interaction changes and functional enhancers of myogenic marker genes during myogenic differentiation
Source: J Biol Chem. 2022 Jul 2;298(8):102149. doi: 10.1016/j.jbc.2022.102149 (PMC9352921; doi:10.1016/j.jbc.2022.102149)
Supplement: Supplementary Figure S15 [file mmc15.docx]

**Supporting information**

**Figure S1.** (A) Schematic workflow of the 4C-seq procedure. Chromatin in the nucleus is fixated by formaldehyde. The chromatin is digested using the primary restriction enzyme (RE1: DpnII) and ligated under dilute conditions. After reversal of the crosslinks, the purified DNA is digested by a second restriction enzyme (RE2: Csp6I) and religated under diluted conditions to create small DNA circles. Inverse PCR and index PCR are performed to capture the ligated DNA sequences and construct a 4C sequencing library. (B) Schematic viewpoint selection of eight myogenic marker genes.

**Figure S2.** (A) Images of C2C12 myoblasts proliferation and differentiation for 5 days. Representative Giemsa staining images of myoblasts differentiation. Black arrows indicate myotubes; red arrows indicate pink nuclei. Immunofluorescence staining images of myoblasts differentiation. Myotubes were stained with anti-myosin heavy chain (anti-MHC) antibody (green) and cell nuclei with DAPI (blue). (B) Fusion index of myoblast differentiation for 5 days between replicates. Fusion index was calculated as the percentage of total nuclei that resided in cells containing three or more nuclei. (C) Relative expression levels of myogenic marker genes in C2C12-MBs and C2C12-MTs. Relative expression levels of genes (*Myod1*, *Myog*, *Mef2a*, *Mef2b*, *Mef2d*, *Myh2*, *Myh3*, and *Mymk*) were normalized to *β-actin* using 2^-∆∆CT^ method. Results are expressed as mean ± SD (n = 3), ****P* < 0.005.

**Figure S3.** (A) Bar plot showing the percentage of mapped reads in *cis*-chromosome of each 4C data. (B) Bar plot showing the percentage of all unique fragment ends at least one mapped read within ± 100 kb of the viewpoint of each 4C data. (C) Bar plot showing the percentage of reads mapped to unique fragment ends within ± 1 Mb of the viewpoint of each 4C data.

**Figure S4.** Scatter plot showing interactions between replicates of *Myog*, *Mef2a*, *Mef2b*, *Mef2d*, *Myh2*, *Myh3*, and *Mymk*. The numbers of interaction sites (in Log_2_) of each 1Mb *cis* in two replicates are plotted. The Pearson correlation coefficient is shown in the panel.

**Figure S5.** Circos plots of genome-wide interaction sites of *Myog*, *Mef2a*, *Mef2b*, *Mef2d*, *Myh3*, and *Mymk*. Chromosomes are shown in a circular orientation. The numbers and letters above the circle indicate the names of the chromosomes.

**Figure S6.** (A) Heatmap showing the clustering of *Myog*, *Mef2a*, *Mef2b*, *Mef2d*, *Myh2*, *Myh3*, and *Mymk* interactions in C2C12-MBs and C2C12-MTs. The color scale indicates the degree of correlation (blue, low correlation; red, high correlation). (B) Principal component analysis results. PCA plot showing the variance between each 4C sample. Samples with similar interactions are clustered together. The sample is indicated by using different colors, as shown in the legend provided. The percentages on each axis represent the percentages of variation explained by the principal components.

**Figure S7.** The discrepancy of chromatin interaction of *Myod1*, *Myog*, *Mef2a*, *Mef2d*, *Myh2*, *Myh3*, and *Mymk* within ±500 kb of the viewpoint in C2C12-MBs and C2C12-MTs. Circles represent interactions. The dotted red line represents the viewpoint. The deeper color indicates a higher interaction frequency. The y-axis indicated reads per million (RPM).

**Figure S8.** (A) Volcano plot of SDISs of *Mef2a*, *Mef2b*, *Mef2d*, *Myh2*, *Myh3*, and *Mymk*. The threshold of SDISs in the volcano plot was -Log_10_(*q*-value) ≥ 2. Red and blue dots indicate significantly differential interaction sites. (B) The average |Log_2_FC| of SDISs within ±500 kb of myogenic marker genes.

**Figure S9.** (A) A density plot showing the |Log_2_FC| distribution of up- and down-regulated SDISs. (B) A density plot showing the |Log_2_FC| distribution of *cis*- and *trans*-SDISs.

**Figure S10.** (A) The peaks numbers of the myogenic marker genes in C2C12-MBs and C2C12-MTs. The peaks were identified by PeakC (wSize = 5). (B) Venn diagram showing the common and unique SISs of the myogenic marker genes in C2C12-MBs and C2C12-MTs.

**Figure S11.** Histone modification of chromatin interaction sites of the *Myod1*, *Mef2a*, *Mef2b*, *Mef2d*, *Myh2*, *Myh3*, and *Mymk* within ±500 kb of the viewpoint in C2C12-MTs. Red circles represent interactions in C2C12-MTs. The dotted red line represents the viewpoint. ChIP-seq profiles for H3K27ac (blue), H3K4me2 (yellow), H3K4me3 (cyan), H3K36me3 (purple), and H3K27me3 (orange) in C2C12-MTs. The chromatin interactions of *Myod1*, *Mef2a*, *Mef2d*, *Myh2*, *Myh3*, and *Mymk* in C2C12-MTs are reused in Figure S7 and Figure S11.

**Figure S12.** Conservation analysis of putative active enhancers of the myogenesis marker genes. UCSC genome browser (http://genome-asia.ucsc.edu/; GRCm38/mm10) showing conservation of putative active enhancers in selected species, such as human, chimp, macaque, panda, elephant, pig, dog, rat, and naked mole-rat. Horizontal red bars indicate conservative elements in 60 vertebrates. Elements conservation is measured as the LOD score of phastCons elements.

**Figure S13.** The cohesin-mediated loop might be responsible for Myog-Ens and *Myog* promoter interaction. Alignment of Hi-C data from C2C12 muscle cells, 4C-seq data from C2C12-MTs, ChIP-seq data of H3K27ac and H3K4me1 from C2C12-MTs, and ChIP-seq data of SMC3 (a subunit of the cohesin complex) and CTCF from mouse muscle tissues or cells in the *Myog* locus. Hi-C heatmap (upper panel) showing that Myog-Ens and *Myog* promoter are organized in an interaction domain. Lower panel showing 4C-seq data (black), ChIP-seq data of H3K27ac (blue) and H3K4me1 (green), and ChIP-seq data of CTCF (dark cyan) and SMC3 (red). The orange column represents the *Myog* enhancers. The red dotted line represents the viewpoint. CTCF-motif position and orientation are indicated by arrows (red arrow: forward core motif, black arrow: reverse core motif). The gray column represents the chromatin loop anchors.

**Figure S14.** Selection of puromycin-resistant C2C12 cells expressing dCas9-KRAB-sgRNAs system. (A) Proliferating C2C12 cells expressing dCas9-KRAB-En1-sgRNAs, dCas9-KRAB-En3-sgRNAs or dCas9-KRAB-En5-sgRNAs system after 10 days of puromycin selection. (B) Amplification curve of *dCas9* and *β-actin* in dCas9-KRAB cells and dCas9-KRAB-sgRNAs cells. (C) Melt curve of *dCas9* and *β-actin* in dCas9-KRAB cells and dCas9-KRAB-sgRNAs cells.

**Figure S15.** GSEA of all expressed genes shows enrichment of genes involved in muscle development and structural constituent in dCas9-KRAB cells.
