# Supplementary figures and images for: Exploring high-resolution chromatin interaction changes and functional enhancers of myogenic marker genes during myogenic differentiation
Source: J Biol Chem. 2022 Jul 2;298(8):102149. doi: 10.1016/j.jbc.2022.102149 (PMC9352921; doi:10.1016/j.jbc.2022.102149)

A

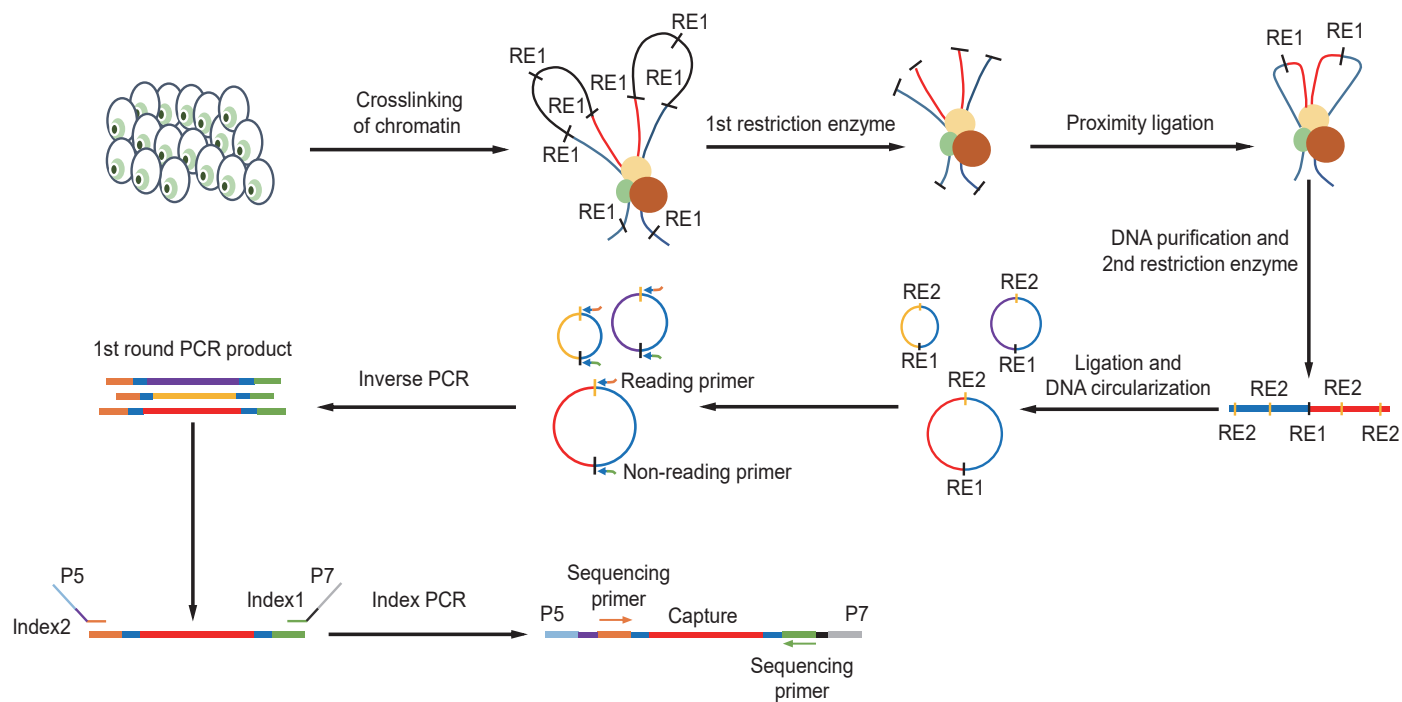

B

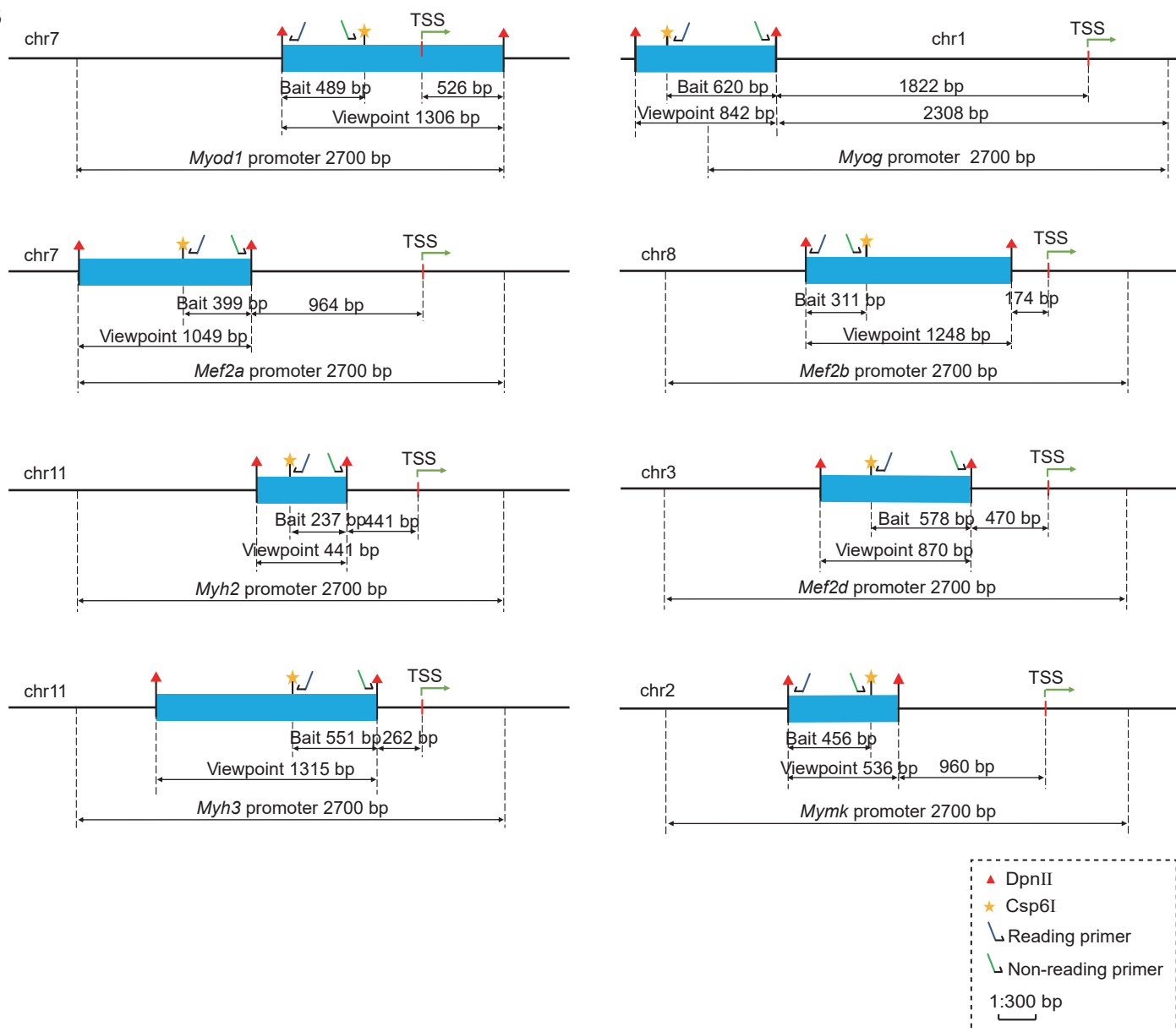

Supplement: Supplementary Table1 [file mmc16.pdf]

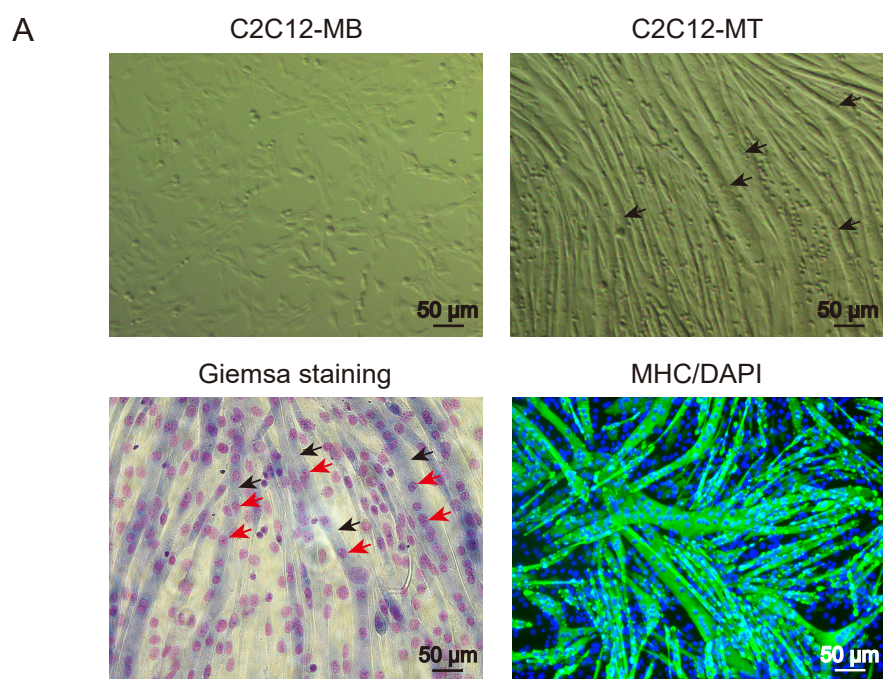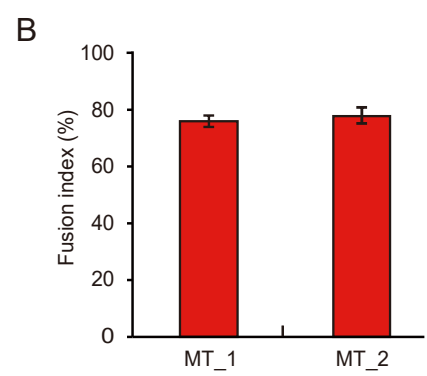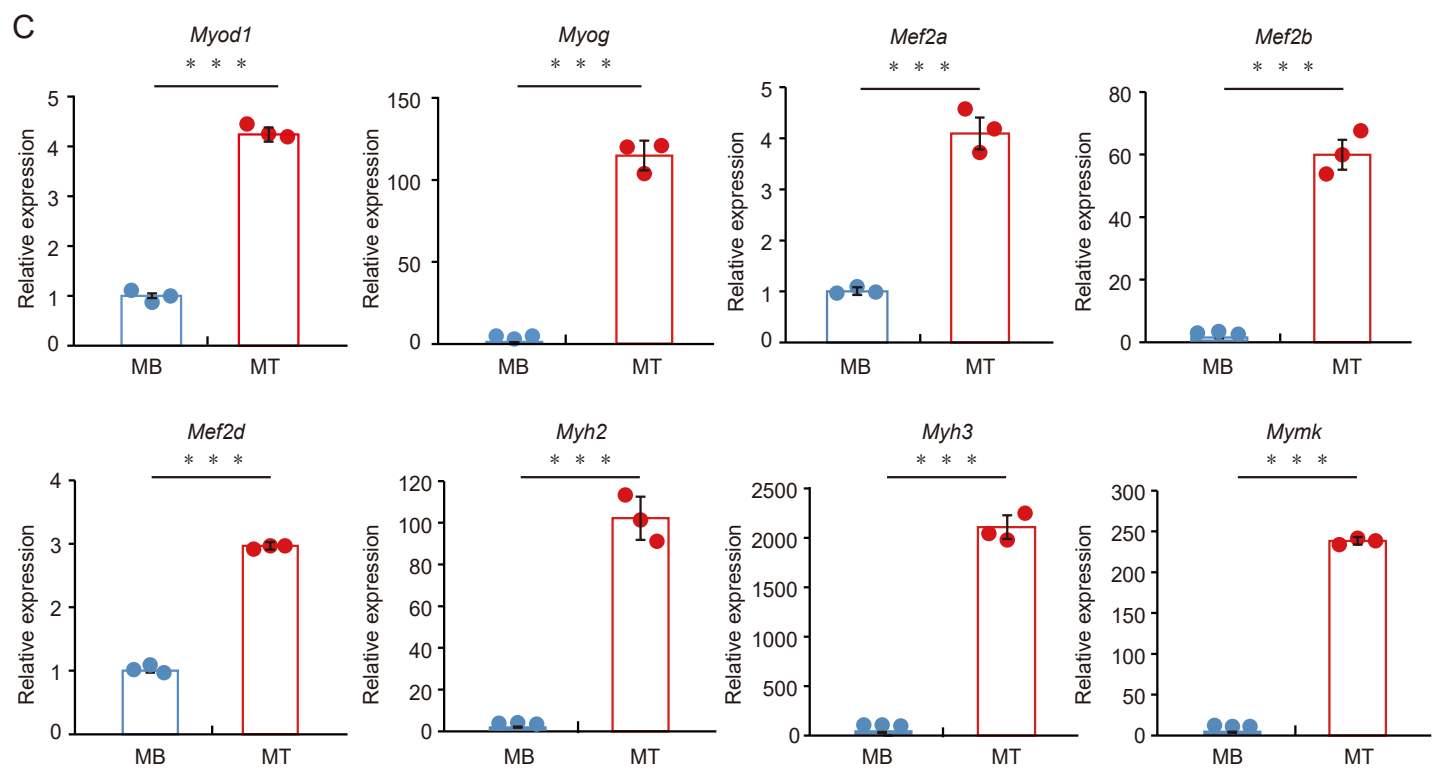

Supplement: Supplementary Table2 [file mmc17.pdf]

A

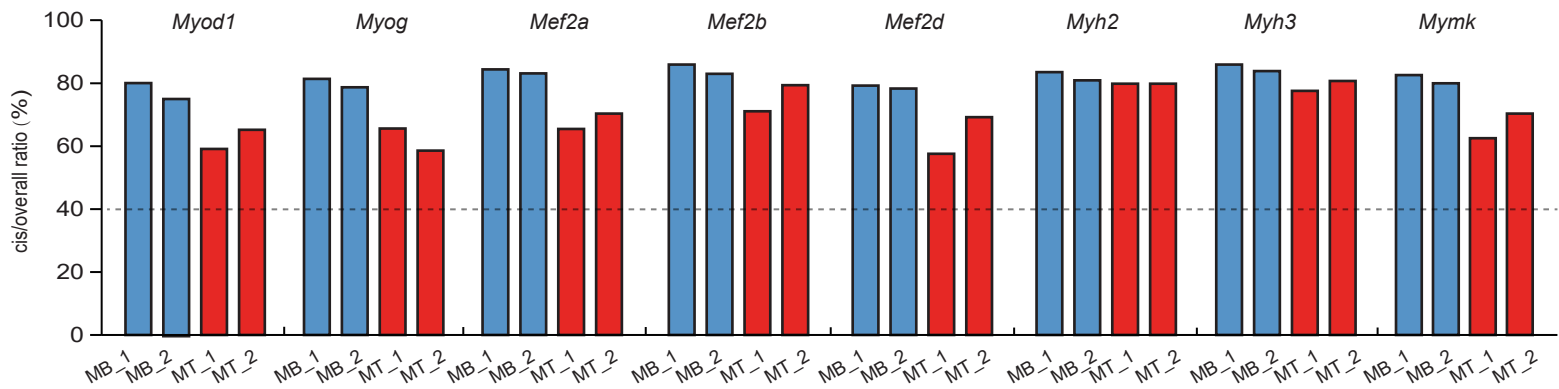

B

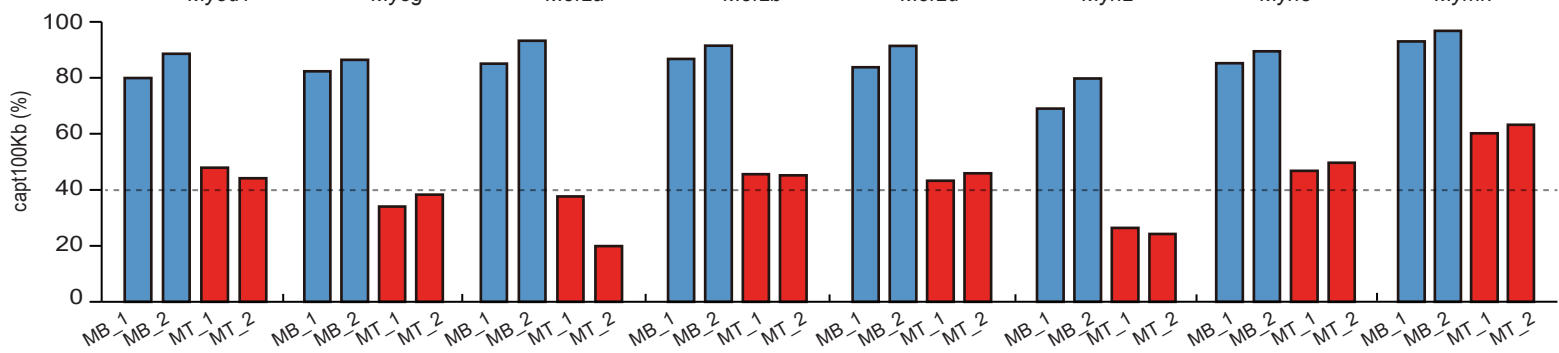

C

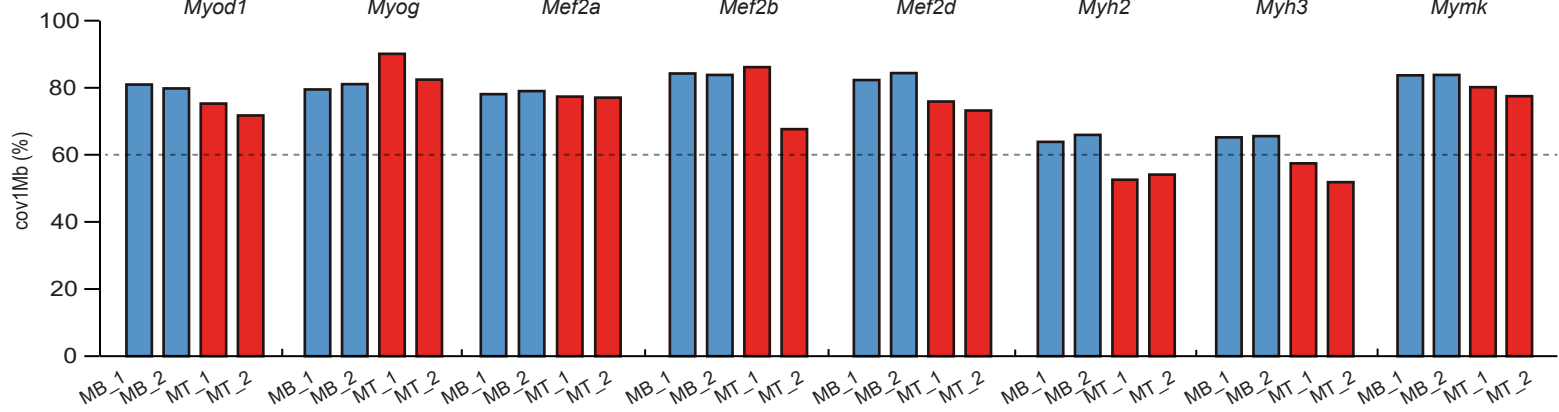

Supplement: Supplementary Table3 [file mmc18.pdf]

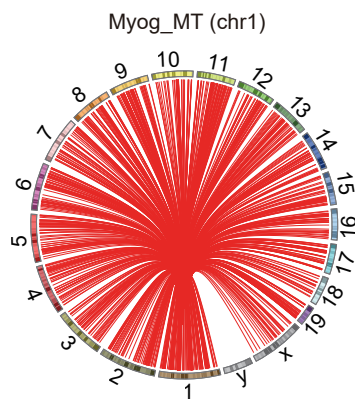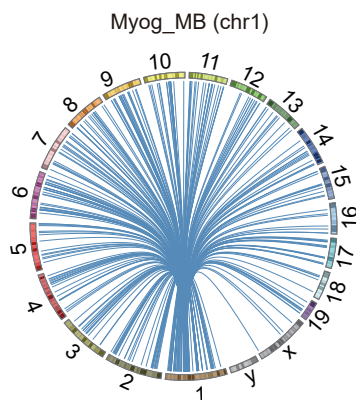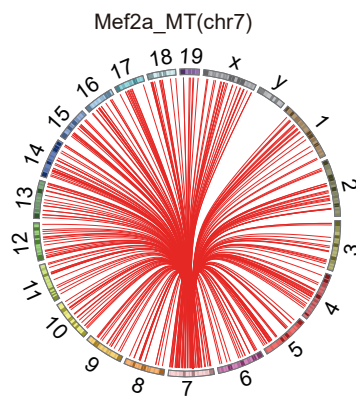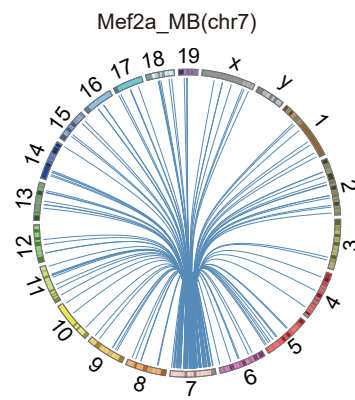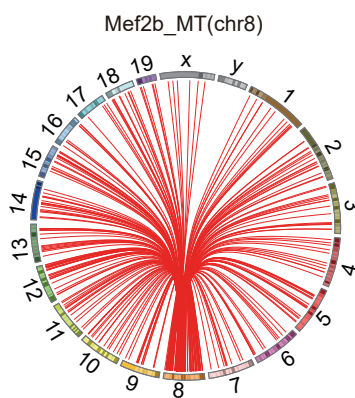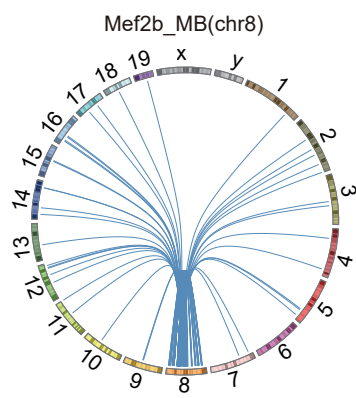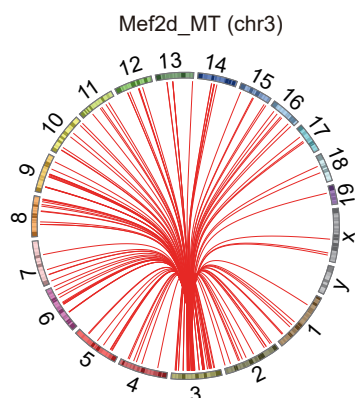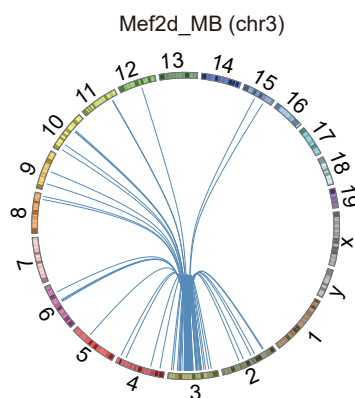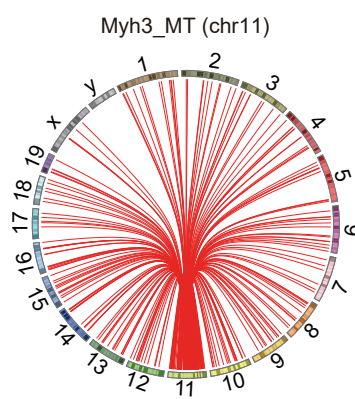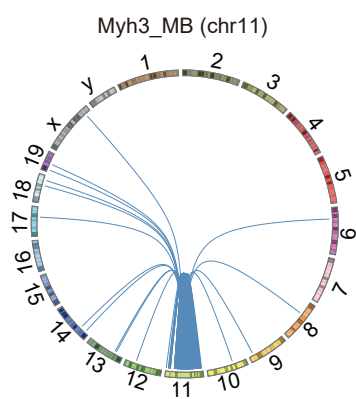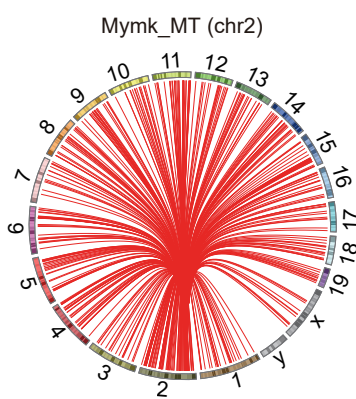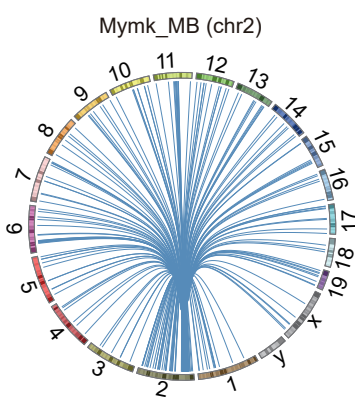

Supplement: Supplementary Table5 [file mmc20.pdf]

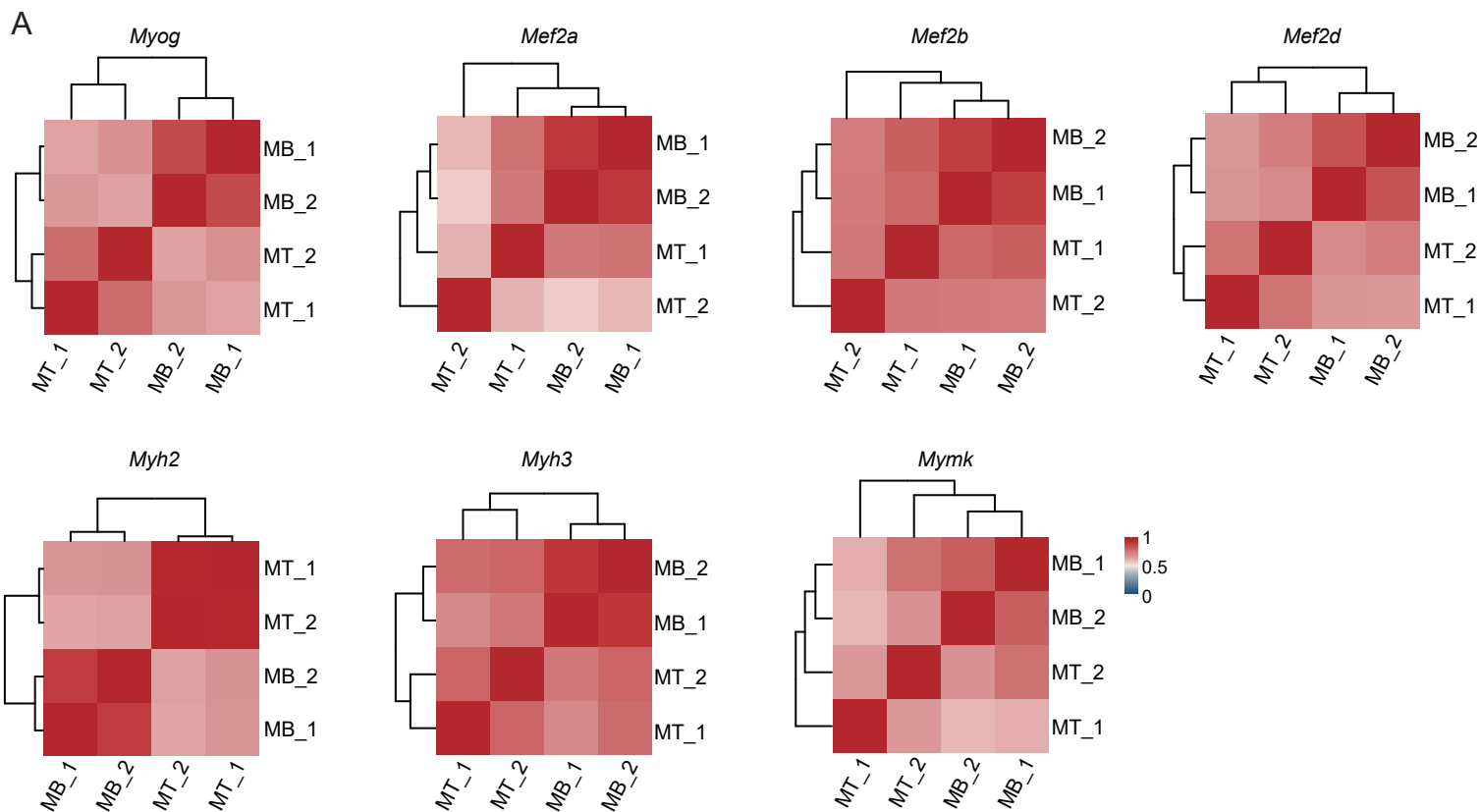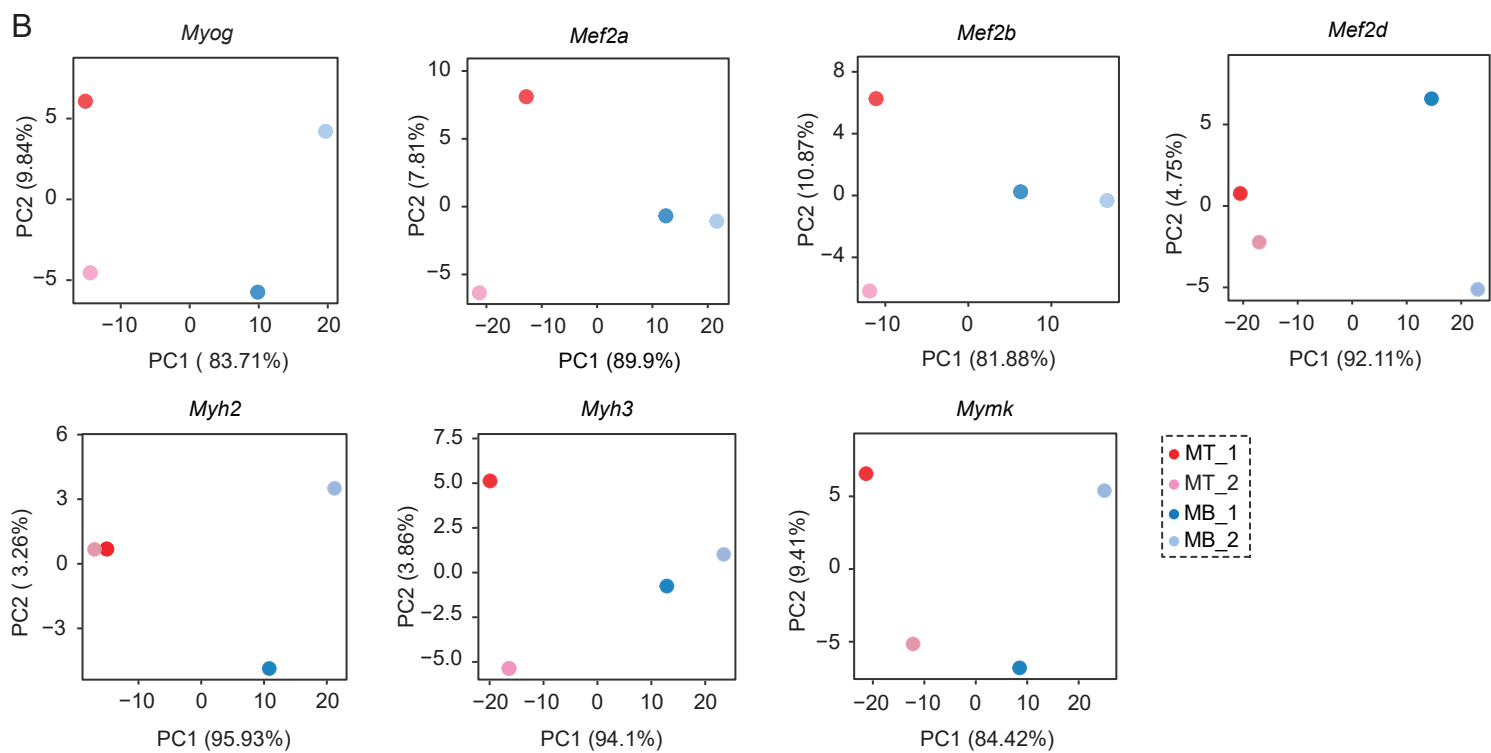

Supplement: Supplementary Table6 [file mmc21.pdf]

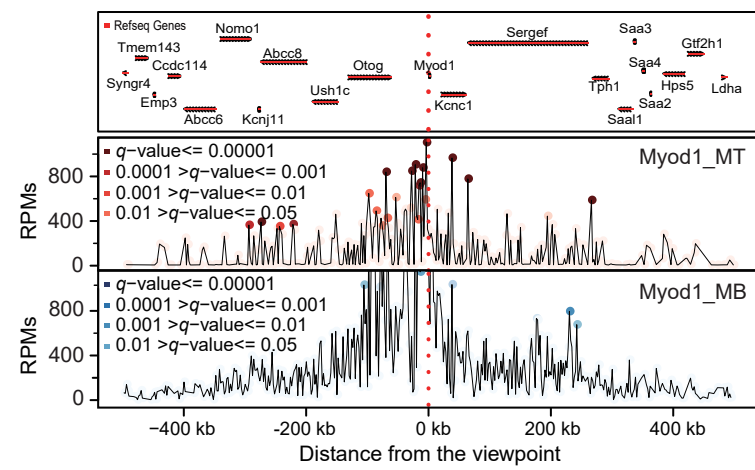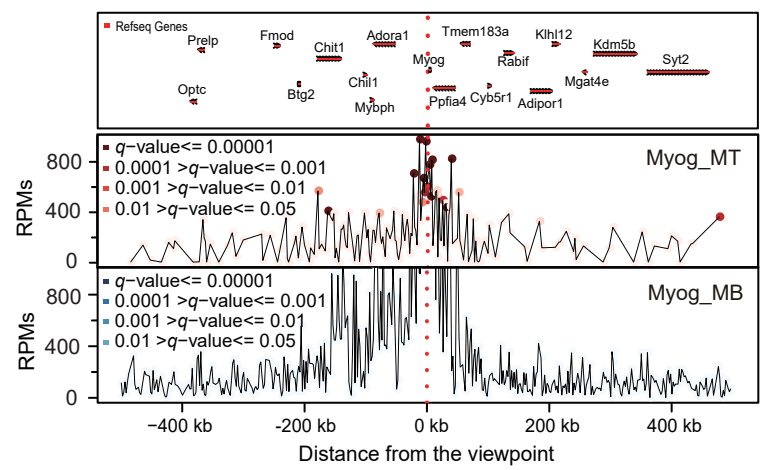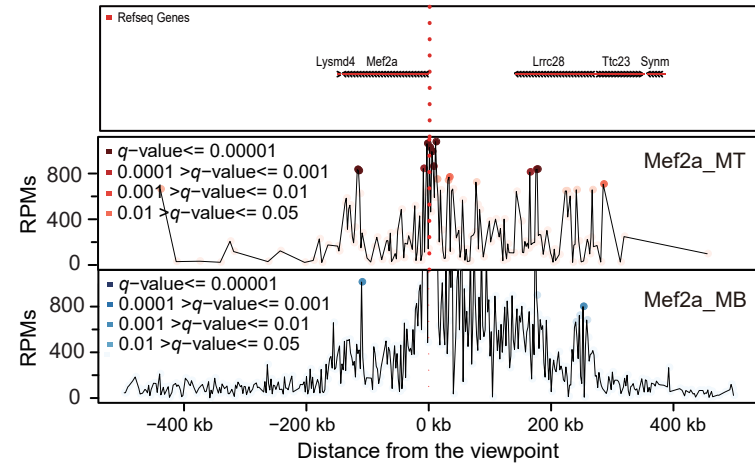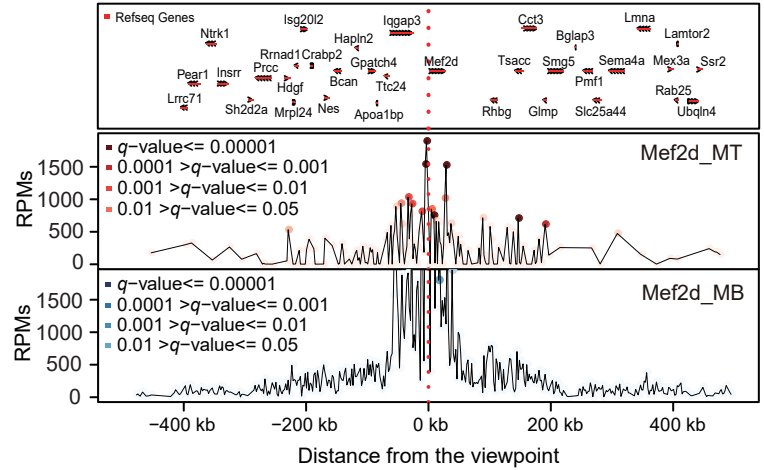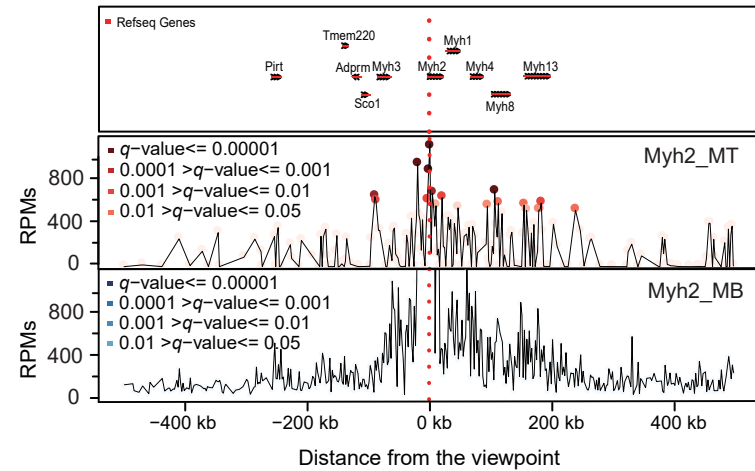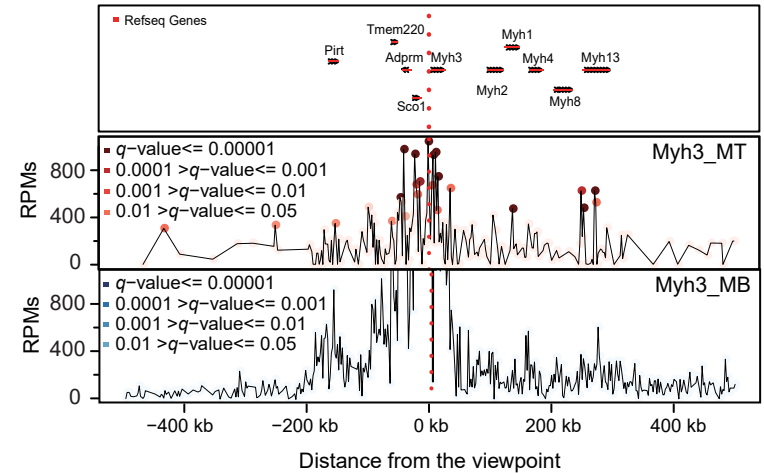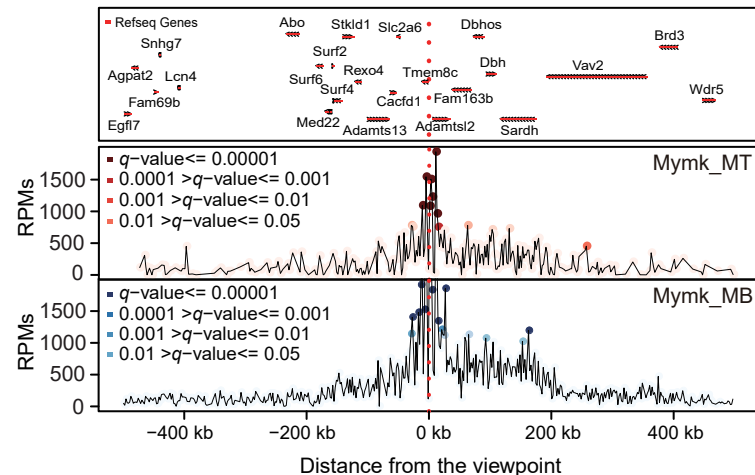

Supplement: Supplementary Table7 [file mmc22.pdf]

A

*Mef2a*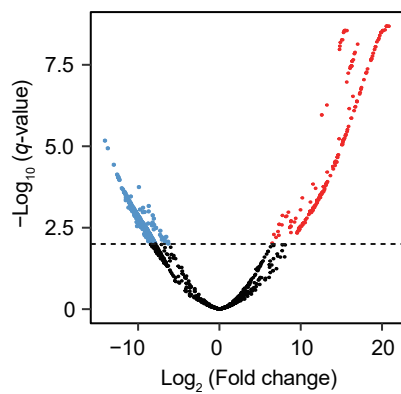*Mef2b*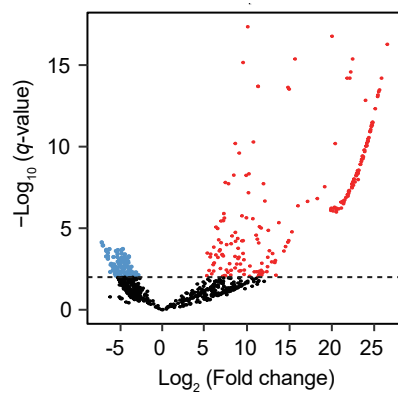*Mef2d*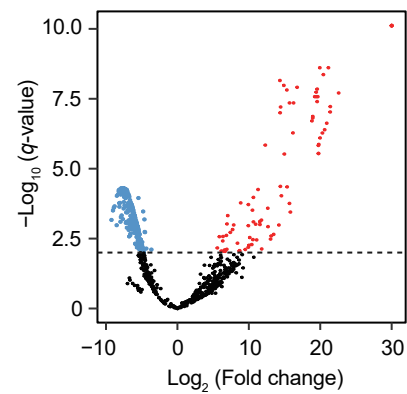*Myh2*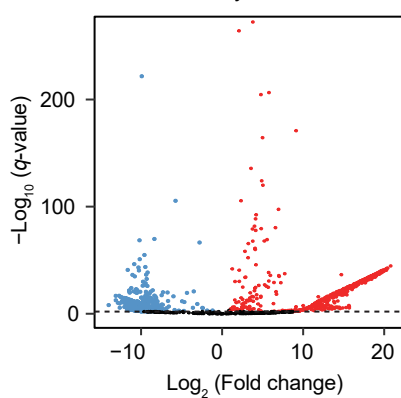*Myh3*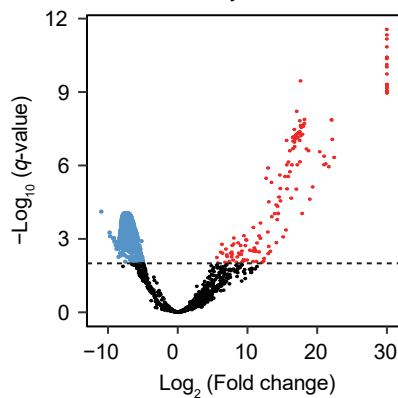*Mymk*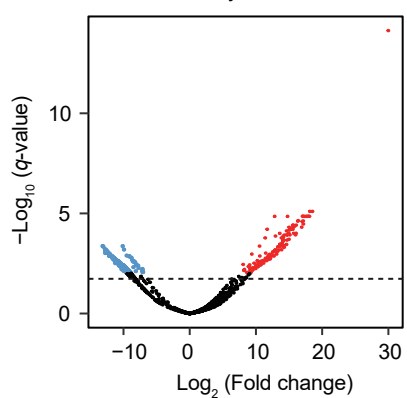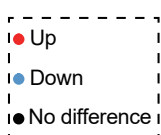

B

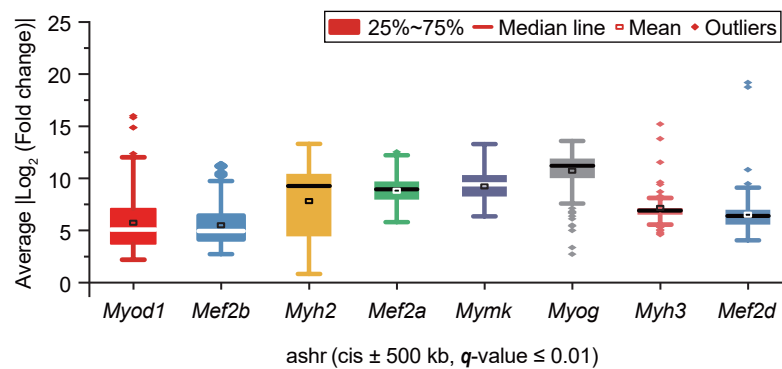

Supplement: Supplementary Table8 [file mmc23.pdf]

A

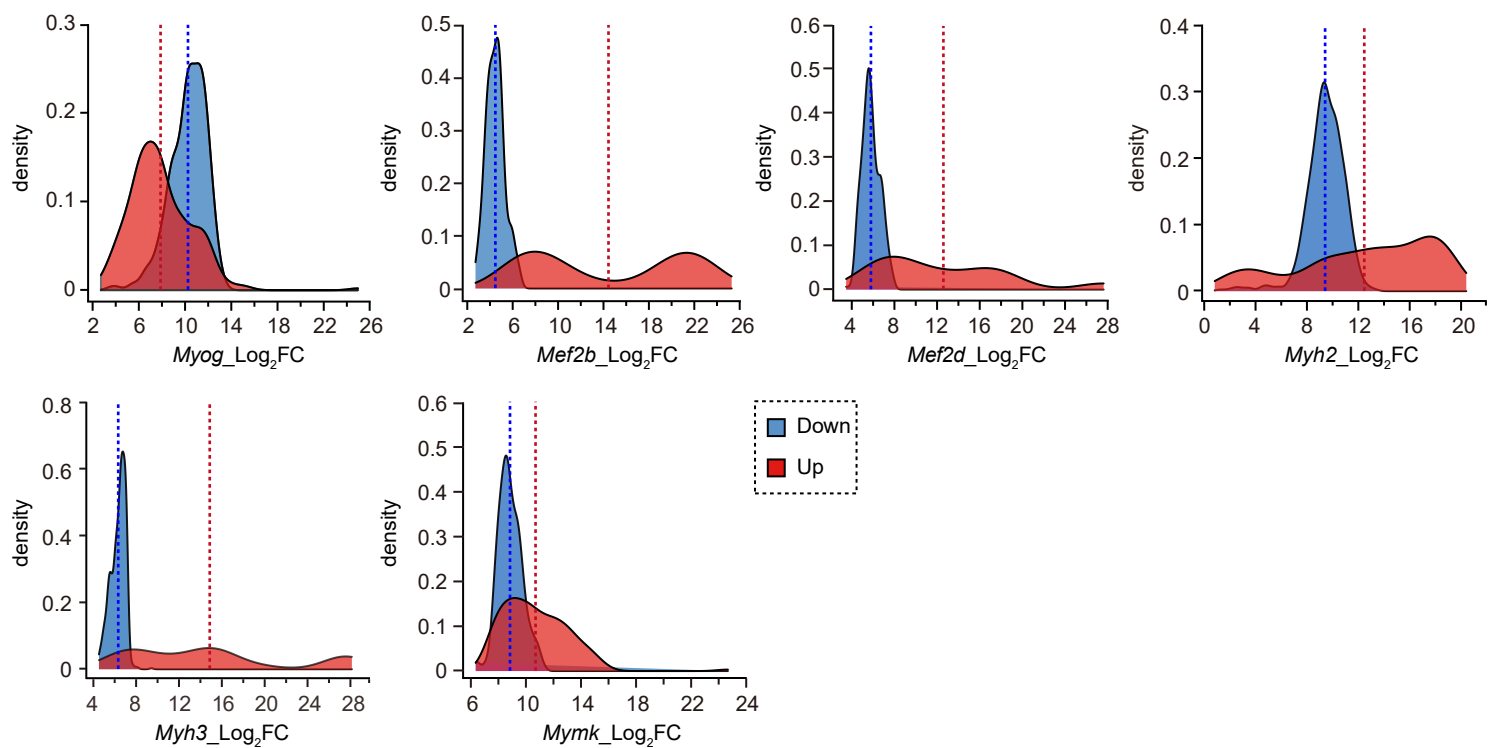

B

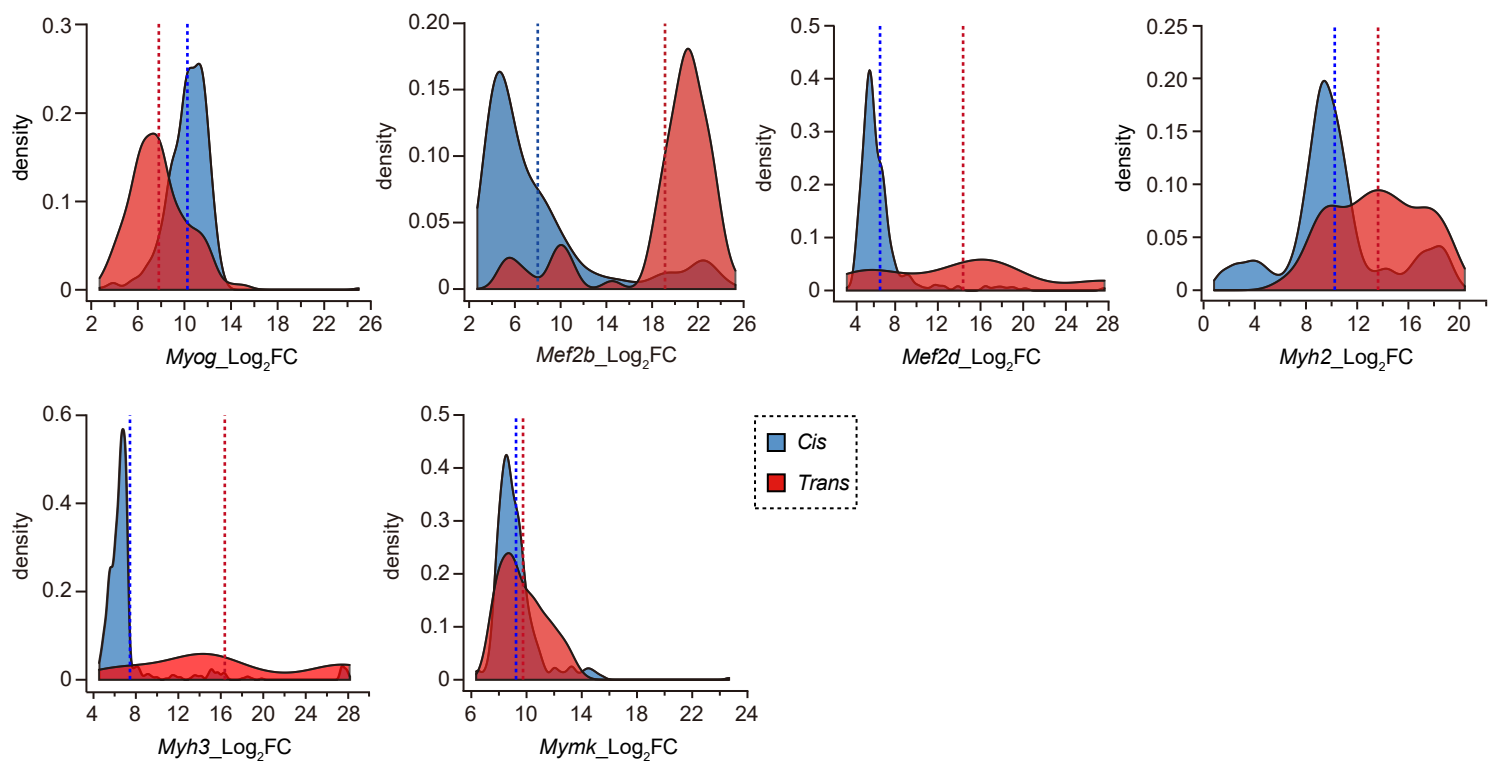

Supplement: Supplementary Table9 [file mmc24.pdf]

A

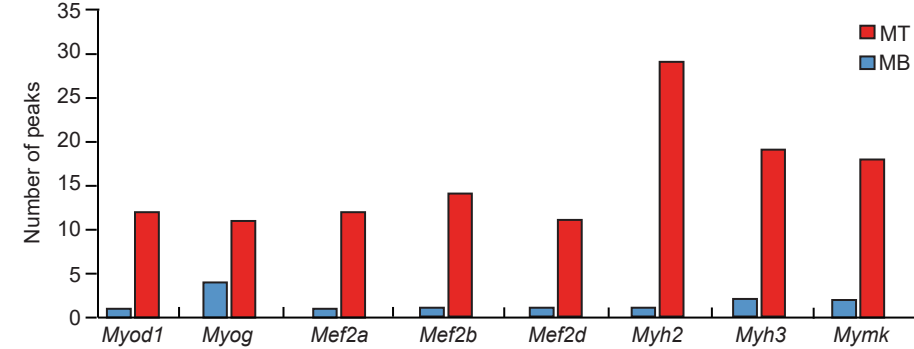

B

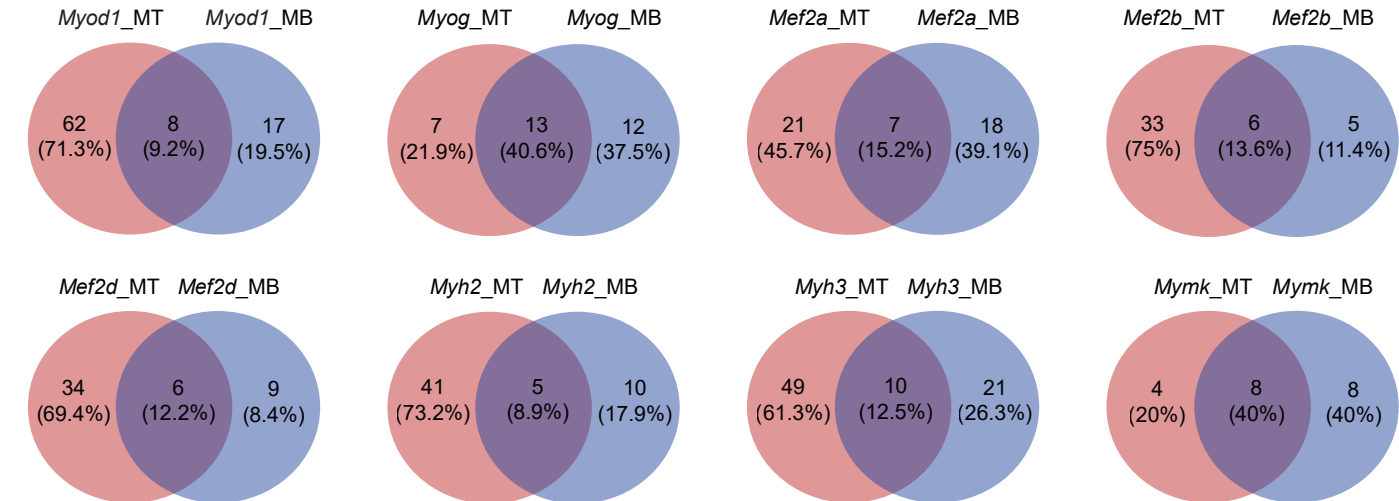

Supplement: Supplementary Table10 [file mmc25.pdf]

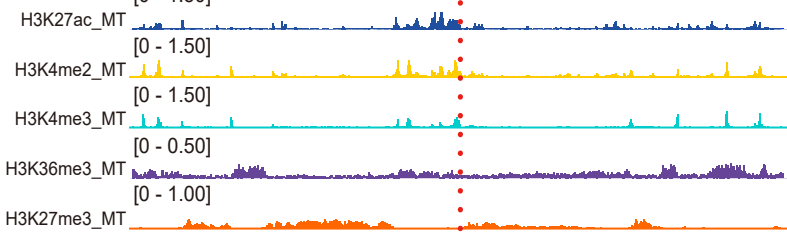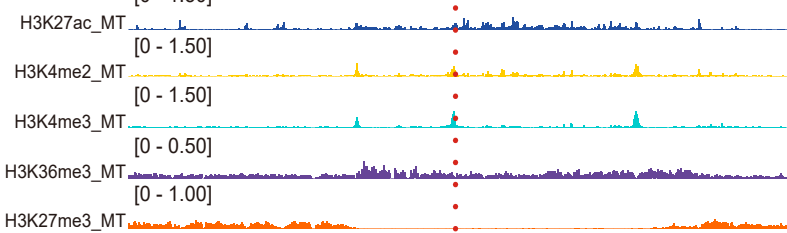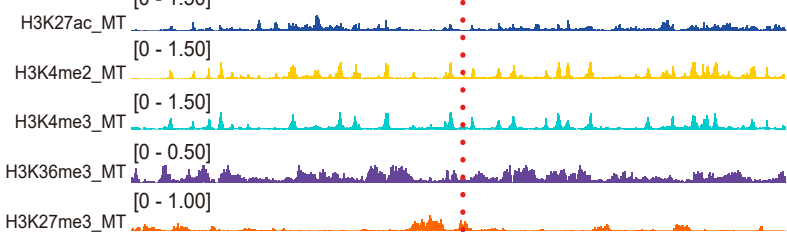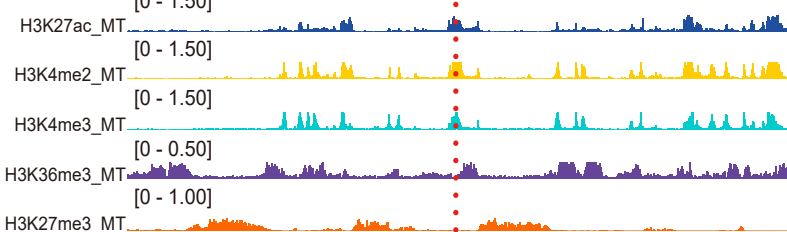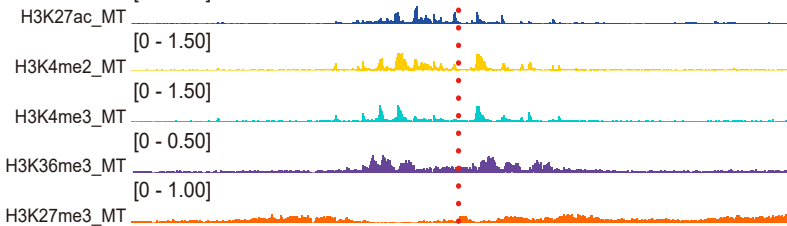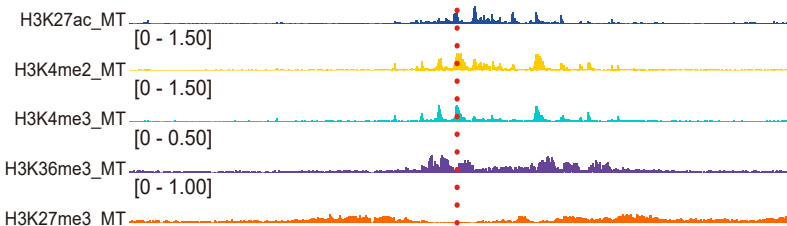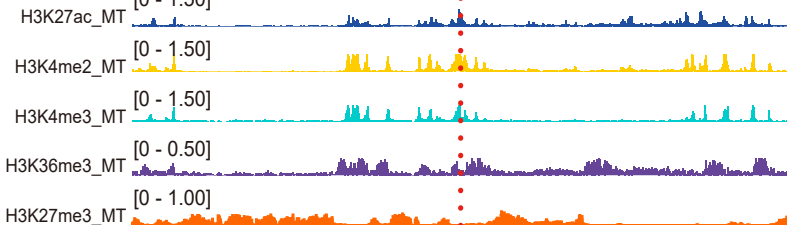

Supplement: Supplementary Table11 [file mmc26.pdf]

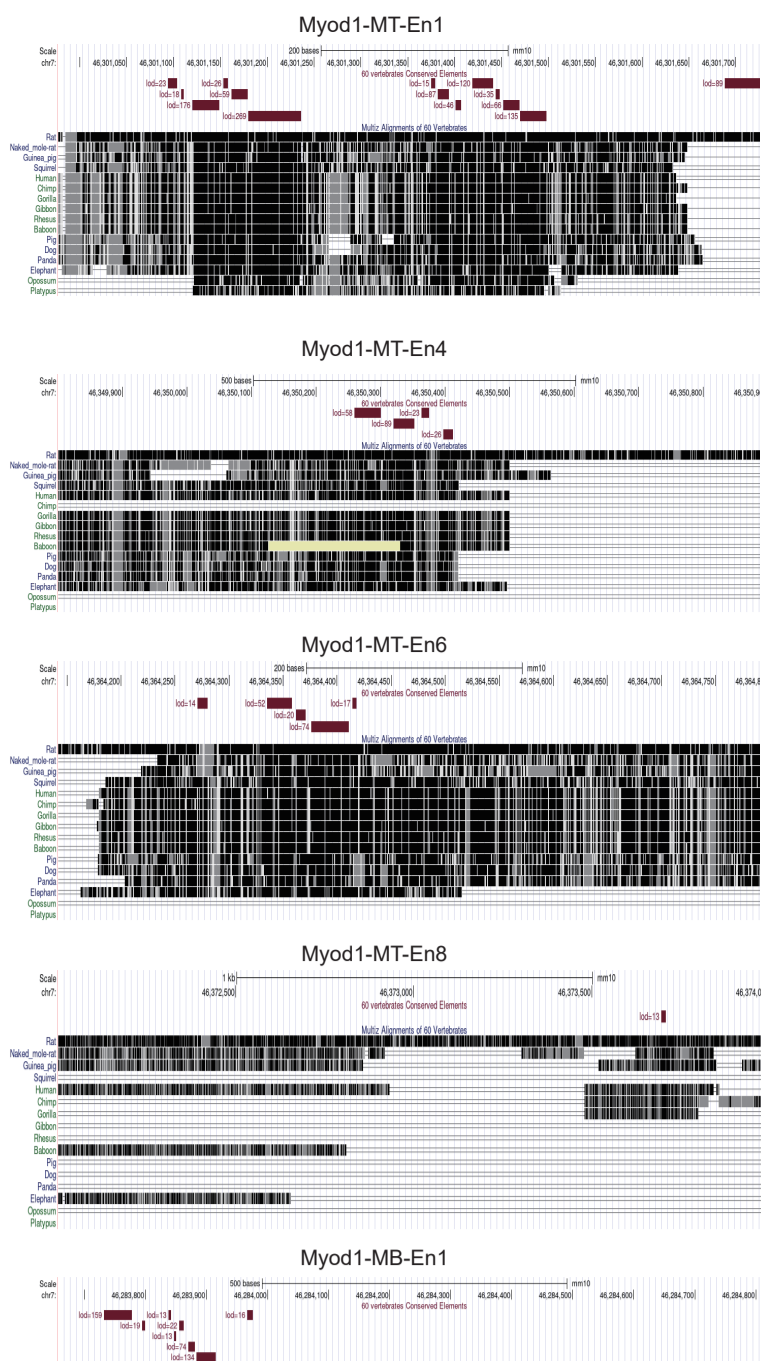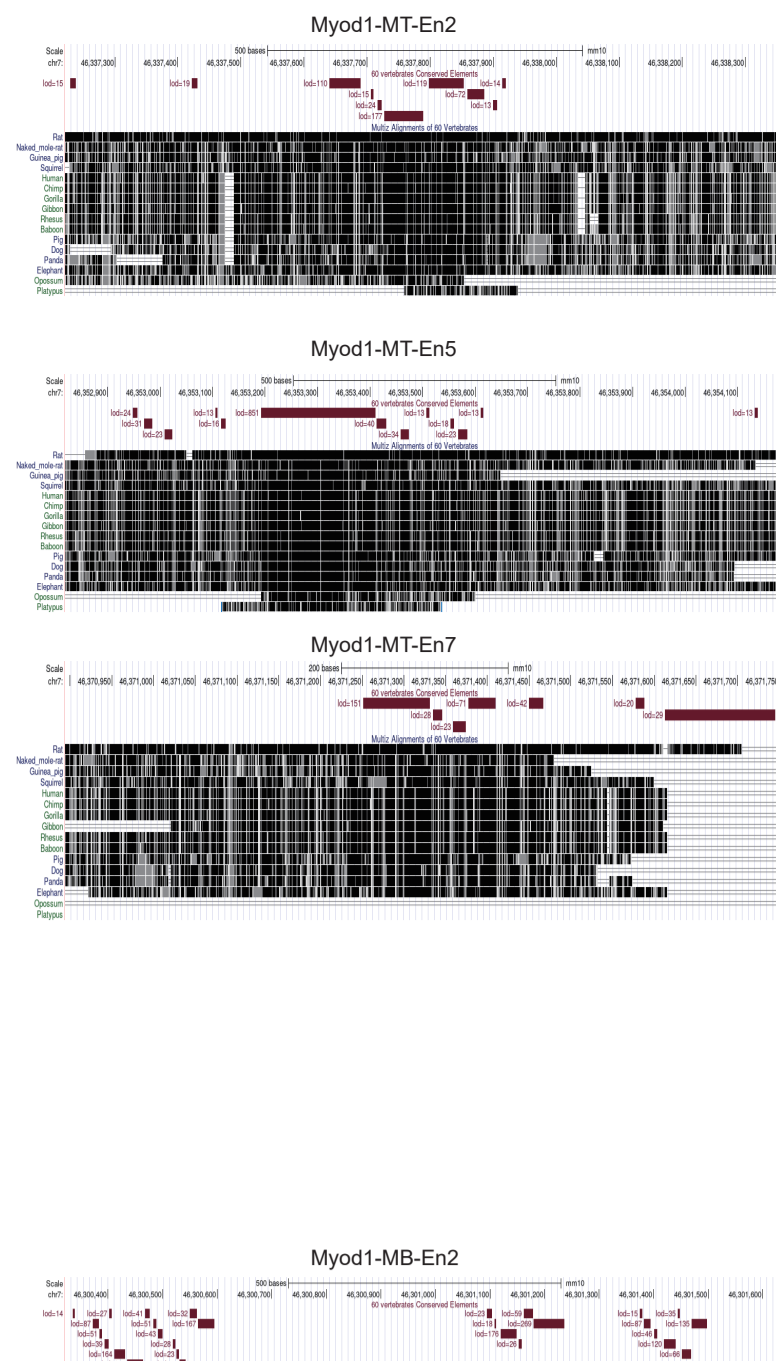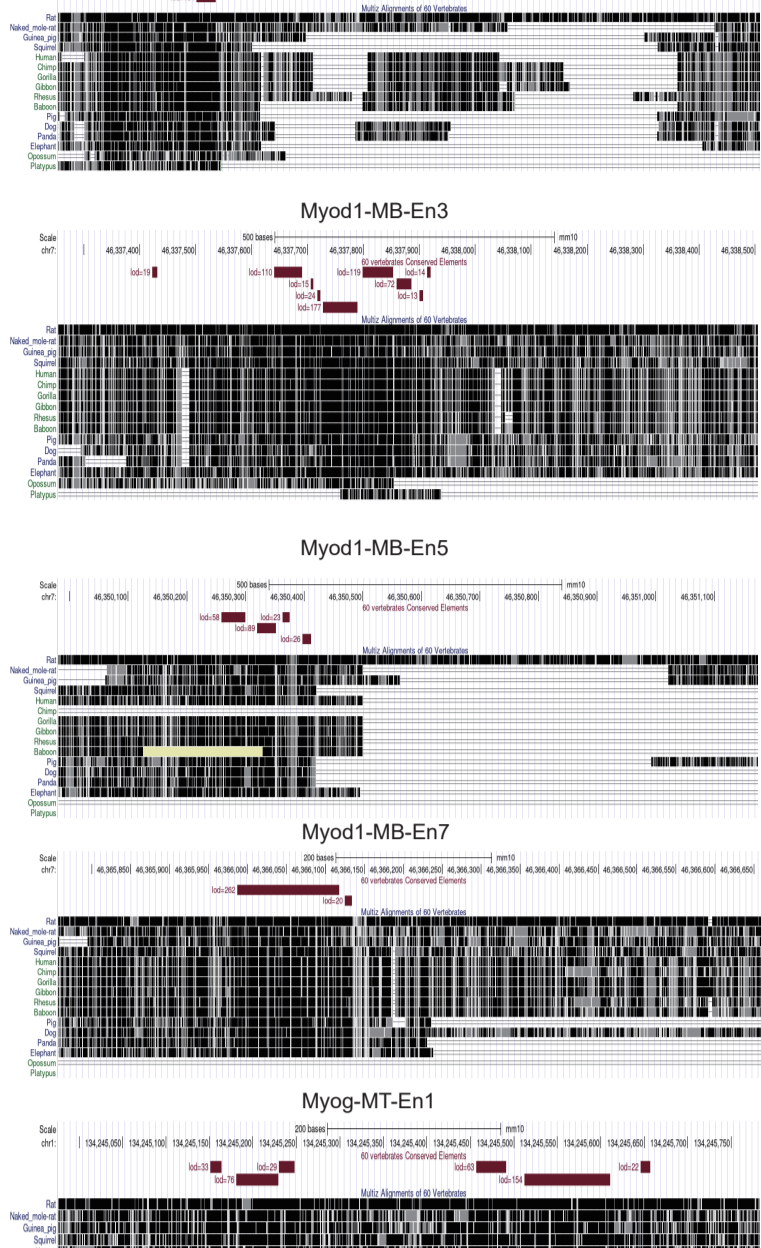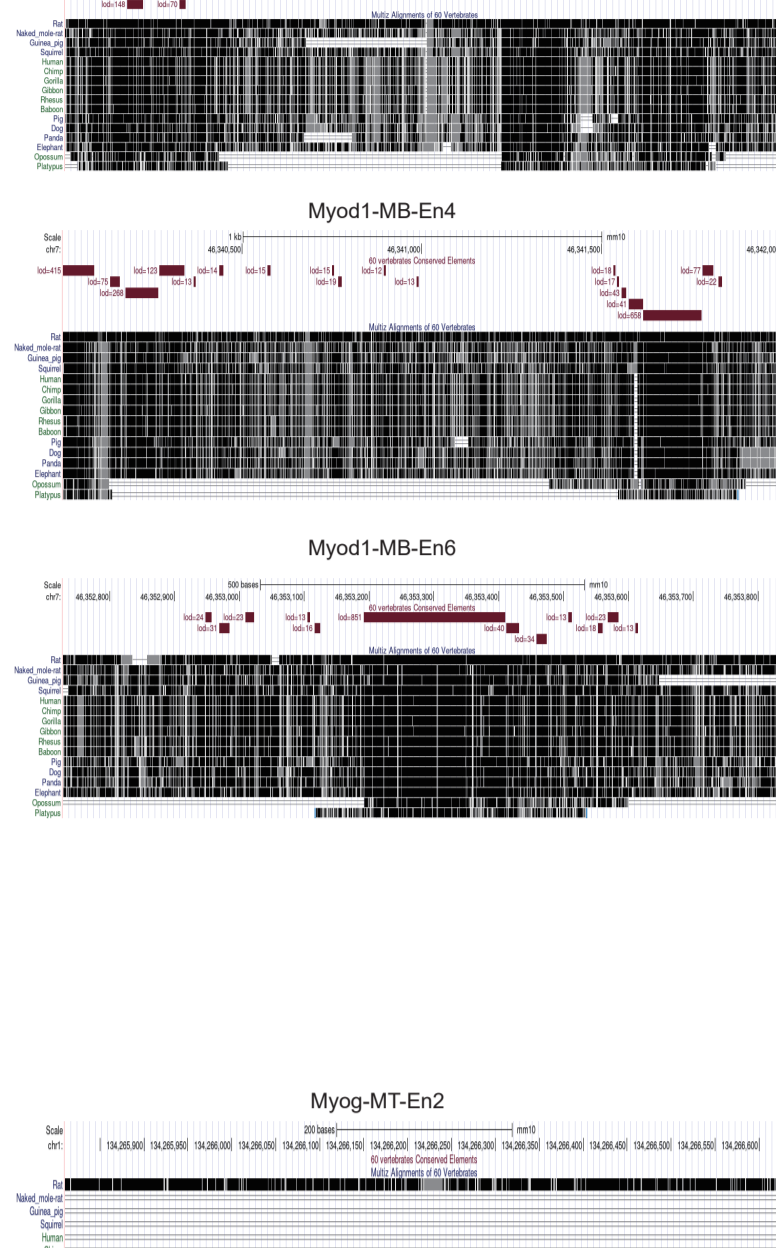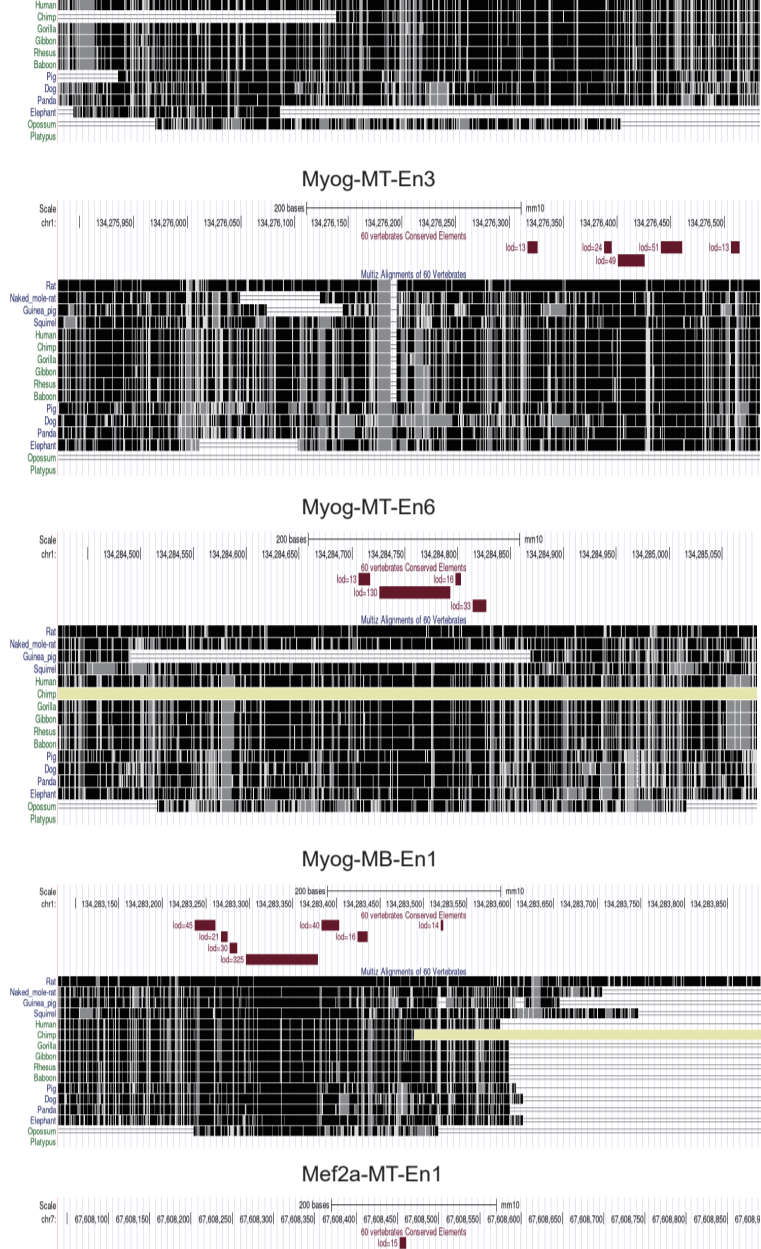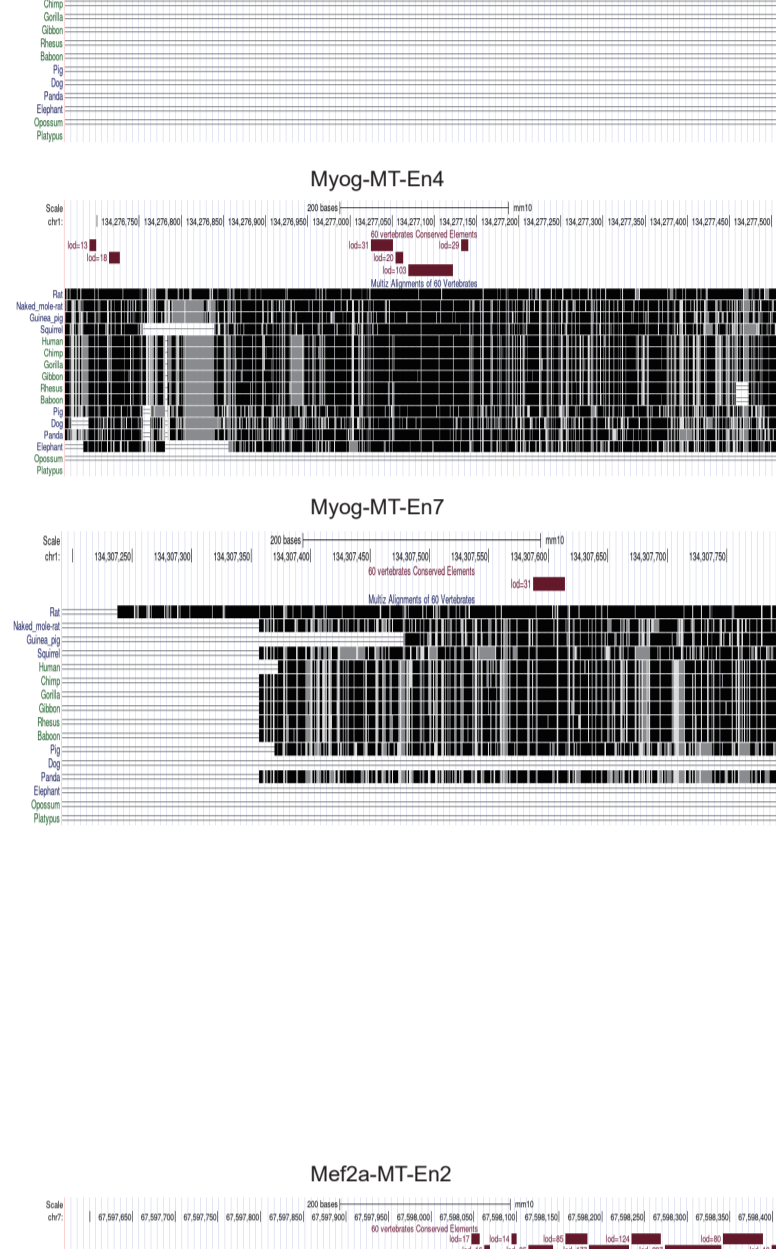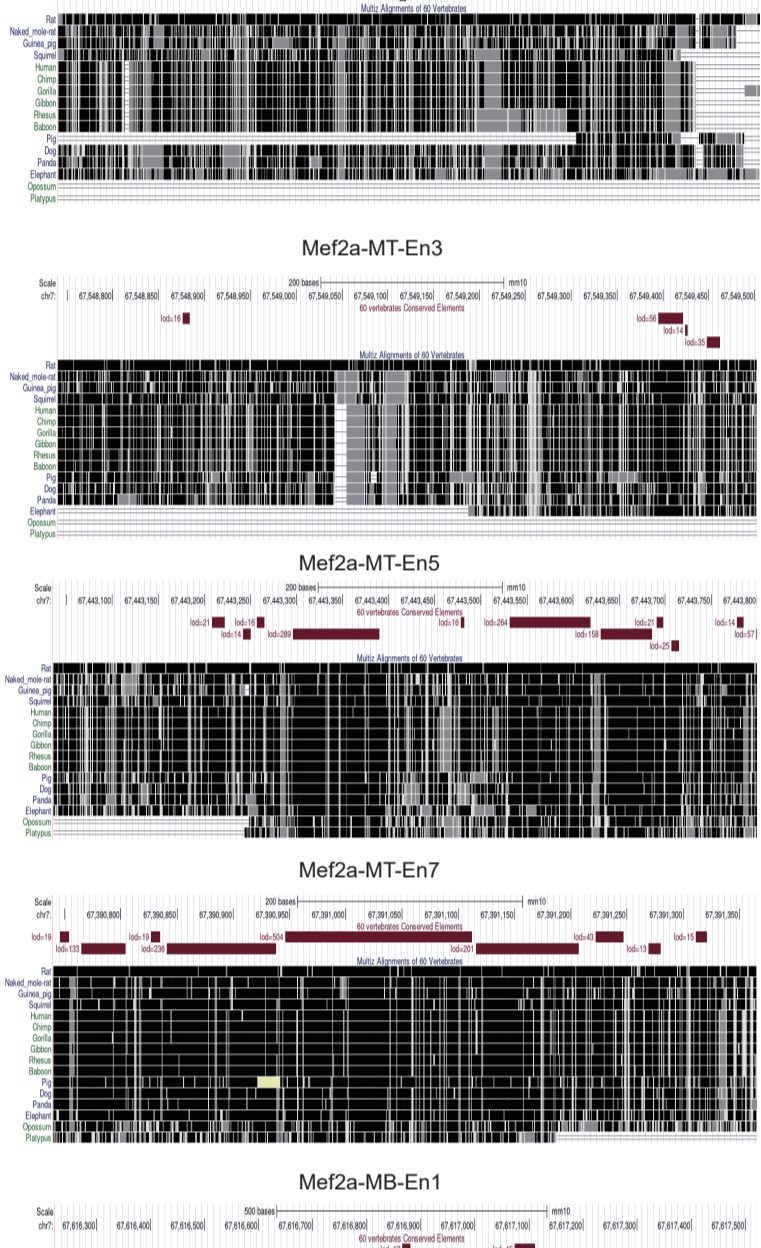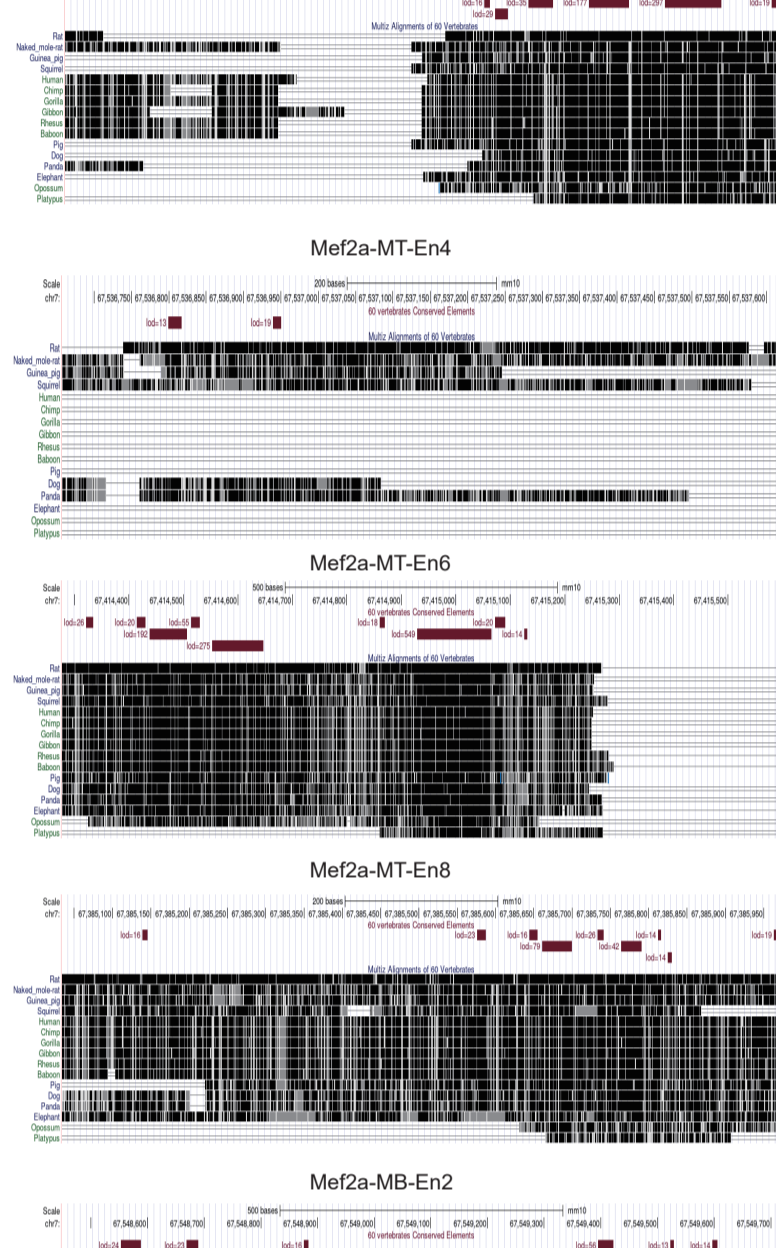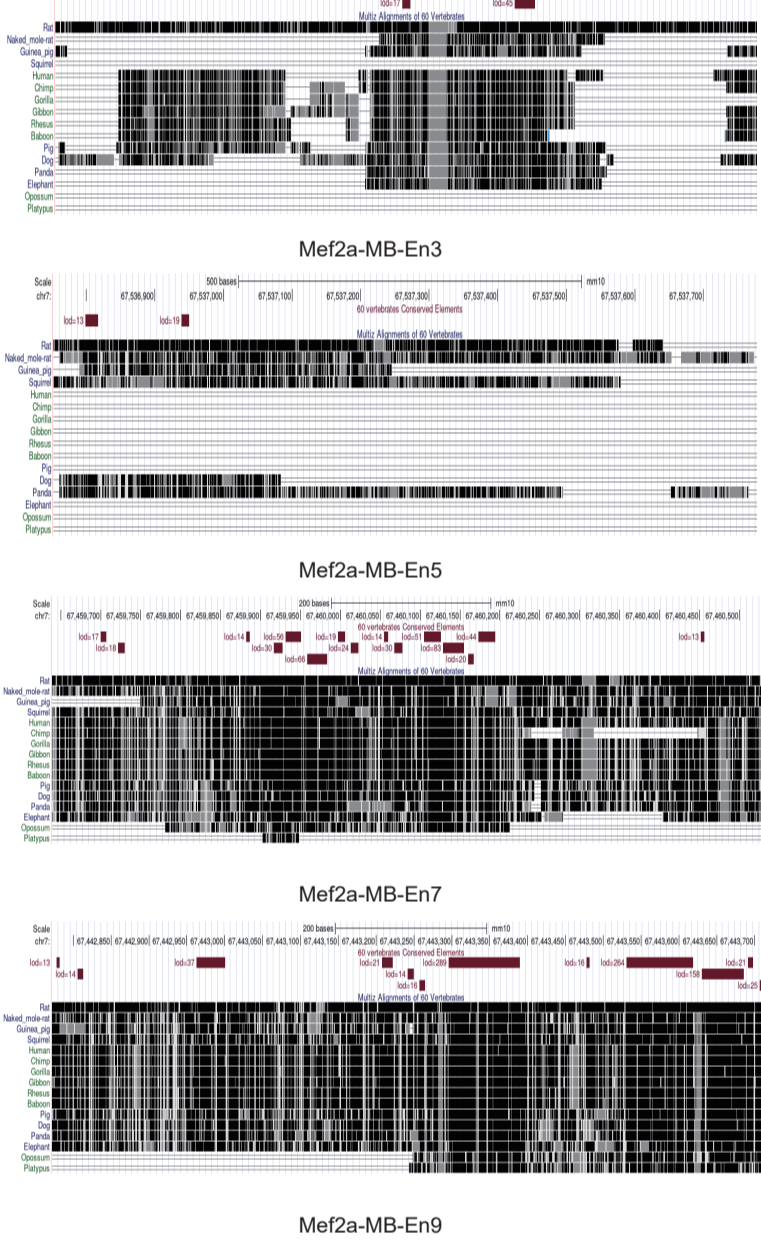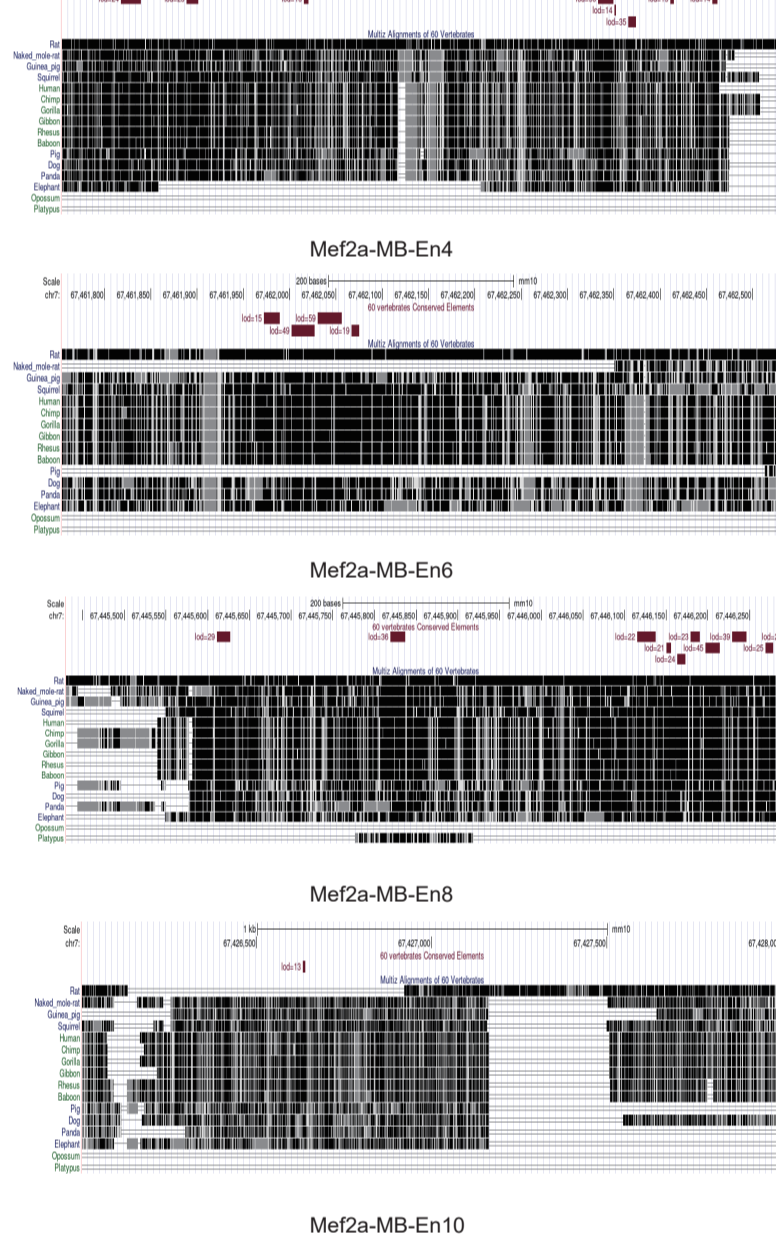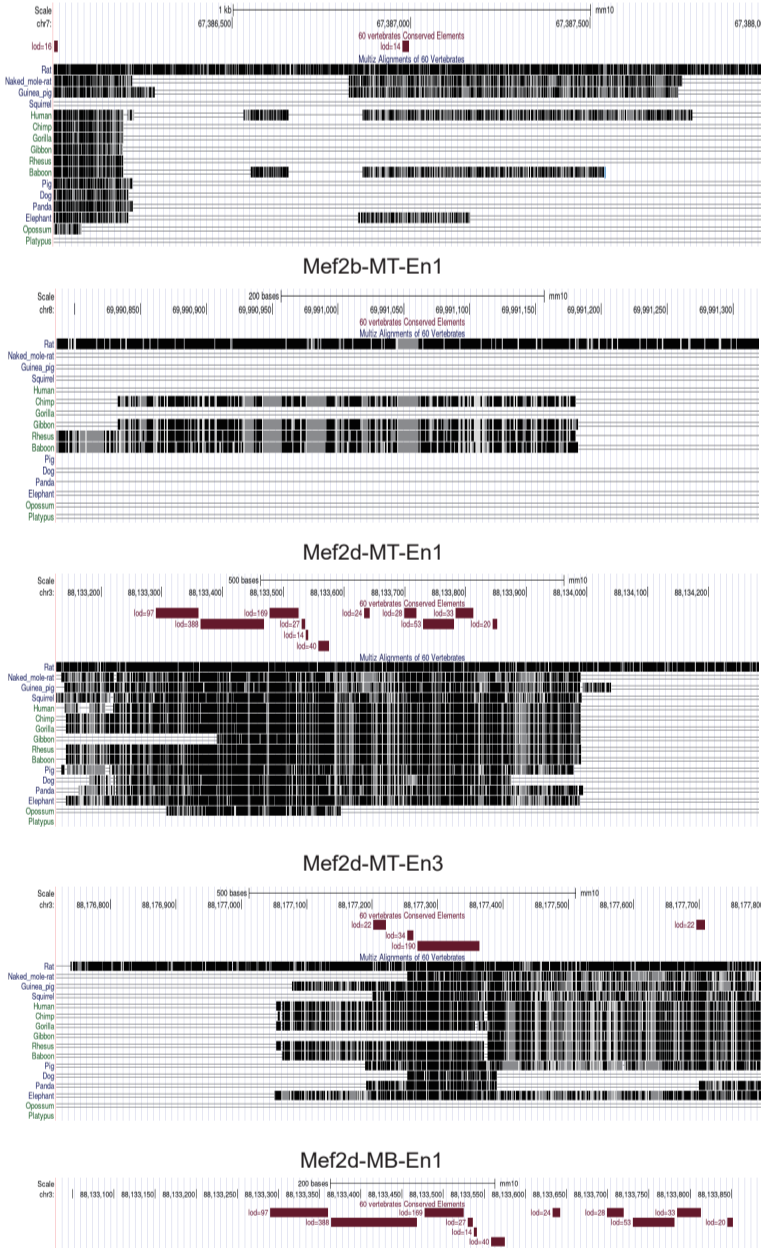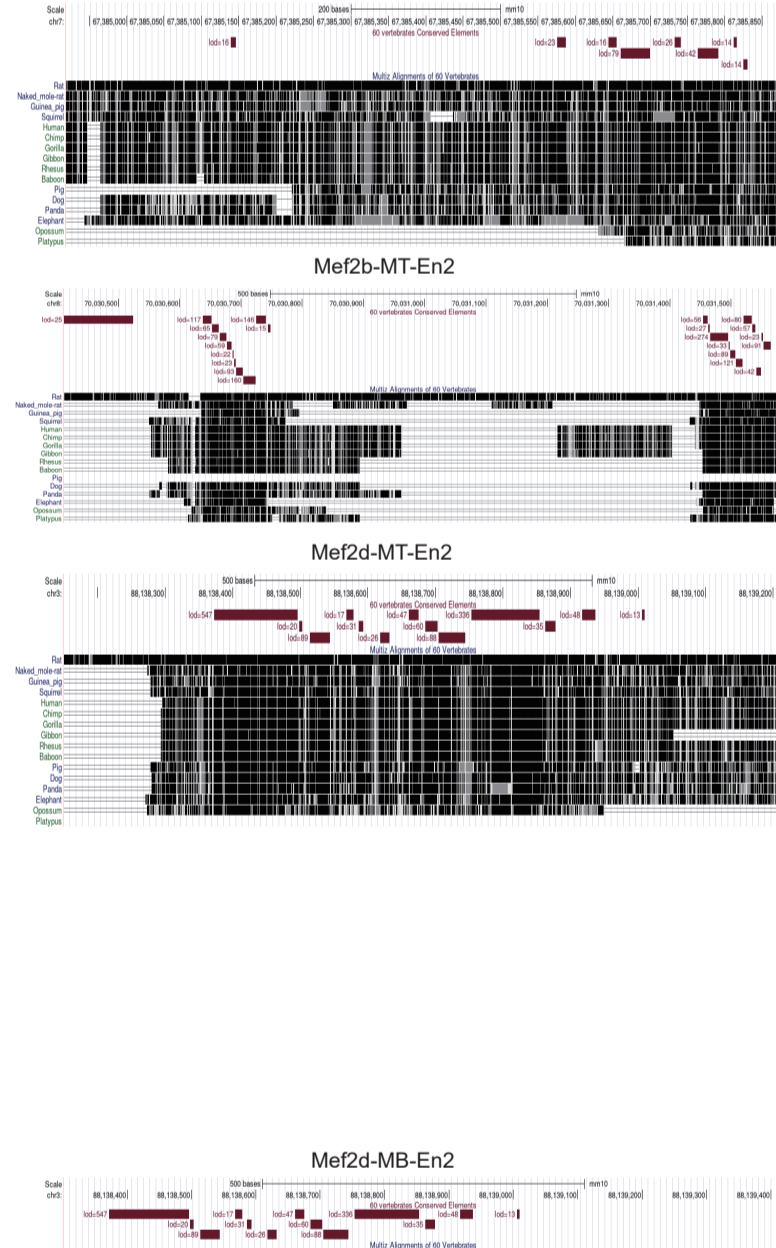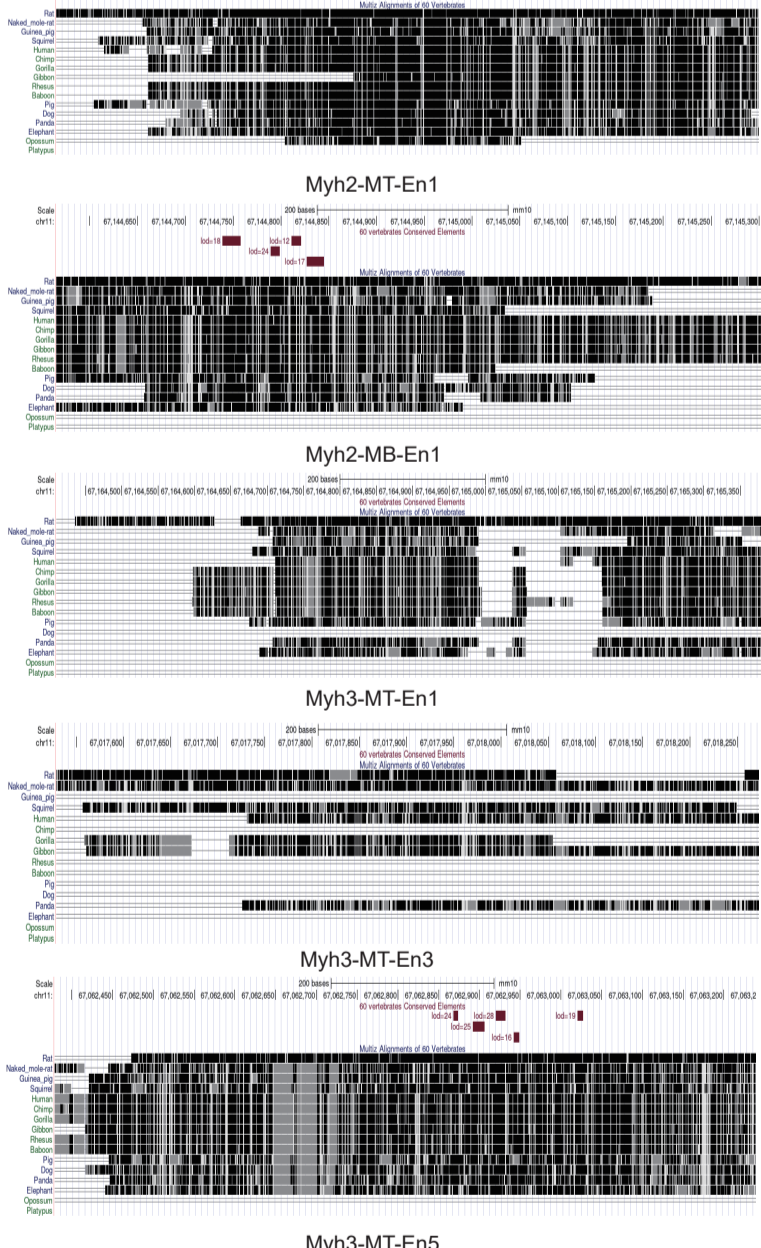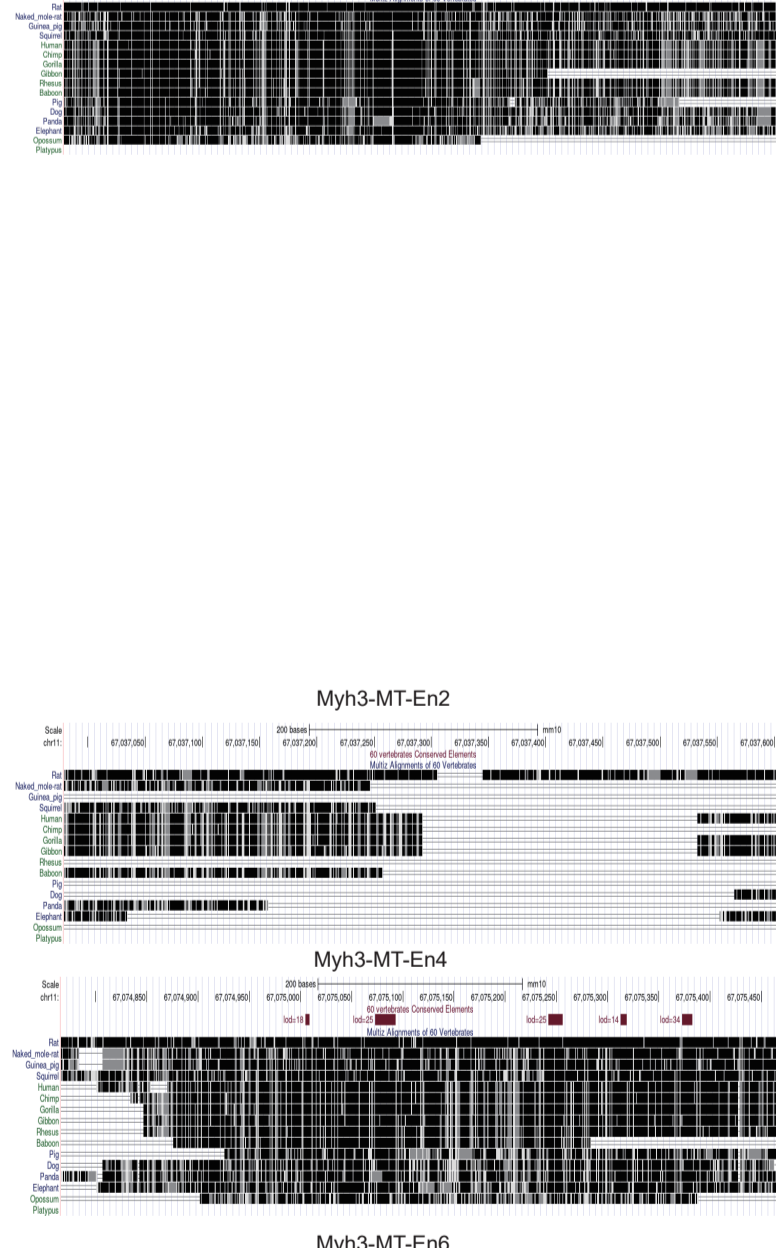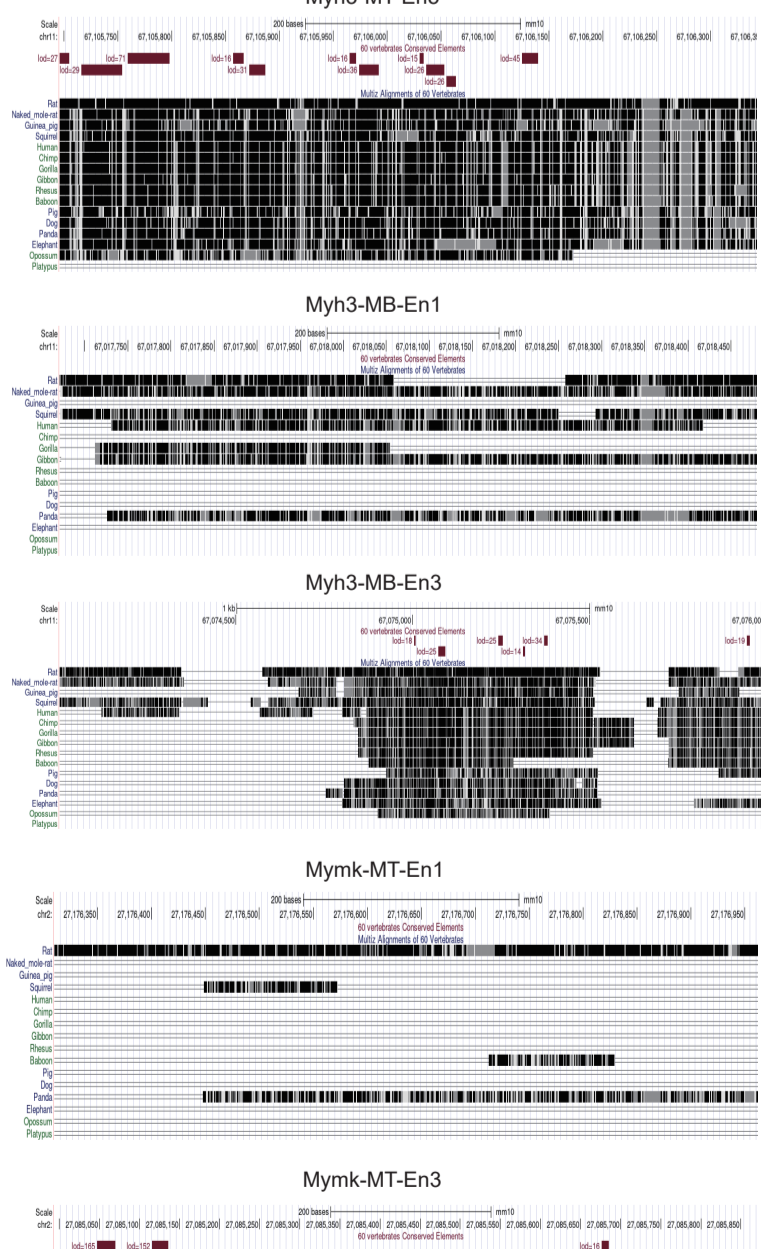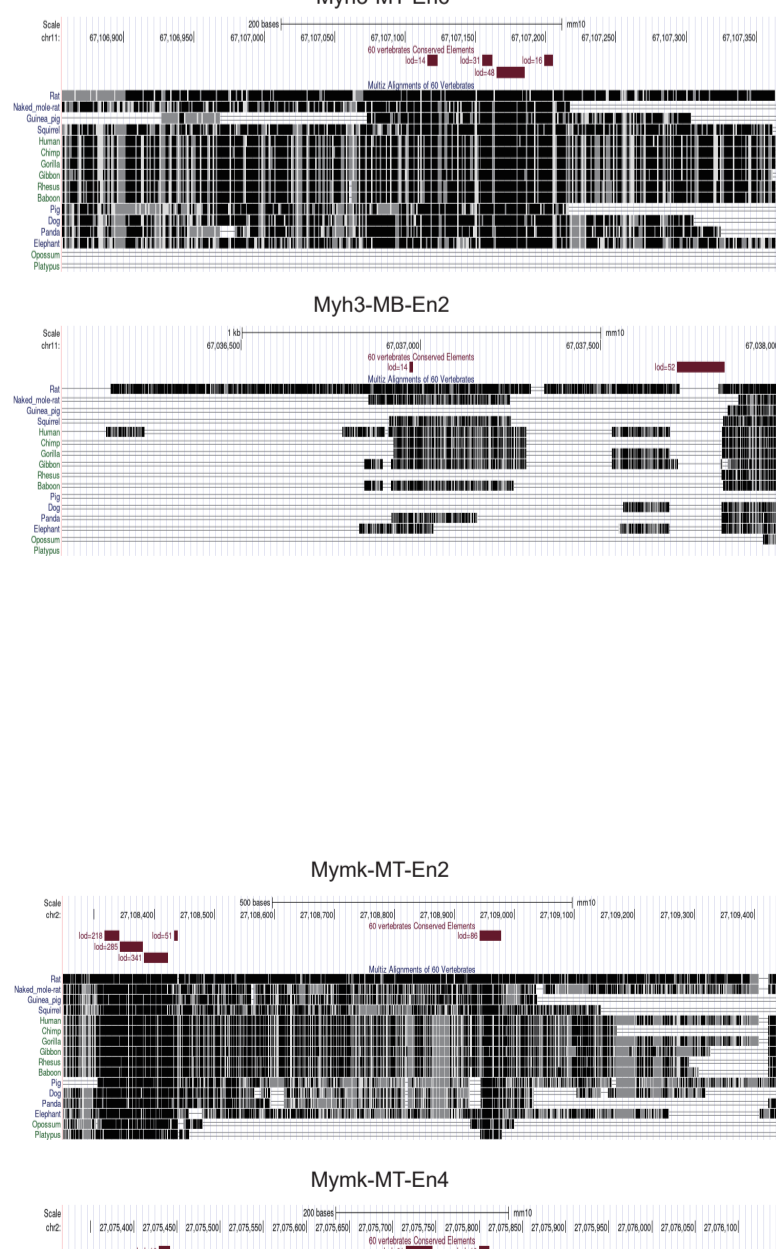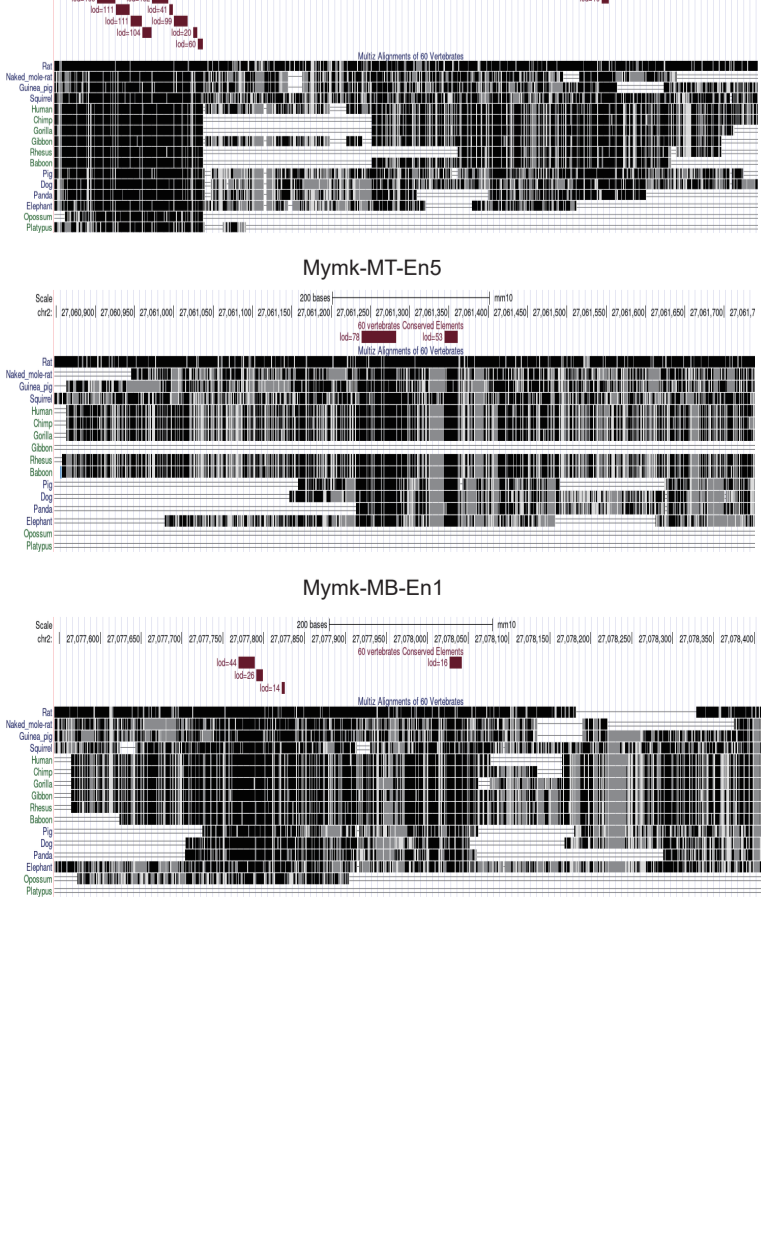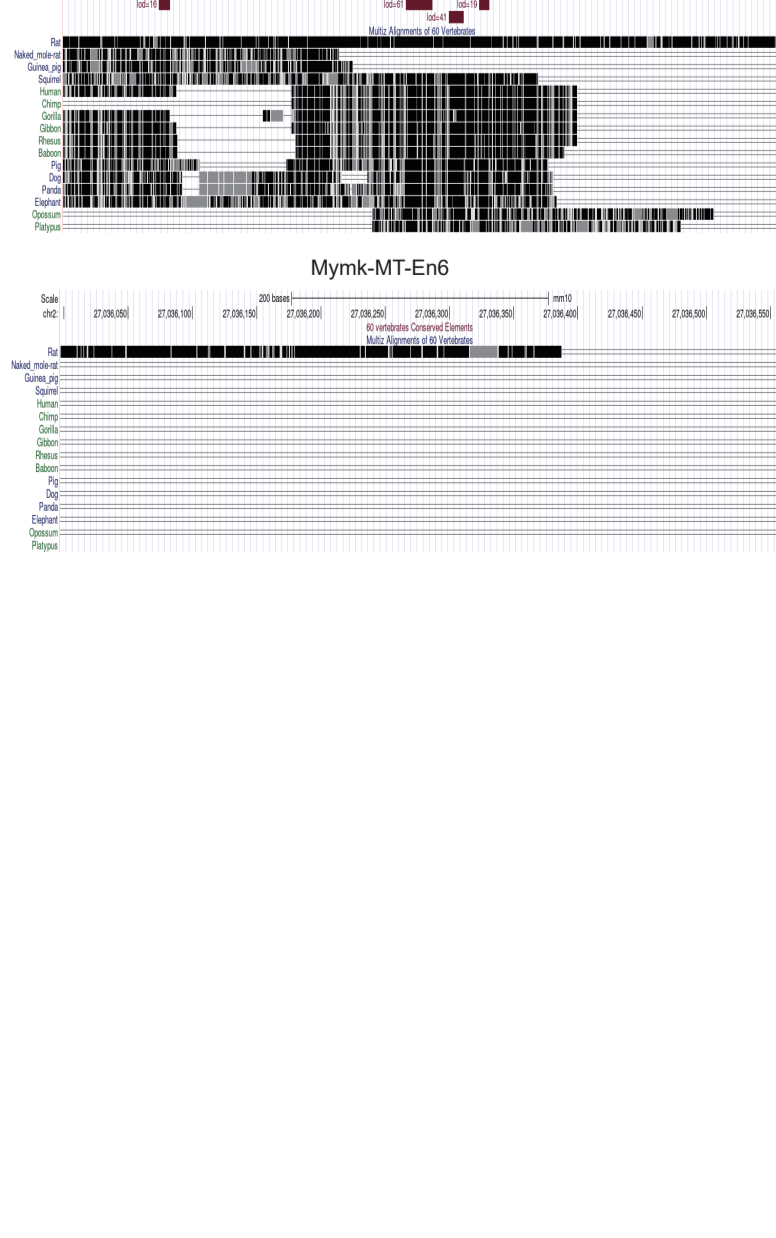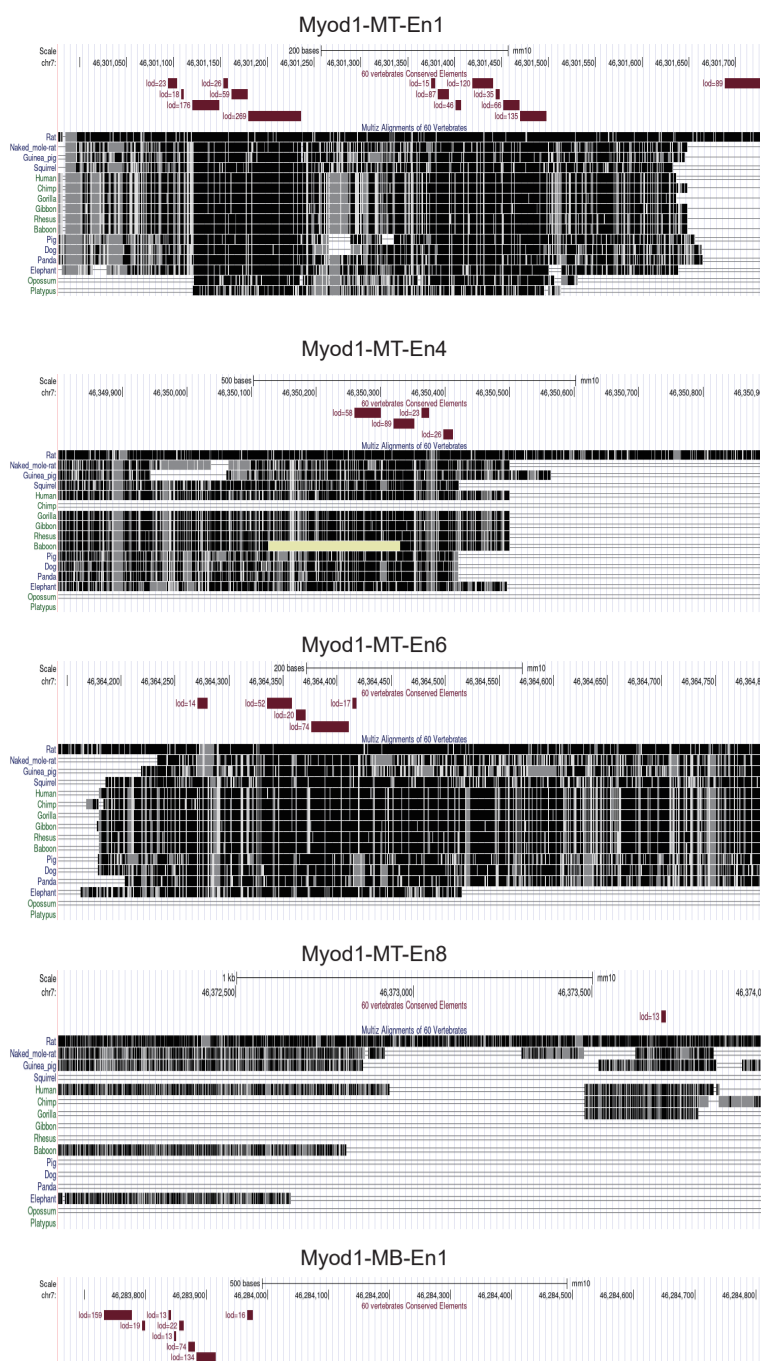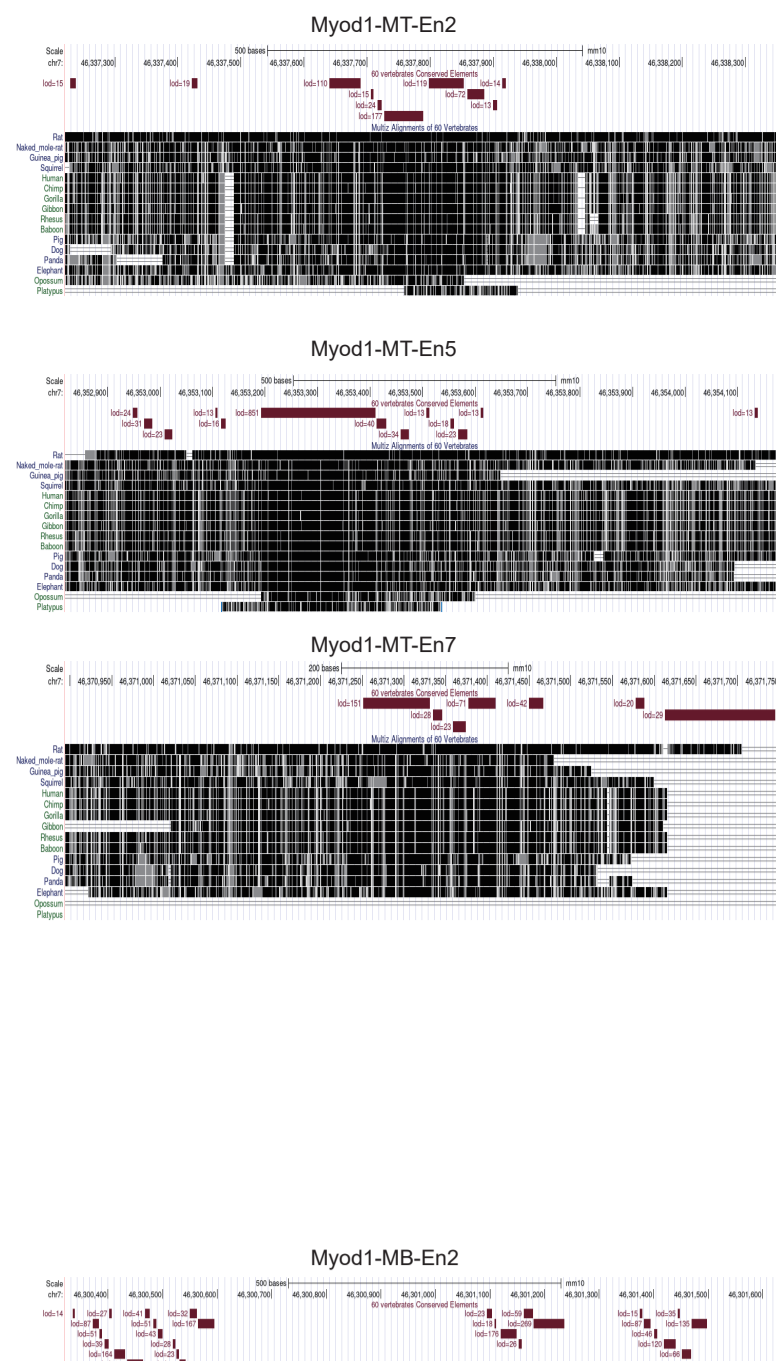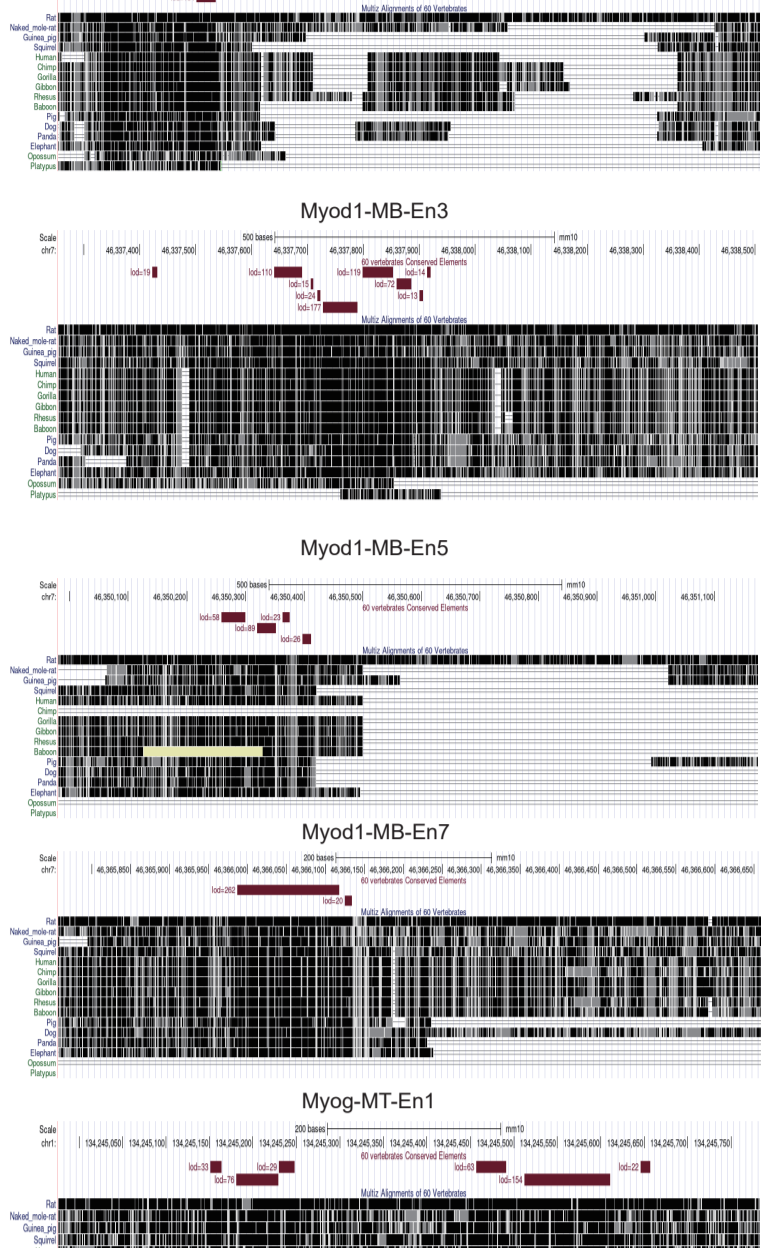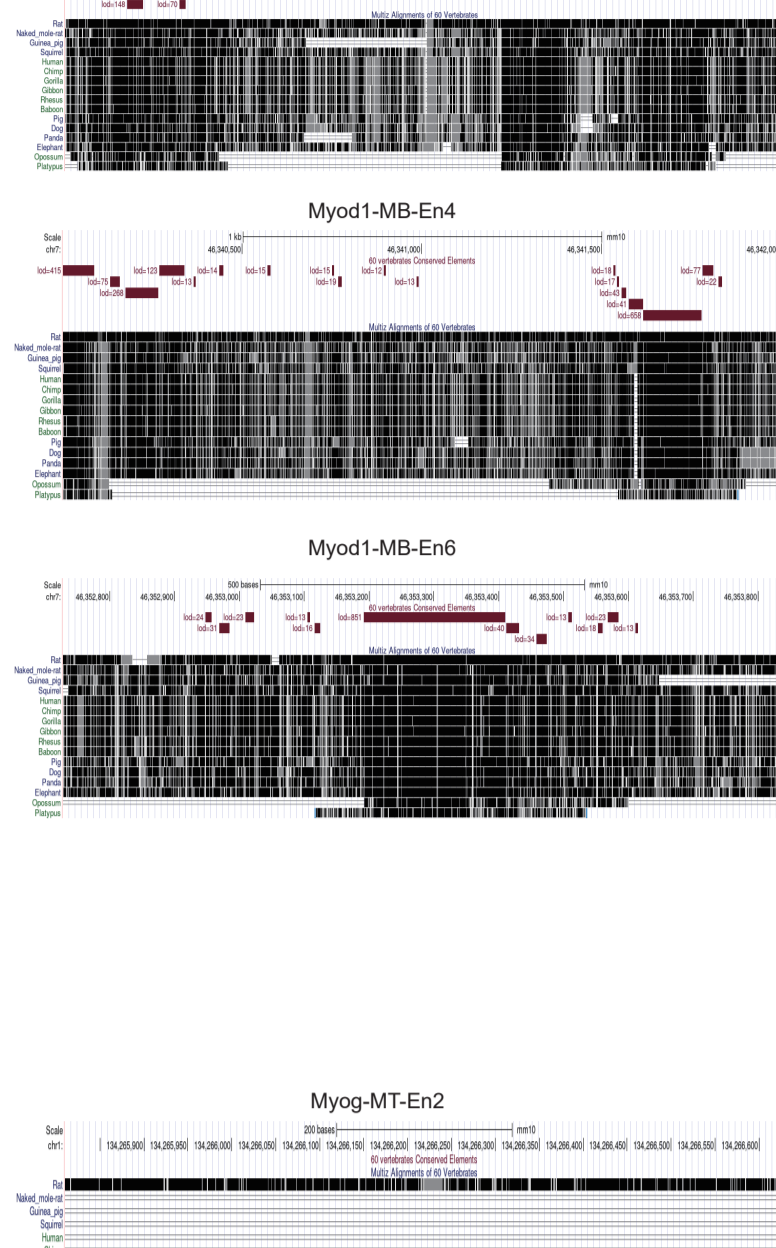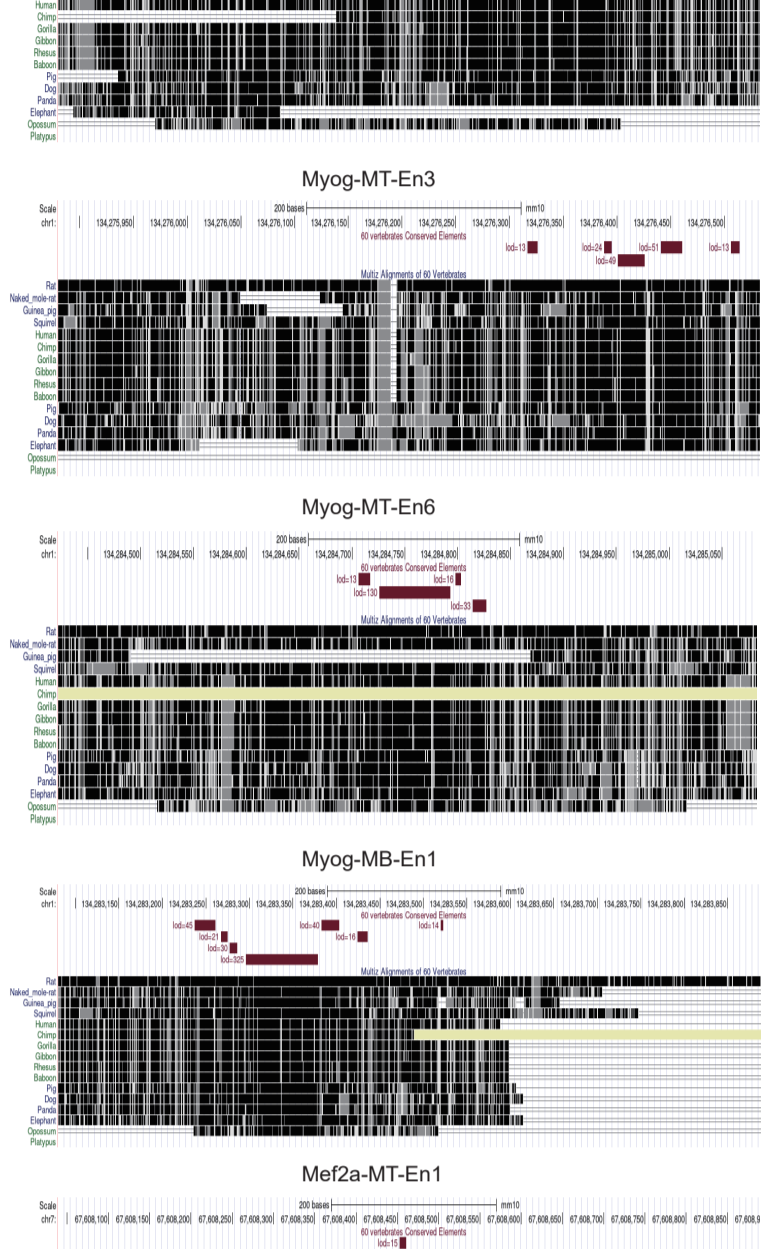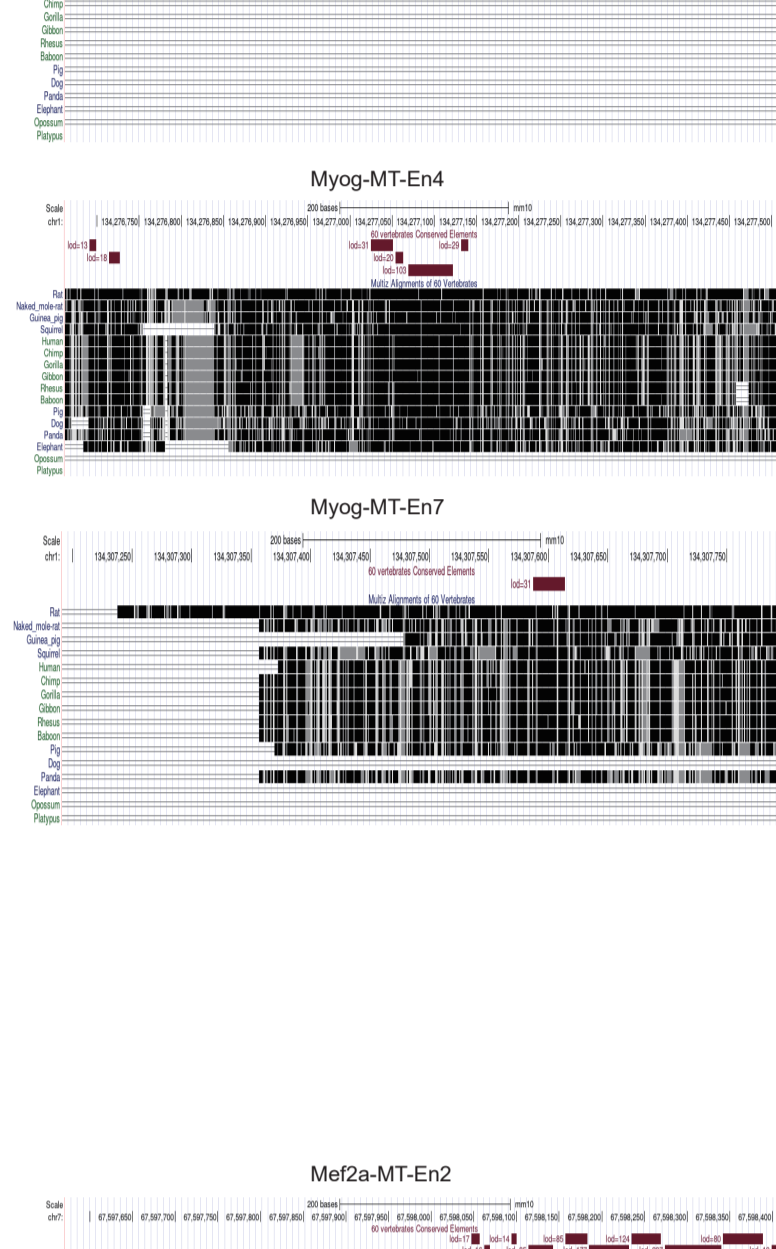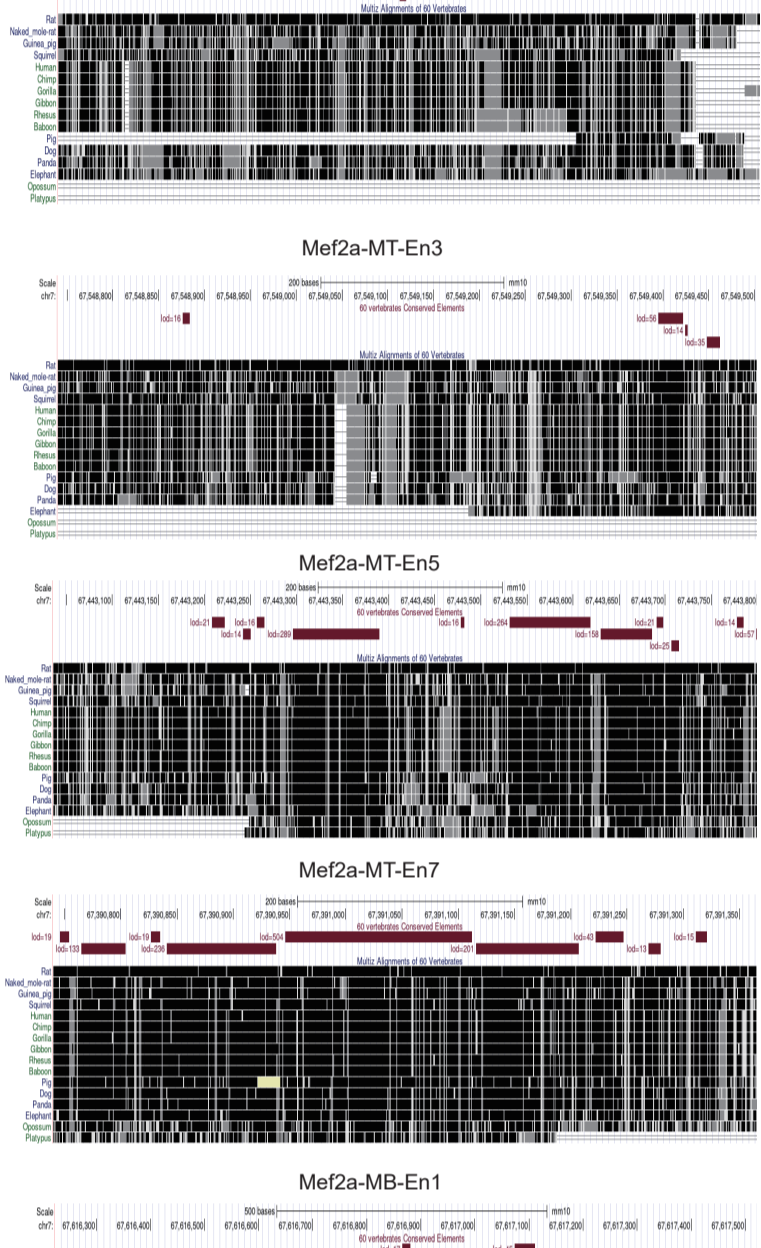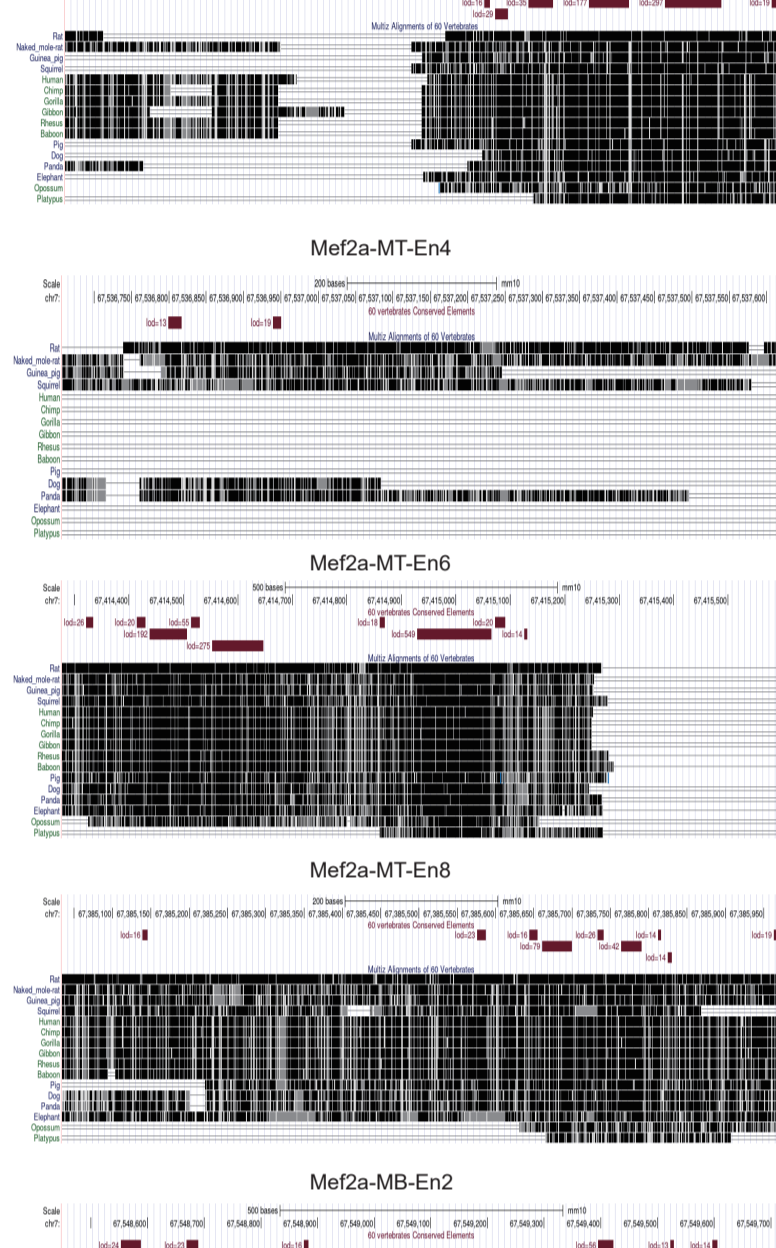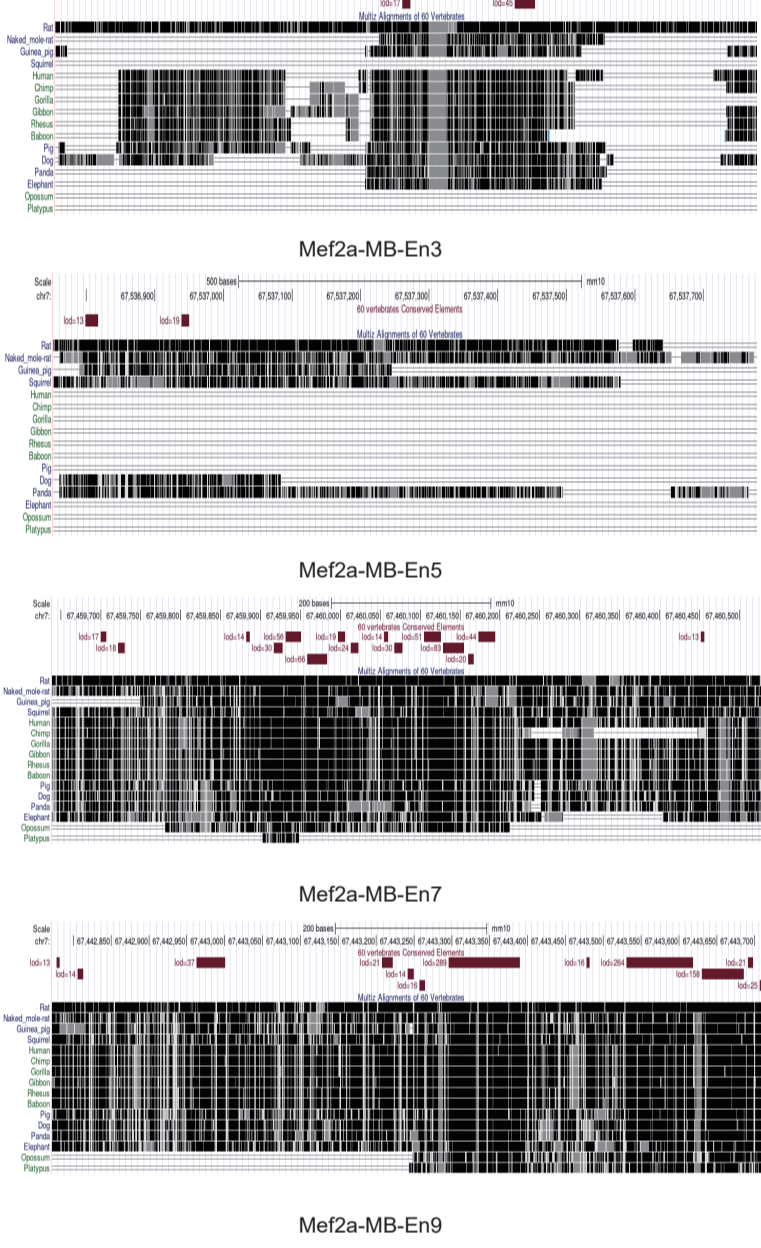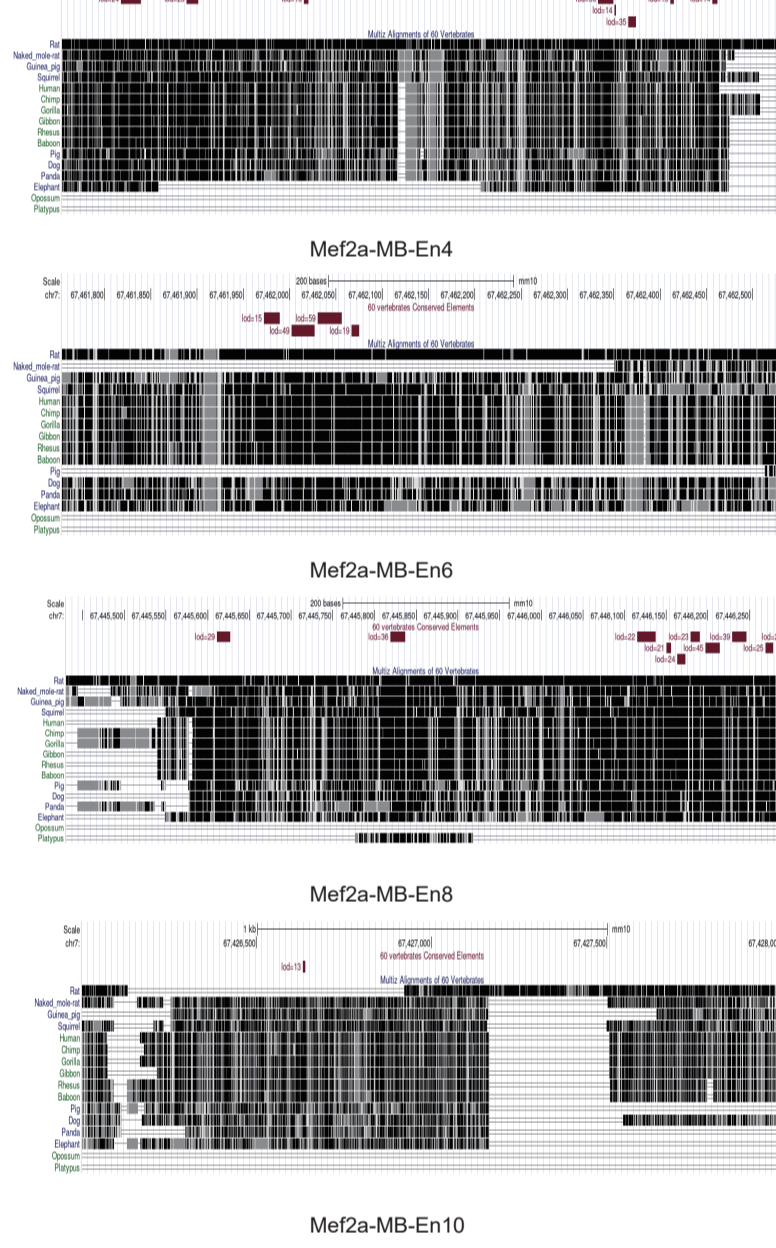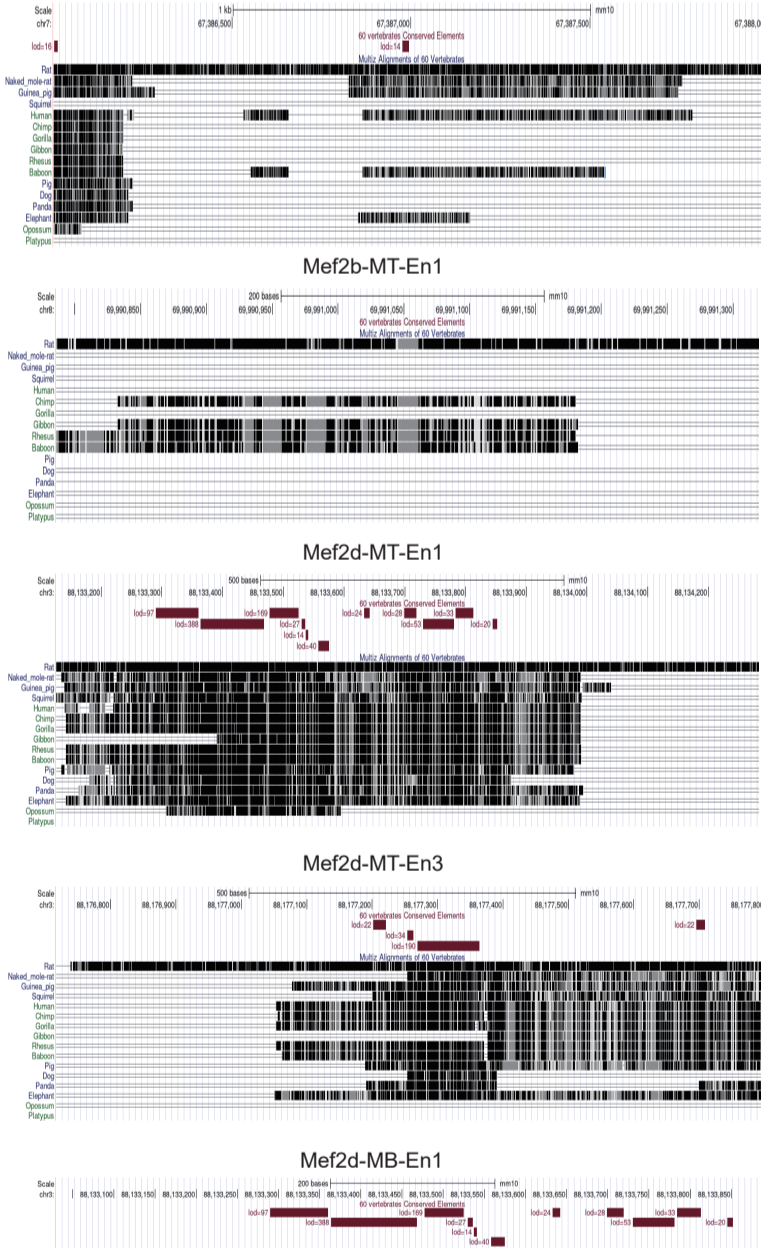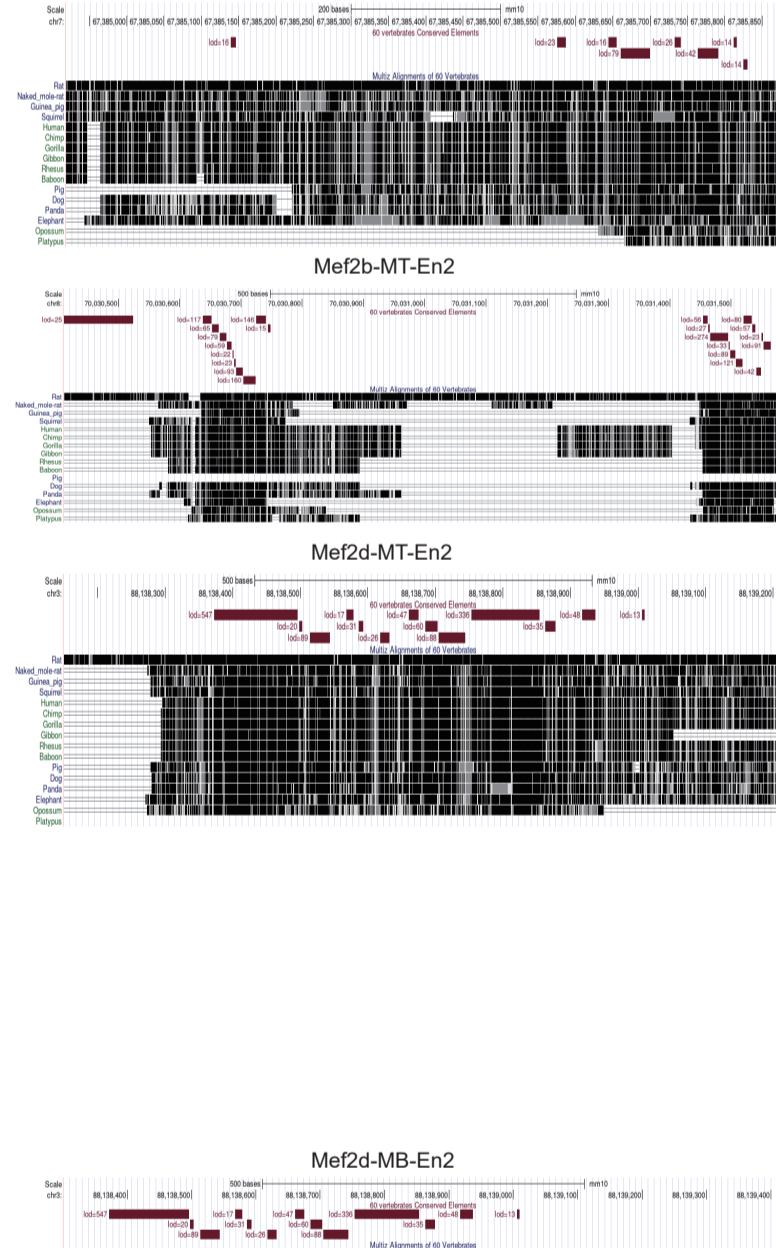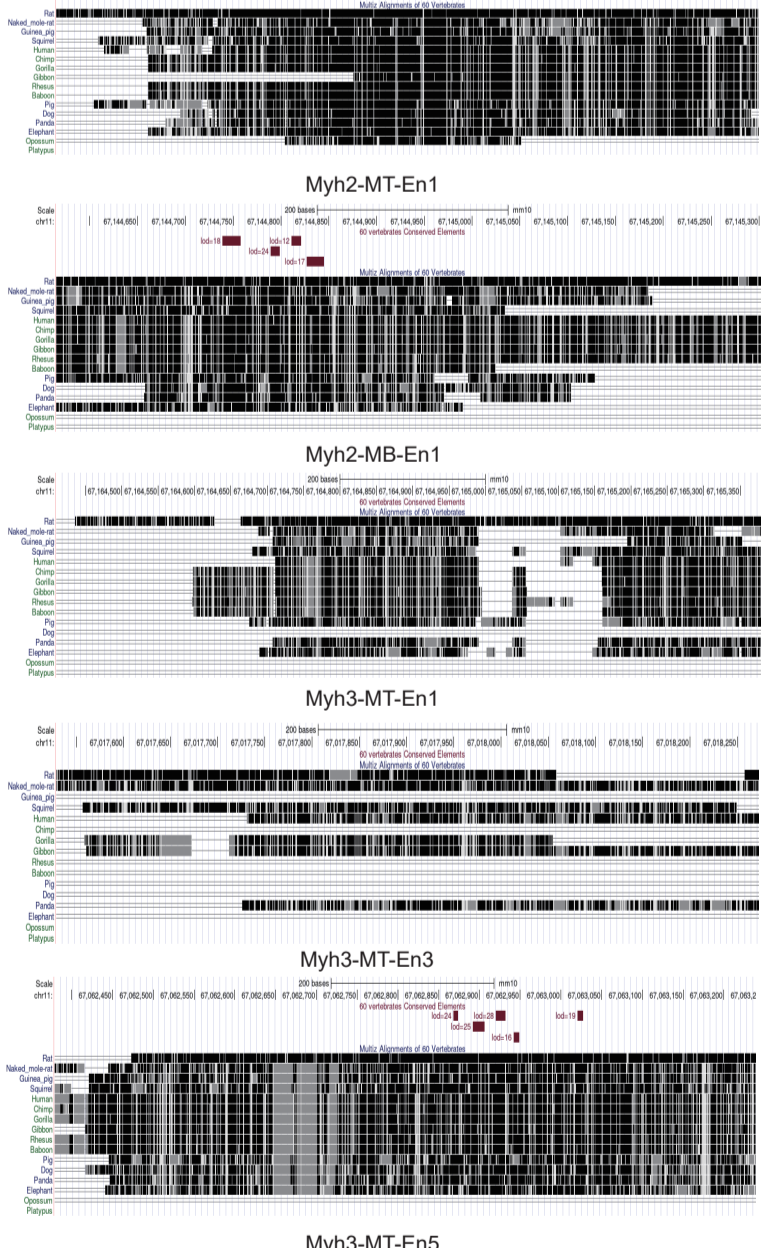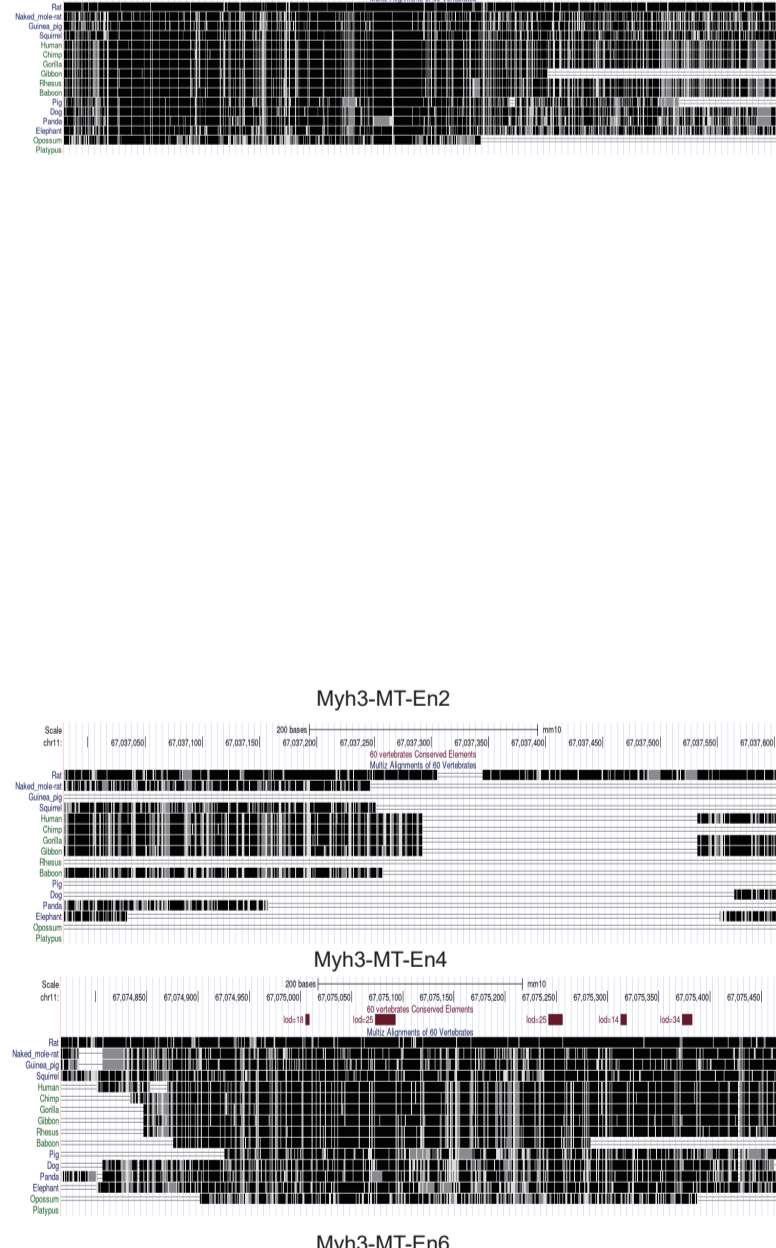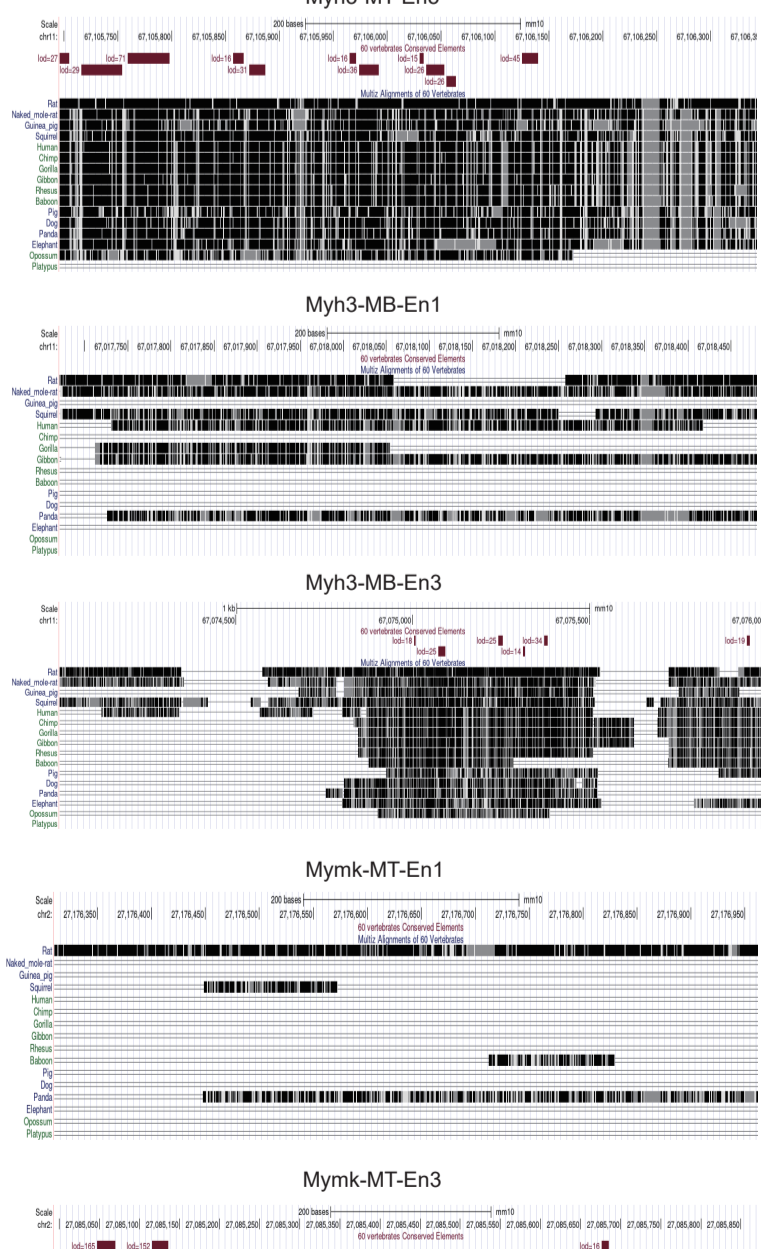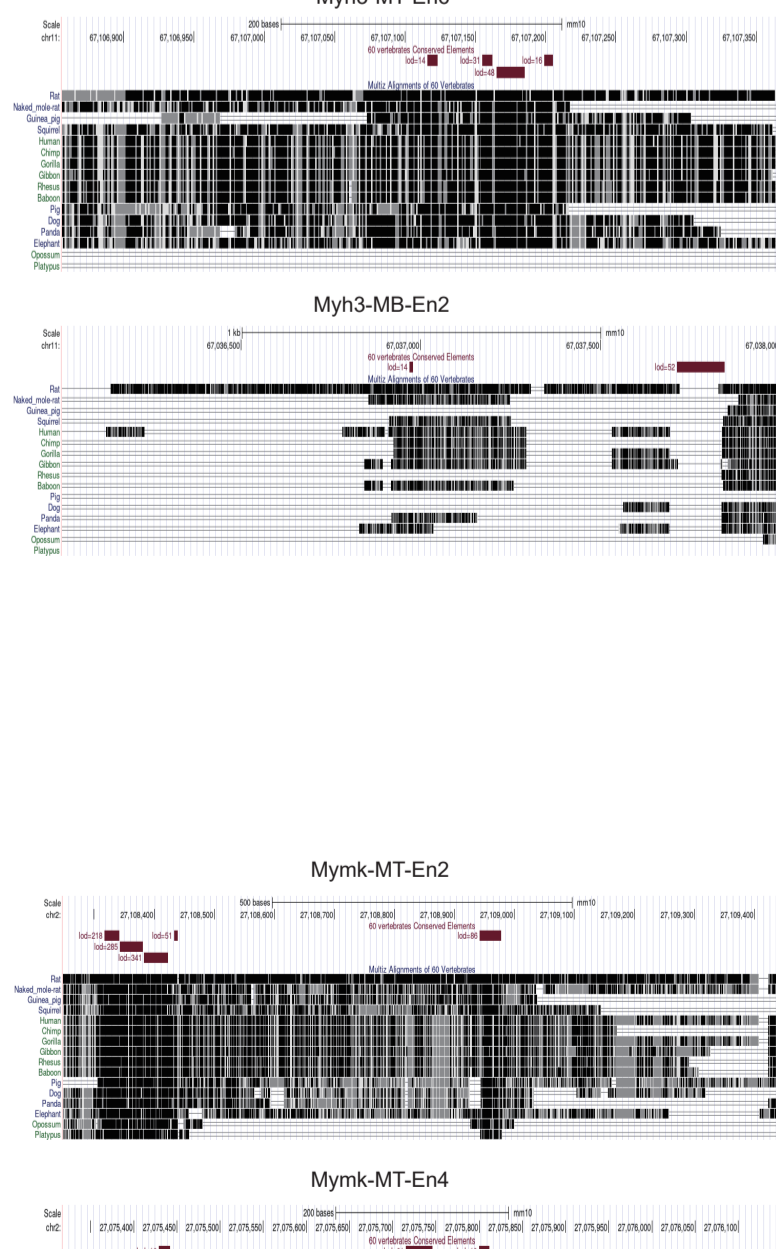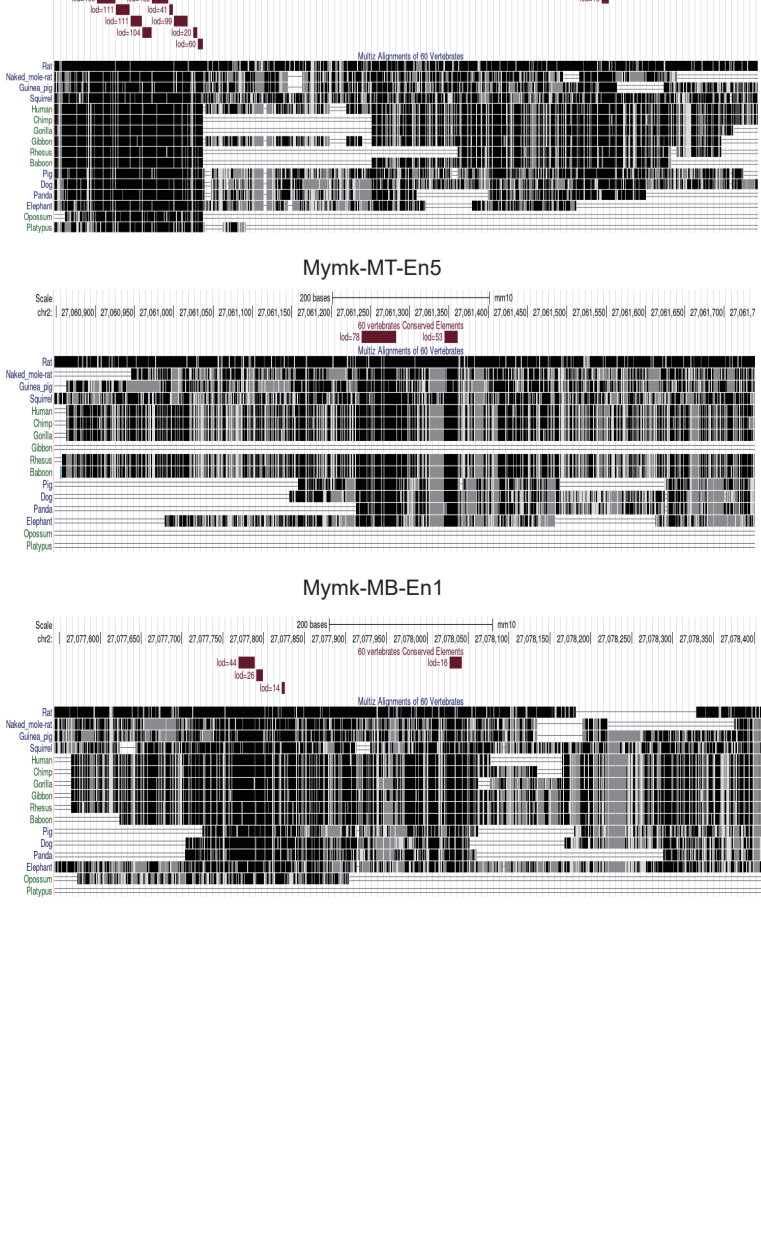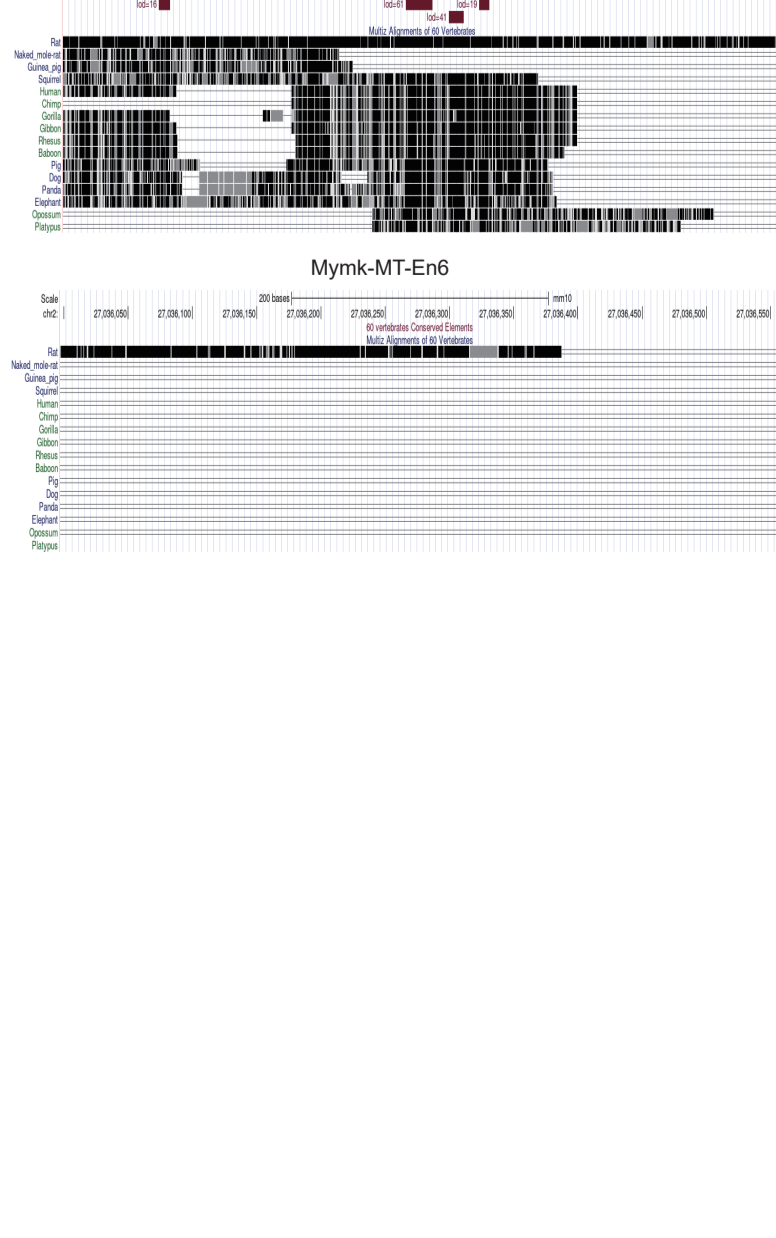

Supplement: Supplementary Table12 [file mmc27.pdf]

# Myog-MT

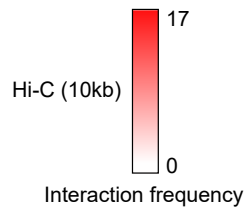

Hi-C C2C12 murine muscle cells  
Barutcu A et al., 2018

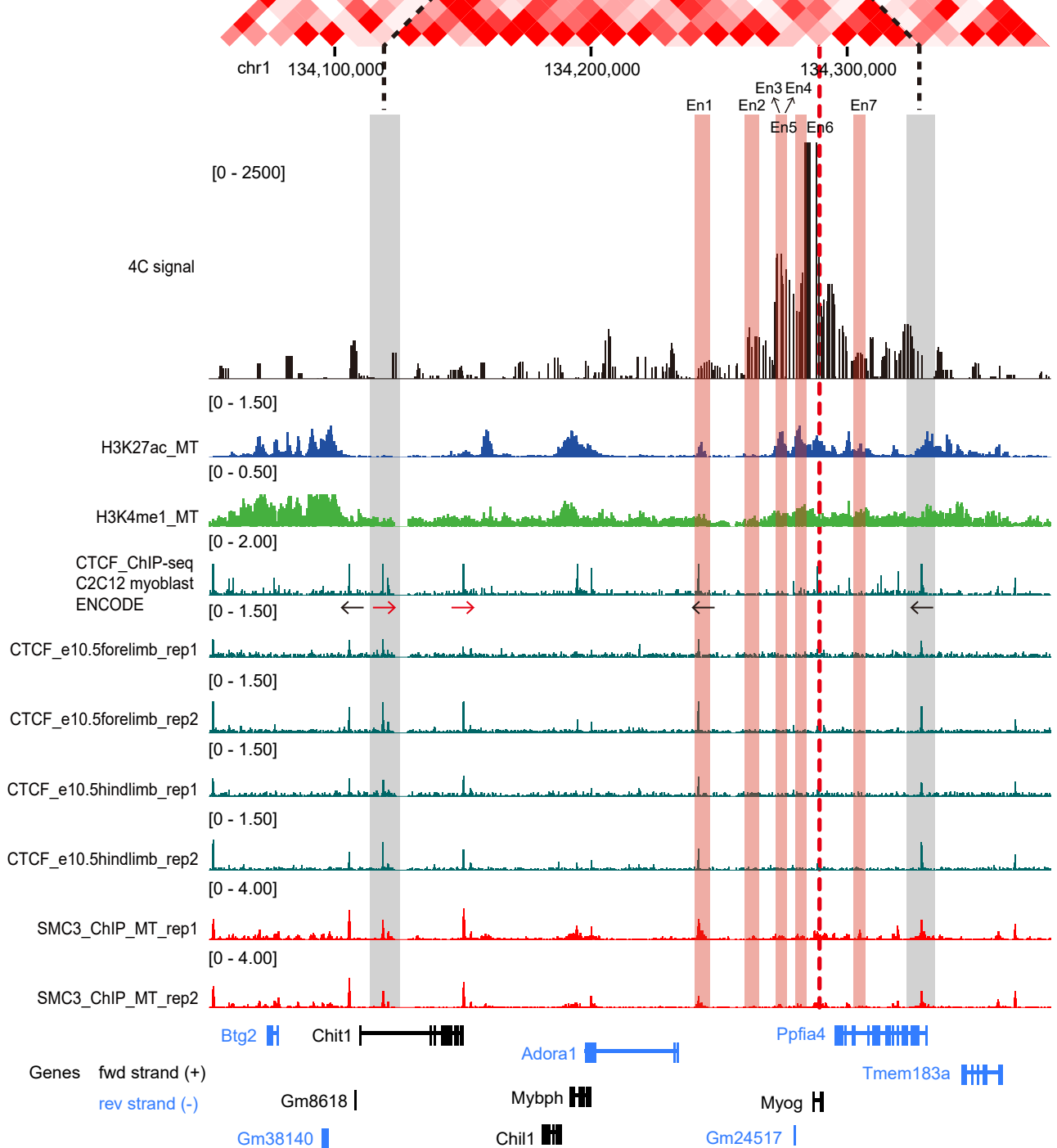

Supplement: Supplementary Table13 [file mmc28.pdf]

**A**

dCas9-KRAB-En1 cells

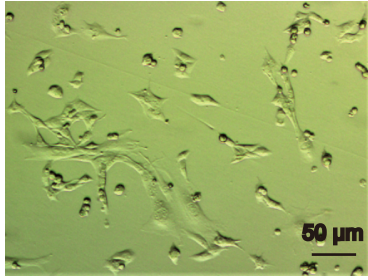

dCas9-KRAB-En3 cells

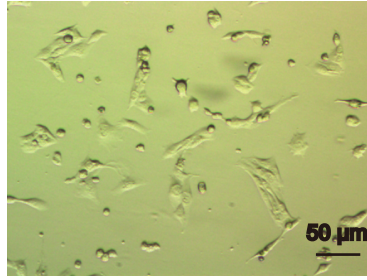

dCas9-KRAB-En5 cells

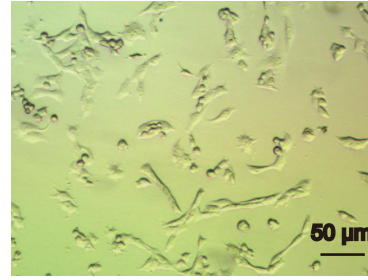**B**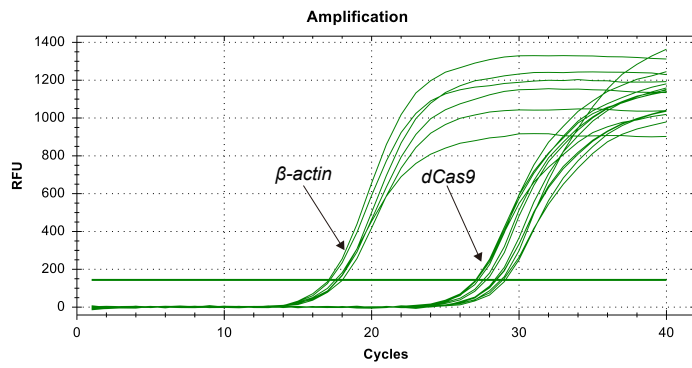**C**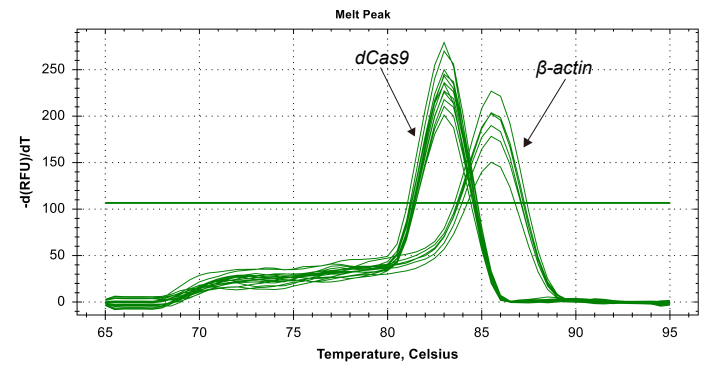

Supplement: Supplementary Table14 [file mmc29.pdf]

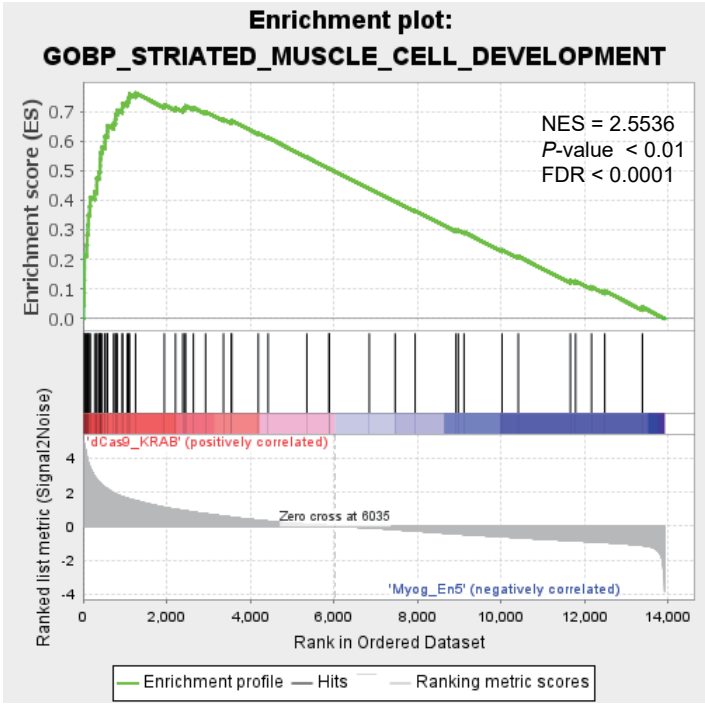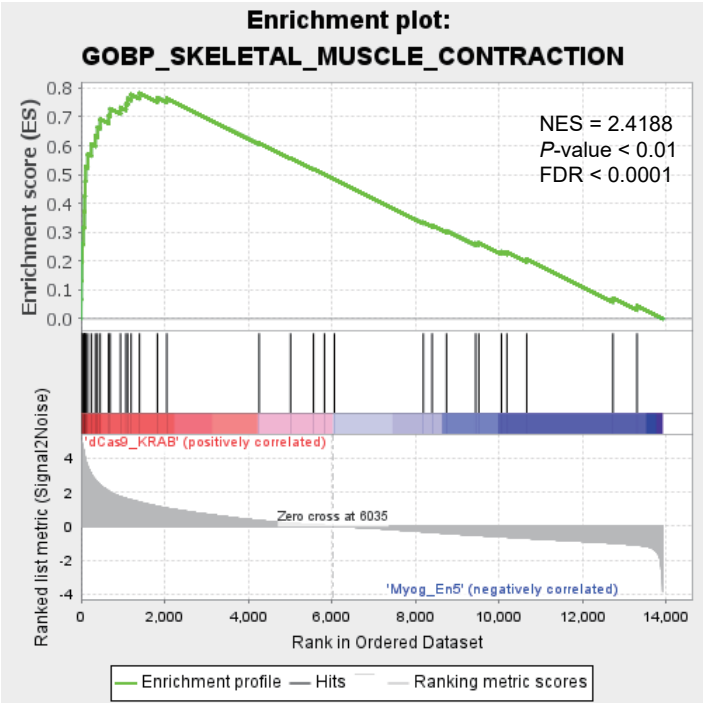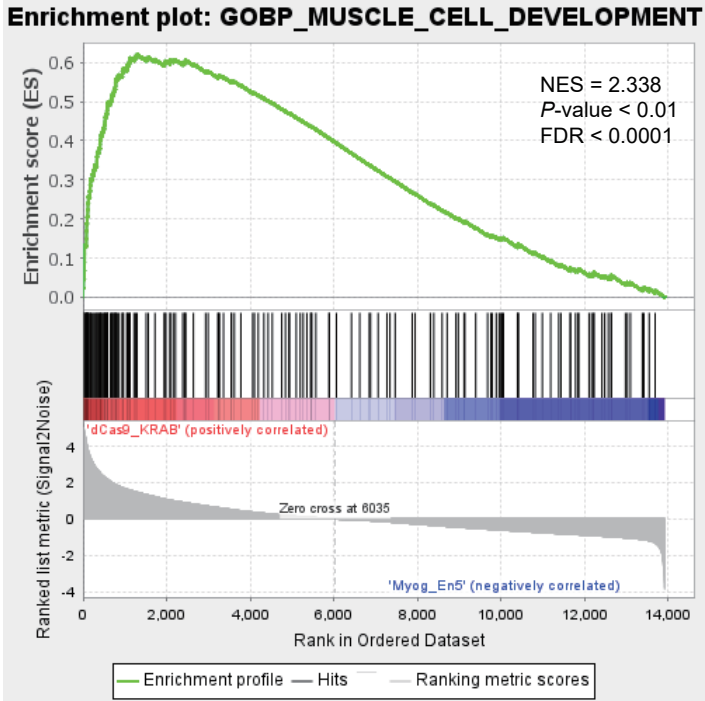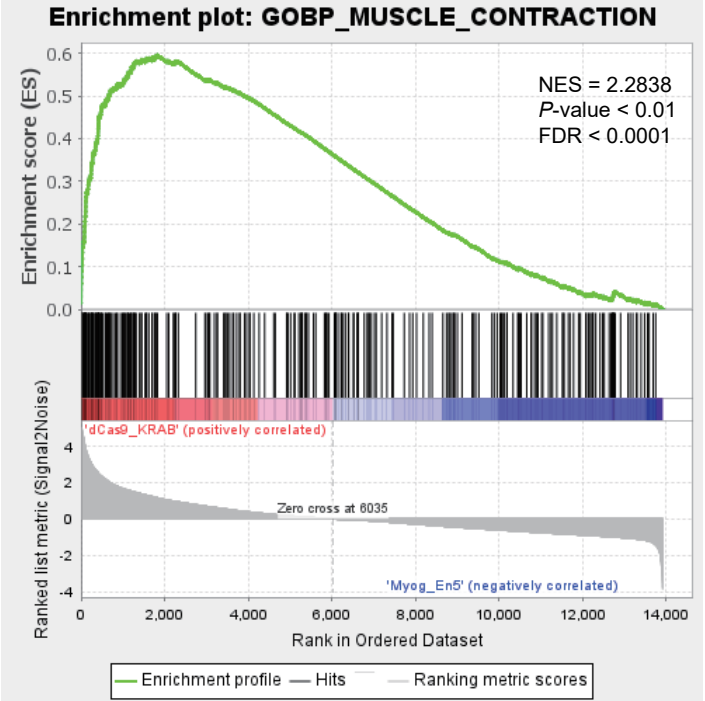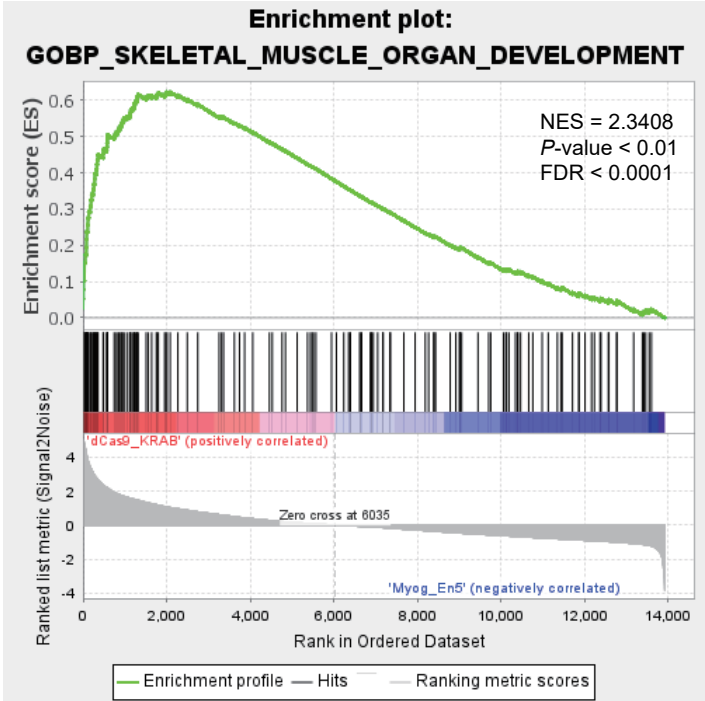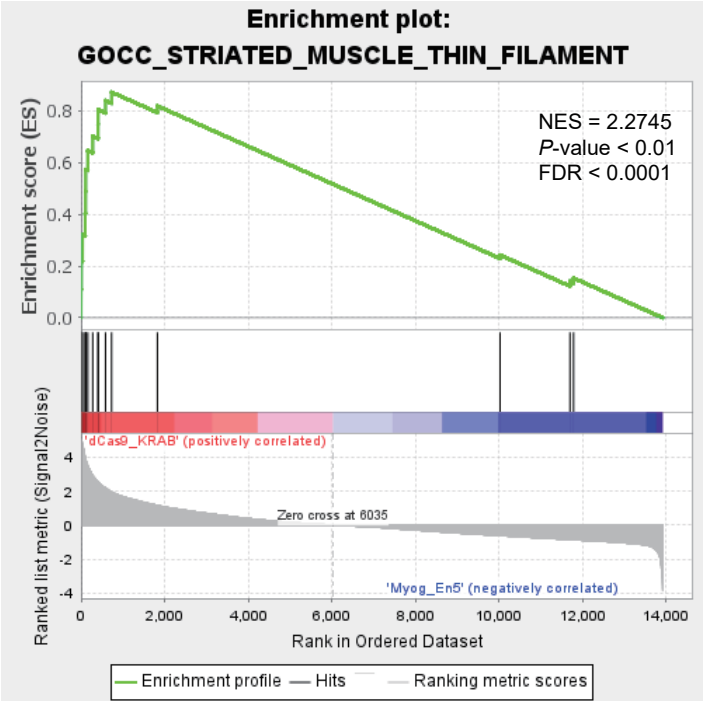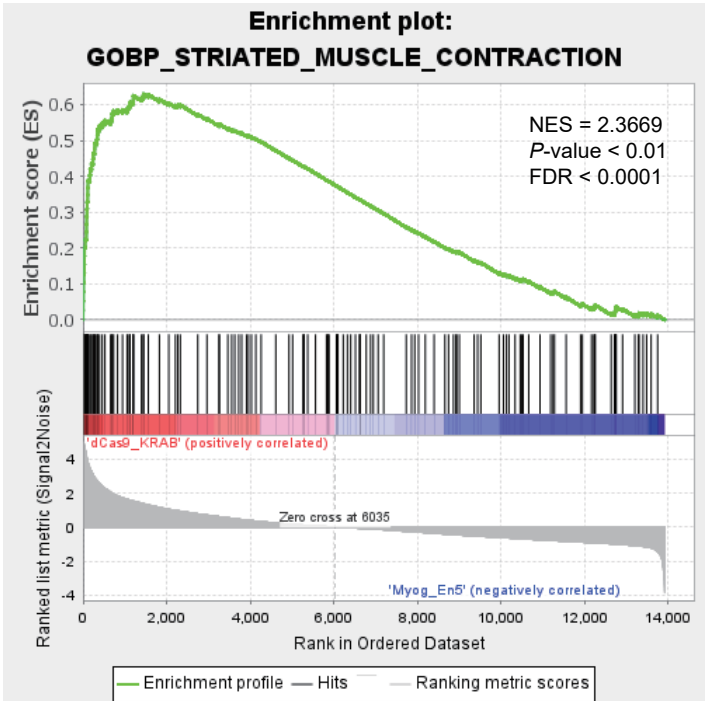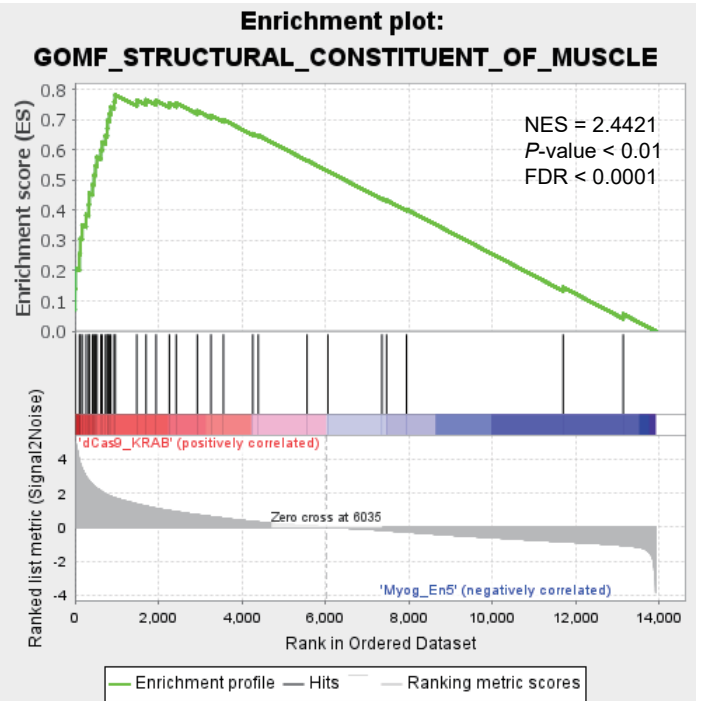

Supplement: Supporting Information Text_Second Submission [file mmc30.pdf]
