# Supplementary material for: Exploring high-resolution chromatin interaction changes and functional enhancers of myogenic marker genes during myogenic differentiation
Source: J Biol Chem. 2022 Jul 2;298(8):102149. doi: 10.1016/j.jbc.2022.102149 (PMC9352921; doi:10.1016/j.jbc.2022.102149)
Supplement: Supplementary Table4 [file mmc19.pdf]

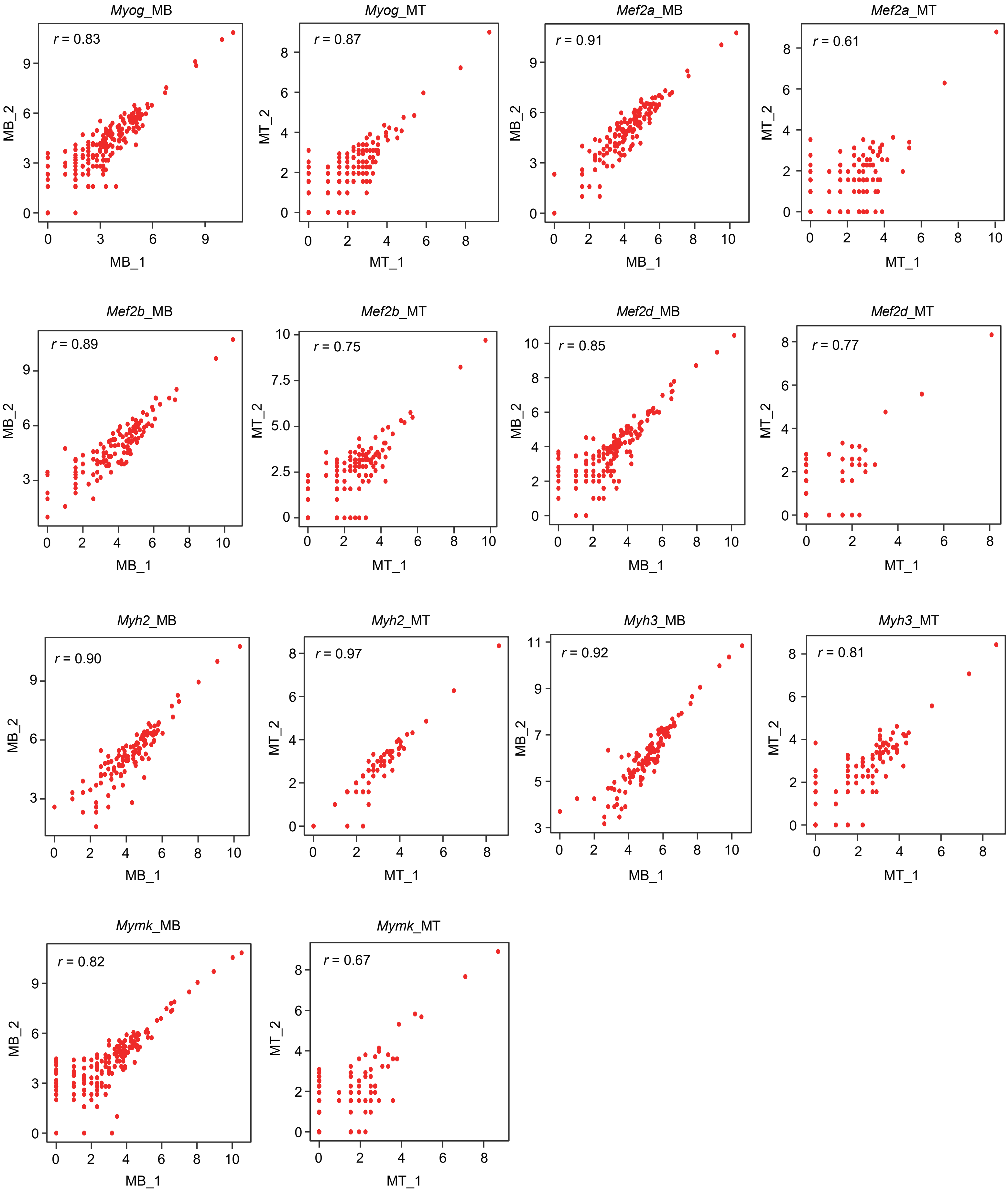

*Myog*_MB
r = 0.83
MB_2
MB_1
*Myog*_MT
r = 0.87
MT_2
MT_1
*Mef2a*_MB
r = 0.91
MB_2
MB_1
*Mef2a*_MT
r = 0.61
MT_2
MT_1
*Mef2b*_MB
r = 0.89
MB_2
MB_1
*Mef2b*_MT
r = 0.75
MT_2
MT_1
*Mef2d*_MB
r = 0.85
MB_2
MB_1
*Mef2d*_MT
r = 0.77
MT_2
MT_1
*Myh2*_MB
r = 0.90
MB_2
MB_1
*Myh2*_MT
r = 0.97
MT_2
MT_1
*Myh3*_MB
r = 0.92
MB_2
MB_1
*Myh3*_MT
r = 0.81
MT_2
MT_1
*Mymk*_MB
r = 0.82
MB_2
MB_1
*Mymk*_MT
r = 0.67
MT_2
MT_1
